# Enrichment of Antibiotic Resistance Genes on Plastic Waste in Aquatic Ecosystems, Aquatic Animals, and Fishery Products

**DOI:** 10.3390/antibiotics14111106

**Published:** 2025-11-02

**Authors:** Franca Rossi, Serena Santonicola, Giampaolo Colavita

**Affiliations:** 1 Istituto Zooprofilattico Sperimentale dell’Abruzzo e Molise (IZSAM), 86100 Campobasso, Italy; 2 Dipartimento di Medicina e Scienze Della Salute “V. Tiberio”, Università Degli Studi del Molise, 86100 Campobasso, Italy; serena.santonicola@unimol.it (S.S.); colavita@unimol.it (G.C.)

**Keywords:** plastics wastes, aquatic environments, antibiotic residues, plastisphere, antibiotic resistance genes, fish, seafood

## Abstract

This comprehensive review compiles current knowledge about the connection between plastic waste and the selection and transmission of antibiotic resistance genes (ARGs) in aquatic ecosystems, which can result in ARG contamination of fishery products—a significant source of microplastic (MP) introduction into the food chain. Plastic debris in aquatic environments is covered by a biofilm (the plastisphere) in which antibiotic-resistant bacteria (ARB) are selected and horizontal gene transfer (HGT) of ARGs is facilitated. The types of plastic waste considered in this study for their role in ARG enrichment are mainly microplastics (MPs), and also nanoplastics (NPs) and macroplastics. Studies regarding freshwaters, seawaters, aquaculture farms, and ARG accumulation favored by MPs in aquatic animals were considered. Most studies focused on the identification of the microbiota and its correlation with ARGs in plastic biofilms, while a few evaluated the effect of MPs on ARG selection in aquatic animals. A higher abundance of ARGs in the plastisphere than in the surrounding water or natural solid substrates such as sand, rocks, and wood was repeatedly reported. Studies regarding aquatic animals showed that MPs alone, or in association with antibiotics, favored the increase in ARGs in exposed organisms, with the risk of their introduction into the food chain. Therefore, reducing plastic pollution in water bodies and aquaculture waters could mitigate the ARG threat. Further investigations focused on ARG selection in aquatic animals should be conducted to better assess health risks and increase awareness of this ARG transmission route, enabling the adoption of appropriate countermeasures.

## 1. Introduction

According to the first global and regional estimate of antimicrobial resistance (AMR) mortality, from 1990 to 2021, deaths directly attributable to drug-resistant infections or associated with drug-resistant infections increased by more than 25,000 annually, with methicillin-resistant *Staphylococcus aureus* (MRSA), multidrug-resistant *Mycobacterium tuberculosis*, carbapenem-resistant *Klebsiella pneumoniae*, and carbapenem-resistant *Acinetobacter baumannii* as the bacteria most often implicated. It was also estimated that by 2050, 39.1 million deaths attributable to AMR and 169 million deaths associated with AMR may occur among people aged 70 years and older [1].

Plastic waste is a widespread environmental pollutant originating from multiple sources, including agriculture and food production, with almost 50 million tonnes of plastic used for cultivation, animal husbandry, fisheries, forestry, and food packaging in 2019. This amount is projected to rise to 9.5 million tonnes globally by 2030 [2]. The main sources of plastic waste in aquatic ecosystems are wastewater treatment plants (WWTPs), irrigation with wastewater, sludge fertilization, and landfill leachates reaching surface and groundwater [3].

According to fragment size, plastic materials are classified as macroplastics (>2.5 cm), mesoplastics (0.5–2.5 cm), microplastics or MPs (<5 mm), and nanoplastics or NPs (<100 nm) [3,4]. MPs of polymers such as polyethylene (PE), polypropylene (PP), polystyrene (PS), polyethylene terephthalate (PET), and polyvinyl chloride (PVC) originate either from the direct emission of microbeads into the environment or from the chemical or mechanical fragmentation of larger plastic debris. These constitute a major component of marine litter, defined as any solid waste of human origin discarded in the sea or reaching the sea by dispersion from other environments [5,6].

The presence of MPs, in the form of textile microfibres or other shapes, in the gastrointestinal tract of marine fish and seafood species intended for human consumption has been demonstrated in numerous studies. Therefore, these food sources pose a significant risk of MP introduction into the human diet. Small fishes and seafood are often consumed without prior evisceration, and it has also been shown that evisceration does not prevent the presence of MPs in edible tissues [5,7,8]. MPs have been detected in aquatic organisms such as fish, crustaceans, molluscs, algae, corals, and marine mammals in at least 57 countries and regions, with concentrations found to increase from zooplankton to fish. Benthic crustaceans and molluscs can contain hundreds or even several thousand MP particles, originating from both sediment and water. These are ingested or absorbed through respiration by fish and can even be transferred to eggs. The amount of MPs in fish depends on the level of local pollution, fish size, feeding type, speed of movement, and the depth inhabited by the species. Aquaculture fishes have been found to ingest higher amounts of MPs than wild fishes from the same area, mostly as fibers, since these are not retained by water filtration systems. Additionally, MPs in these systems originate from plastic equipment [9].

The absorption of antibiotics by MPs in aquatic environments began to be investigated after it was observed that sulfonamides, trimethoprim, and fluoroquinolones are stable in water, and that MPs can transfer organic pollutants to the food web of aquatic organisms. It was found that ciprofloxacin, amoxicillin, and tetracycline are adsorbed onto polyamide (PA) through hydrogen bond formation, and that ciprofloxacin, amoxicillin, trimethoprim, sulfadiazine, and tetracycline were absorbed at levels following the order PP > PS > PE > PVC. Adsorption on MPs was more efficient in freshwater than in seawater, as both antibiotics such as ciprofloxacin and amoxicillin and MPs are negatively charged in this environment [10].

Plastic waste dispersed in seawater and freshwater harbors a specific biofilm microbiota known as the “plastisphere”, a term coined after scanning electron microscopy (SEM) revealed a diverse microbial consortium on plastic debris filtered from the North Atlantic Ocean. This biofilm also included pathogens of the genus *Vibrio*, as shown by high-throughput sequencing (HTS) of the small rRNA gene subunit [11]. Biofilm formation occurs through the phases of microorganism adsorption onto the plastic surface, formation of multilayered clusters of bacterial cells, and development of a three-dimensional network [3]. Biomass growth on plastic particles is favored by the so-called “ecocorona”, which results from the accumulation of organic and inorganic materials on their surface, facilitating colonization by microorganisms [12]. Plastic biofilms can contain 100 to 5000 times higher loads of antibiotic-resistant bacteria (ARB) compared to the surrounding water [3,13], as antibiotic adsorption creates selective pressure favoring the predominance of ARG bacterial carriers [14]. Mobile genetic elements (MGEs) involved in ARG horizontal gene transfer (HGT) and detected in the plastisphere include the class 1 integron, gene cassette capture elements involved in ARG spread and encoding the integrase *intI*1, as well as plasmids pBB55, pGS05, and the conjugative plasmid RP4, which encodes resistance to kanamycin, tetracycline, and ampicillin [4,14].

Biofilm development is promoted by plastic degradation resulting from exposure to UV rays, aging, oxidation, and thermal stresses, which increase surface roughness and facilitate bacterial colonization. Size, shape, and color have been shown to influence biofilm development on MPs. Biofilms alter the morphology, hydrophobicity, surface area, and functional groups of MPs, affecting their capacity to adsorb chemical pollutants [14]. For example, photoaging in water led to an increase in high-energy binding sites and greater adsorption of tetracycline on PVC MPs. In biofilms formed in a suspension of activated sludge, the abundance of microorganisms and the abundance of tetracycline ARGs (*tet*A, *tet*B, *tet*C, *tet*D, *tet*E, *tet*G, *tet*K), as determined by qPCR, were higher on aged MPs [15].

In one of the first studies, the MP-associated microbiota was found to vary in composition according to nutrient availability, salinity, and proximity to the coast, and also differed between PE and PS. Bacterial families enriched on MPs at all coastal stations were Hypomonadaceae and Erythrobacteraceae. Hypomonadaceae produce strongly adhering polysaccharides and possess cell appendages called prosthecae that enable the uptake of nutrients from a wide area. Therefore, these bacteria are the first to adhere even to smooth MPs. Erythrobacteraceae are abundant on MPs because they degrade the polycyclic aromatic hydrocarbons (PAHs) adsorbed on plastic. In addition, the potentially pathogenic genus *Tenacibaculum* from the family Flavobacteriaceae was detected on both PE and PS. The genus *Sphingopyxis* from the family Sphingomonadaceae was abundant on MPs in a WWTP and was found to harbor a Class I integron encoding ARGs leading to the hypothesis that MPs represent a hotspot for ARG HGT [16]. Indeed, it has been shown that the high bacterial density in MP biofilms facilitates ARG transfer via the three main routes: natural transformation, conjugation, and transduction [14]. Therefore, the detrimental health effects of MP ingestion by humans and animals—such as oxidative stress, cytotoxicity, chronic inflammation, neurological and immune disorders, and toxicity caused by the release of chemicals including bisphenol A (BPA), polychlorinated biphenyls (PCBs), polycyclic aromatic hydrocarbons (PAHs), and organochlorine pesticides [5]—can be further exacerbated by their potential to disseminate AMR.

The purpose of this comprehensive review was to summarize the updated evidence on the involvement of plastic debris in the selection, increase, and HGT of ARGs and multidrug resistance genes (MDRGs) in aquatic environments, as well as their transmission to aquatic organisms, including species used as food. Scientific articles on these topics were retrieved from Google Scholar (https://scholar.google.com/schhp?hl=it, accessed on 24 September 2025) and EMBASE (https://www-embase-com.bibliosan.idm.oclc.org/, accessed on 24 September 2025) using two search strings: “antibiotic resistance AND microplastics AND water” (string 1), and “antibiotic resistance AND microplastics AND fish” or replacing “fish” with the word “mollusc”, “mollusk”, “crustacean”, “shellfish”, “seafood” or “sea food” (string 2). The searches were sorted by relevance in Google Scholar without time restriction, and article screening continued until two consecutive pages did not yield further relevant publications. The scientific articles considered in this study were selected from the first 770 retrieved from Google Scholar and the 283 articles retrieved from Embase using search string 1. With search string 2 and all its variants, the first 50 articles retrieved in Google Scholar were considered, while in Embase, 12 articles were retrieved with the word “fish”, 9 with “sea food”, 4 with “seafood”, 4 with “crustacean”, and 3 with “mollusc”. The first selection was based on the title, the second on abstract content, and the third on the full text content. The articles ultimately considered were those documenting the role of plastic waste in the selection and spread of ARGs and MDRGs in aquatic species habitats. Special attention was given to the correlations established between the microbiota in the plastisphere and ARGs, as well as to the factors found to influence their composition. Accordingly, articles elucidating the physiological mechanisms that favor ARG HGT in the plastisphere were also discussed. Both non-biodegradable plastics (NBPs) and biodegradable plastics (BDPs) were taken into account, and NPs and macroplastics were also considered as substrates involved in ARG selection and transmission. Throughout the text, the indicated ARG functions are those defined by the Comprehensive Antibiotic Resistance Database (CARD, https://card.mcmaster.ca/home, accessed on 20 July 2025), and the nomenclature of the microorganisms is that used in the original source. The currently valid names can be found at https://lpsn.dsmz.de/ (accessed on 30 September 2025). The studies cited in each section are ordered chronologically to highlight the evolution of investigation methods and the progression of research achievements.

## 2. Antibiotic Adsorption on Plastic Polymers

The adsorption of environmental antibiotic residues onto plastic debris promotes their colonization by antibiotic-resistant bacteria (ARB), and studies on antibiotic binding help to explain the dynamics of antibiotic resistance gene (ARG) selection. Adsorption isotherms were initially used for this purpose, employing antibiotic concentrations in the range of 0.2–50 mg/L, which are much higher than those found in the environment, where concentrations are typically in the ng/L–µg/L range. Adsorption isotherms showed that aged plastics have increased adsorption capacity, and that the interactions favoring the binding of substances to plastics are: (i) electrostatic, i.e., dependent on the electrical charges of the polymer and the antibiotic; (ii) hydrophobic, i.e., occurring between non-polar plastics such as PS, PE, PP, and PET and antibiotics, at a rate dependent on the hydrophobicity of the latter; (iii) Van der Waals and π–π interactions for aliphatic polymers such as PVC and PE, and aromatic polymers such as PS, respectively; and (iv) pore-filling, with larger pores in plastic debris adsorbing a higher number of antibiotic molecules. Moreover, antibiotic adsorption increases as the microplastic (MP) particle size decreases, due to the greater surface-to-volume ratio. For this reason, aging of MPs caused by UV radiation, mechanical shearing, and exposure to extreme temperatures increases their adsorption capacity. Parameters such as pH, presence of electrolytes, dissolved organic matter, and competing contaminants influence the rate of antibiotic adsorption on MPs [17].

Recently, machine learning (ML) has been applied to develop generalizable prediction models for the adsorption of various antibiotics onto plastic materials. One such model is based on poly-parameter Linear Free-Energy Relationships (ppLFER) molecular descriptors derived from Quantitative Structure Property Relationship (QSPR) data obtained from scientific publications on individual adsorbents and adsorbates. This model combines the tree-based random forest (RF), eXtreme Gradient Boosting (XGB), and Light Gradient Boosting Machine (LGBM) algorithms. The model considers the following characteristics for the adsorbents: specific surface area (SSA), which determines the number of active sites; carbon percentage (C%), which determines surface homogeneity and influences the affinity for adsorbates depending on hydrogen bonds and Van der Waals forces; the hydrogen-to-carbon (H/C) ratio, which determines the aromaticity of MPs and their interaction with aromatic antibiotics; and the oxygen-to-carbon (O/C) ratio, which indicates the presence of hydroxyl, carboxyl, and carbonyl groups and the tendency to interact with ionic antibiotics. For the antibiotics, the Abraham descriptors, which correlate structure and properties under different conditions, were taken into account. The output parameter was the logarithm of the solute equilibrium partitioning coefficient, “Kd”. The best-fitting model maximized the coefficient of determination (R^2^) and minimized the root mean square error (RMSE). The SHapley Additive exPlanation (SHAP) method identified the hydrophobicity of the antibiotic, the polymer surface polarity, the fraction of ionized species at the test pH, and the molecular volume as the top adsorption predictors [18].

The molecular dynamics (MD) approach to studying antibiotic–MP interactions offers an alternative to experimental methods for elucidating the adsorption dynamics of antibiotics in MPs and determining how these processes contribute to their transport and persistence, which is important for risk assessment and remediation monitoring. Amoxicillin, tetracycline, and ciprofloxacin were used as model molecules; 50 repeat units of PP and PS served as model MPs; and a model water with added NaCl ions represented the natural environment in computational simulations of all possible molecular interactions based on the molecular structures. Oxidative aging was simulated by introducing hydroxyl (-OH) and carbonyl (-C = O) groups. This method predicted the enhancement of adsorption by aged MPs due to increased roughness and the introduction of polar groups. It was shown that PS, particularly when aged, adsorbs antibiotics more efficiently than PP because of its higher hydrophobicity and π–π interactions with aromatic antibiotics. However, PP also binds antibiotics efficiently after aging due to increased polarity. This model indicated that MPs prolong the persistence of antibiotics in aquatic environments. Root mean square deviation (RMSD) analysis demonstrated the structural stability of molecules during the adsorption process [19].

In addition to antibiotics, plastic polymers adsorb extracellular DNA, particularly under conditions of high ionic strength that reduce electrostatic repulsion. Among PE, PET, and PS MPs, PET showed the highest adsorption capacity for linear DNA, possibly facilitated by the ester groups, and adsorption occurred within a short time (30 min) [20].

## 3. Demonstrations of ARG HGT in the Plastisphere

Studies demonstrating ARG HGT by natural transformation or conjugation in MP biofilms or in the presence of NPs were conducted under controlled conditions using model bacteria. It was shown that *E. coli* DH5α exposed to PS NPs was transformed with increased frequency by the plasmid pUC19 encoding ampicillin resistance, produced dose-dependent amounts of reactive oxygen species (ROS) as determined with the fluorescent dye H2DCF-DA, and exhibited increased catalase (Cat), superoxide dismutase (Sod), and glutathione peroxidase (GSH-Px) activities. Addition of the ROS scavenger thiourea reversed ROS formation and decreased transformation frequency, thus confirming that oxidative stress favored transformation. Moreover, NPs induced increased expression of the secretion system genes *sec*A and *sec*B, the arginine translocase gene *tat*A, and the gene *hof*B involved in the synthesis of the type II/IV conjugative pilus [21].

A strain of *Bacillus subtilis* and a strain of *Acinetobacter baylyi*, both naturally competent bacteria, were allowed to form biofilms in a mineral solution on PE, PP, and PS MPs in the presence of plasmid pUC19 and the non-conjugative plasmid pRK415, which encodes tetracycline resistance and the green fluorescent protein gene *gfp* for the identification of tranformants by fluorescence microscopy. The frequency of transformation for both plasmids was higher in the MP biofilms than in sand, used as a control natural substrate (NS), and in water. Its extent followed the order PP > PE > PS, with PP showing the highest bacterial density. The transformation frequency was higher in MPs of 300 µM size compared to those of 3 mm size and in MPs aged by exposure to UV light. Transcriptome analysis showed that on PS and PP biofilms, the flagellum movement genes *motA*, *glgA*, and *bss*R, as well as the quorum sensing (QS) gene *lsr*K, were upregulated in *B. subtilis*. The genes involved in polysaccharide synthesis, *pga*A and *pga*B, were upregulated, and biofilm formation genes *com*A and *pli*X were slightly upregulated in both bacteria in smaller PP particles, with higher expression levels on aged particles. In a multi-species microcosm in river water, the transformation efficiency of pRK415 was higher in the MP biofilms than in the NS at certain time intervals. The transformants identified by 16S rRNA gene sequencing belonged to the species *B. cereus*, *Paraclostridium* spp., and *Enterococcus faecalis.* The latter species was detected only in MPs [22].

The role of QS in ARG transfer by transformation in MP biofilms was demonstrated in *B. subtilis* CGMCC 1.286, which produces acyl-homoserine lactone (AHL) QS signal molecules, including C8-HSL, 3-oxo-C10-HSL, and C12-HSL. Their exogenous addition at concentrations ranging from 0 to 10,000 ng/L resulted in a dose-dependent increase in extracellular polysaccharide (EPS) synthesis and transformation frequency of pRK415 in biofilms formed on PS, PE, and PP MPs in mineral solution. This was of the order of 10^−4^ transformants per recipient, and higher on MPs than on NS, as the bacterial density in the MP biofilms was about one hundred times greater. The QS inhibitors 2(5 H)-furanone, furaneol, coumarin, and benzpyrole caused a decrease in EPS production and transformation efficiency. AHL presence led to upregulation of the competence genes *com*X and *com*A, the environmental DNA uptake gene *rec*O, and the EPS production genes *tasA*, *epsG*, and *tapA*. The latter were downregulated upon exposure to QS inhibitors [23].

A frequency of plasmid transfer by conjugation three orders of magnitude higher than in water was observed in PS MPs in a microcosm containing water from Lake Stechlin (Germany), in which the *E. coli* MG1655 donor, chromosomally tagged with the red-fluorescence expressing cassette *lac*Iq-Lpp-mCherry-km^R^ and harboring the self-transmissible trimethoprim resistance plasmid pKJK5 tagged with *gfp*, and a *Pseudomonas* spp. recipient isolated from the lake were incubated. The *lac*I repressed *gfp* in the plasmid donor, so this was expressed only in the transconjugants, which were identified by green fluorescence using flow cytometry and confocal microscopy. Bacteria detached by two methods from PS MPs deployed for four weeks in the lake received plasmid pKJK5 in mating experiments with the *E. coli* donor, with a transfer frequency one order of magnitude higher than that of free-living bacteria isolated from the lake water. Metataxonomy showed that the microbiota consisted mainly of Gammaproteobacteria, Actinobacteria, and Betaproteobacteria, but with a different genus distribution between MPs and water, and that *Arthrobacter* was the genus transformed with the highest frequency [24].

The same donor/recipient system was used to demonstrate that tire wear particles (TWP) of 10–100 µm size, composed of styrene–butadiene rubber, supported conjugation, particularly at 1:10 and 1:100 donor/recipient ratios. The bacteria colonized the TWPs. The transfer frequency was 27 times higher than in suspension and in PS MPs. HGT was also enhanced by TWPs when a lake water bacterial community was tested as the recipient [25].

SEM showed that the red fluorescent donor *E. coli* MG1655 and the recipient *E. coli* ATCC 25922, or recipient sludge bacteria, incubated in LB broth at 37 °C in the presence of 0 to 500 mg/L on a polydimethylsiloxane (PDMS) membrane chip, formed a biofilm on PS particles with a diameter greater than 2 µm, while particles of 0.2 µm aggregated on the bacterial cell surface. At MP concentrations below 200 mg/L, the 0.2 µm particles promoted conjugation, and at higher MP concentrations, the larger particles also promoted conjugation. Plasmid transfer to sludge bacteria was highest on particles of 15 µm/20 µm at a concentration of 500 mg/L, which induced upregulation of the mating genes *trb*Bp and *tra*F, as well as the DNA transfer and replication genes *trf*Ap and *tra*J in *E. coli*. The 0.2 µm particles increased cellular ROS and membrane permeability. Contact between transconjugants and MPs was demonstrated by combining fluorescence observations with bright field images and considering the theoretical geometric contact probability [26].

The *E. coli* HB101 donor strain harboring plasmid RP4 and the recipient strain *E*. *coli* NK5449, resistant to rifampicin, showed no significant variation in the expression of genes related to oxidative stress in biofilms formed on 4 mm PP, PS, PVC, and PE particles in simulated estuarine water with controlled nutrient concentration. However, there was a 4- to 11-fold increase in the expression of the outer membrane protein genes *omp*A, a pathogenicity factor involved in adhesion and invasion, as well as *omp*C, and *omp*F, which form osmo-protection and antibiotic resistance channels, and a 1- to 5-fold increase in the expression of *tra*G, involved in the formation of the mating apparatus, conjugative pilus, and DNA transfer, occurring to a greater extent in PE biofilms [27].

A theoretical investigation using *E. coli* as the donor and *Pseudomonas aeruginosa* as the recipient indicated that the average binding energy with the predicted active codons of the *E. coli* plasmid harboring multiple ARGs for enzymes involved in HGT—namely, the relaxase, the type IV secretion system (T4SS) coupling protein (T4CP) and its component VirB5, and the DNA ligase of *P. aeruginosa*—increased by 1.12 to 2.02 times in the presence of PP, PVC, PET, PE, PS, and polycarbonate (PC). This finding is consistent with previous evidence that VirB5 is upregulated in the presence of PE and PVC MPs. The multiple criteria decision making (MCDM) method VIKOR identified PP as the material posing the highest risk of HGT. The GROningen MAchine for Chemical Simulations (Gromacs, https://www.gromacs.org/, accessed on 12 April 2025) biomolecule interaction prediction software indicated that PP stress increased the number of hydrogen bonds between the active codons and the relaxase by 3 to 4 times, and those with VirB5 by 8 to 10 times, with a similar increase observed upon exposure to PC [28].

Conjugative transfer occurred between the donor strain *P. putida* KT2440::*lac*Iq-dsRed, harboring the conjugative plasmid *gfp*-RP4, and the recipient strain *E. coli* NK5449 in LB medium in the presence of PVC and PS MPs. Laser scanning confocal microscopy (LSCM) enables observation of green-fluorescing transconjugants appearing earlier and at a higher ratio on donors during biofilm growth and maturation on PVC compared to PS [29]. The conjugal transfer on PVC was most likely favored by oxidative stress, as ROS were more abundant in PVC and in the smaller particles and, according to flow cytometry analysis, induced a dose-dependent membrane permeabilization in the recipient bacteria. The enrichment of DNA replication and repair pathways and QS signal transduction systems in smaller particles was determined by functional prediction with Tax4Fun [30]. The PVC leachate obtained by rinsing MP particles promoted HGT while, in the case of PS, the solid particles rather than the leachate favored HGT. A Bliss independence model analysis [31] highlighted that the effect of PVC particles and leachate on HGT frequency was synergistic [29].

Polylactic acid (PLA) is the most widespread BDP, and its global production is steadily increasing. It has a degradation time of decades and a high tendency to fragment, resulting in debris accumulation in water and the release of large amounts of its highly toxic plasticizer dibutyl phthalate (DBP), which constitutes 10% to 70% of its weight. After 12 h of incubation, PLA MPs and DBP increased the conjugation frequency between *E. coli* Stbl4, a donor of a mobilizable plasmids encoding chloramphenicol and tetracycline resistance, and the recipient *E. coli* JM109, by about two-fold at concentrations of 100 mg/L and 0.1–100 µg/L, respectively. The combination of PLA MPs at 1 mg/L and DBP at 1 µg/L slightly increased the conjugation frequency [32].

Transformation with the pAC plasmid encoding chloramphenicol resistance increased by 28% in the presence of 10 to 100 mg/L PLA MPs and by 1.2- to 1.5-fold in the presence of DBP, reaching the highest level with the combination of PLA MPs at 1 mg/L and DBP at 1 µg/L. ROS formation increased in bacteria exposed to 1 to 100 µg/L DBP and was further enhanced when PLA MPs were also present. Staining with 2′,7′-dichlorofluorescein diacetate (DCFH-DA) showed maximum membrane permeability in the presence of 100 mg/L PLA, 100 µg/L DBP, and the combination of PLA MPs at 1 mg/L and DBP at 1 µg/L. An increase in total antioxidant capacity (TAOC), activation of the SOS response indicated by upregulation of *umu*C and *lex*A, a threefold increase in EPS levels and adhesion, upregulation of the membrane permeability genes *omp*A and *omp*C, and the mating genes *trf*Ap and *trb*Bp were observed in donor cells. In addition, DBP induced upregulation of the DNA translocation, recombination, and replication genes *bhs*A, *yba*V, *nfs*B, and *rec*F [32].

Among the BDPs, polyhydroxybutyrate (PHB) and PLA and the NBP PS NPs, the latter produced fewer NPs after exposure to light in water for 60 days. No toxicity of the NPs was observed in *E. coli* DH5α harboring the plasmid RP4 and *E. coli* HB101, used as donor and recipient, respectively, to analyze plasmid conjugation frequency. Exposure to PS NPs did not alter the plasmid transfer frequency, possibly due to their low concentration, while for the BDPs an increase in transfer frequency was observed, mainly for PLA, as PHB NPs were unstable in solution and aggregated. The increase in transfer frequency was up to 28-fold for PLA NPs and up to about 13-fold for PHB NPs. An increase of about two-fold in membrane permeability, based on LDH release, was observed upon exposure of the two strains to PLA NPs, and the expression of proteins *omp*A and *omp*B reached a maximum, increasing six- to sevenfold. Both BDP NPs induced upregulation of the global regulators *kor*A, *kor*B, and *trb*A, resulting in increased expression levels of *trf*Ap and *trb*Bp [33].

PS spherical particles of approximately 1 µm in size at a concentration of 1 mg/L, as may be found in the aquatic environment, increased the conjugative transfer frequency of plasmid RP4 from *E. coli* DH5α to *E. coli* HB101. However, when combined with 1 mg/L of the neurotoxic and endocrine-disrupting plasticizer di(2-ethylhexyl) phthalate (DEHP), the transfer frequency decreased, possibly due to the increased hydrophobicity of the PS/DEHP combination [34].

The acquisition of phenotypic AMR occurred in *E. coli* ATCC 700926 allowed to attach for 24 h to 10 µm diameter PS spheres at concentrations between 10,000 and 15,000/µL in LB broth and exposed to sublethal concentrations of ampicillin, ciprofloxacin, doxycycline, and streptomycin. The MIC for ciprofloxacin, doxycycline, and streptomycin increased by 100-fold after 10 days for cells detached from the 500 µm PS MPs. Moreover, a 75- to 171-fold increase in multidrug resistance (MDR) was observed. Ten days after cessation of antibiotic exposure, bacteria exposed also to MPs or to MPs alone retained or increased the level of AMR to a greater extent than those exposed only to antibiotics, particularly those exposed to doxycycline and MPs. The minimum inhibitory concentration (MIC) for ciprofloxacin increased more than 3-fold after exposure to sublethal concentrations of this antibiotic and larger PS particles, and more than 2-fold for smaller particles, reaching values higher than the clinical *E. coli* breakpoint in the presence of MPs only. Cells detached from MPs showed an increased capacity to form biofilms in Petri dishes, impaired motility, and a greater increase in ciprofloxacin MIC compared to planktonic cells [35].

Finally, PS MPs were found to increase the mutation rate, calculated as the ratio of bacteria acquiring resistance to rifampicin to the total number of bacteria, in *E. coli* K12. This effect was greatest with particles functionalized with NH_2_ to acquire a positive charge and with a 0.1 µm diameter, combined with norfloxacin at concentrations possibly found in estuary water. These combinations also promoted the highest conjugation frequency of plasmid RP4 between *E. coli* strains, as well as an increase in ROS and membrane permeability [36].

## 4. Occurrence of ARGs and Bacterial Hosts on Plastic Fragments in Water Bodies

The experimental procedures used in studies on plastisphere formation, ARG selection, and HGT in aquatic environments are not described in detail in this review, as they are covered in a dedicated review that discusses their advantages, disadvantages, and developments. These procedures involved analyzing biofilms formed either after in situ deployment of MPs in cages in water bodies or after suspending MPs in laboratory-scale microcosms containing water from natural sites or suspensions that mimic their composition. Alternatively, direct microbiological and biomolecular examination of plastic debris isolated from aquatic environments and collected by pumping and filtration, together with identification of the plastic polymers and analysis of plastic degradation by Fourier transform infrared (FTIR) and Raman spectroscopy, was also used. SEM was employed to observe plastic degradation, biofilm growth, and EPS formation. Studies on microbial community composition in the plastisphere were conducted either by isolating and molecularly identifying isolates using 16S rRNA gene sequencing, or by extracting whole DNA from plastic samples followed by HTS of 16S rRNA gene regions (e.g., V4, V6, V3/V4, V4/V5, and V4/V6) for prokaryotes, or the 18S rRNA gene, ribosomal internal transcribed spacers (ITS), and the 26S rRNA gene for eukaryotes [37]. This type of analysis is referred to as “metataxonomic” in this review.

ARGs and MDRGs were detected by quantitative Polymerase Chain Reaction (qPCR), high-throughput qPCR (HT-qPCR), and/or metagenome sequencing, which simultaneously identified the microbial composition of the plastisphere and the enriched cell functions. Bacterial abundance was generally determined as the 16S rRNA gene copy number, and the relative abundance of ARGs was expressed as the copy number of ARGs per 16S rRNA gene copy number. These parameters were compared with those in water or natural solid substrates such as sand, gravel, or wood. Therefore, the definition of “relative abundance” in this review is used as described above, unless otherwise specified. Network correlation analysis was widely used to identify the most probable bacterial hosts of ARGs, ARG associations, and ARG variation with environmental parameters [37].

A systematic review of 69 plastisphere metagenome datasets from the literature established the correlation between bacterial community abundance and ARGs in MPs from aquatic environments using machine learning algorithms: multiple linear regression (MLR), multilayer perceptron (MLP), gradient boosting decision tree (GBDT), and random forest (RF). Among plastic polymers, PVC appeared most frequently, followed by PE, polyhydroxyalkanoate (PHA), and fibers, which were the most common microplastic shape. The bacterial groups associated with MPs, in order of abundance, were Pseudomonadota, Actinomycetota, and Bacteroidetes, with genus diversity ranging from about nine hundred on PLA to more than two thousand on phenol formaldehyde (PF). ARG reads per million decreased from more than one thousand to less than one hundred in the following order: PHA > PLA > PE > PS > PFP > PP > PF > PA/PET. More than 85% of the ARGs were MDRGs, followed by ARGs for sulfonamides, tetracyclines, and aminoglycosides in order of abundance. ARGs for macrolides, penicillin, and rifampicin were also frequently reported. The enriched ARG types varied with the MP polymer, and the BDPs presented the highest risk of ARG spread. All four algorithms enabled the prediction of the presence of ARGs based on the occurrence of bacterial genera and the MP type, particularly the MLR. More accurate prediction of ecological risks and the efficiency of ARG removal treatments will be achieved by considering the water environment (i.e., freshwater or seawater), MP aging, antibiotic occurrence, and larger metagenomic datasets [38].

Salinity influences microbial diversity, abundance, and ARG type in the plastisphere, according to a study in which the biodegradable polybutylene adipate-co-terephthalate (PBAT) and PET MPs were submerged in freshwater and seawater in separate tanks for two weeks. Subsequently, those in freshwater were transferred to seawater and vice versa for an additional four weeks. The quinolone resistance gene *qnr*S and the sulfonamide resistance gene *sul*2 were dominant in the plastisphere in both water types but the tetracycline resistance gene *tet*A and *qnr*A were detected in seawater only after the transfer, being most probably released from the plastisphere. Conversely, the macrolide resistance gene *mef*A became bound to MPs after the transfer, most probably because it was already present in seawater. Rhizobiaceae, *Romboutsia*, and *Clostridium*_*sensu*_*stricto*_13 decreased after MP transfer to seawater, possibly inhibited by salinity. Network analysis showed that fewer bacterial genera in the PBAT plastisphere were positively correlated with ARGs in seawater compared to freshwater, while the opposite was observed for PET, particularly for *tet*A and the chloramphenicol resistance gene *cml*A1. When entering freshwater PBAT became colonized by genera positively correlated with *qnr*S, including *Afipia* and the Rhizobiaceae. The genus *Bacillus* was positively correlated with *sul*2 and the beta-lactamase *bla*Q, while genera *Gemmobacter*, *Conexibacter*, and *Lamia*, were positively correlated with *tet*A, *tet*C, *tet*X, and the erythromycin esterase *ere*B. When MPs entered seawater, the MGEs *tnp*A04 and *tet*A, *qnr*S, and *bla*Q became strongly associated with the genus *Labrenzia* and the family Vicinamibacteraceae while *tnp*A05 co-occurred with *qnr*B in *Coxiella* spp., the Acidobacteriota *Sva0996_marine_group*, and the genera *Croceibacter* and *Tumebacillus*, indicating HGT among different genera. PBAT enriched more ARGs than PET, possibly due to the release of carbon sources and rougher surfaces that favored bacterial adhesion [39].

The study mentioned above indicated that the microbial groups harboring ARGs in MPs in freshwater or seawater differ in composition, so the following sections separately address studies regarding freshwaters, seawaters, and waters with intermediate degrees of salinity.

### 4.1. ARGs in the Plastisphere in Freshwater

This section discusses studies on the occurrence of ARGs in the plastisphere from rivers and lakes conducted in various countries.

One of the earliest investigations reported that MPs collected from two urban rivers in Jiaxing City (China), located in the Yangtze River Delta and composed mainly of PE and PP, harbored lower microbiota diversity than water, with significant enrichment of Gammaproteobacteria, Bacilli, Anaerolineae, Firmicutes, and the genera *Deinococcus*-*Thermus*, and *Nitrospira*, based on metataxonomic analysis. The abundance of ARGs measured by qPCR was lower in MPs than in water, except for certain genes including *tet*M, *tet*S, and *tet*W. Co-occurrence analysis indicated that Proteobacteria acted as ARG hosts in MPs, and the enrichment of integrase genes *intI*1 and *intI*2 in MPs suggested a higher risk of ARG spread in the MP biofilm [40].

Metataxonomic analysis showed that at various time intervals during 30 days of incubation in waters from an urban area, the bacterial family most enriched in the plastisphere compared to water was Comamonadaceae, followed by Planctomycetaceae. The enriched bacterial species were *M. abscessus*, *B. megaterium*, and *P. putida*. The ARGs enriched in MPs based on HT-qPCR were the fluoroquinolone efflux pump *qep*A, *lnu*F, *aad*A7, *bla*_OXY-1_ and *tet*G F. The ARG *erm*E was particularly enriched in MPs, while *flo*R, *cph*A and *aph*4-Ib were found only in MPs at certain time intervals [41].

In the Dutch section of the Rhine River sampled in winter and summer, MP concentrations were higher in summer, with particles of 2–10 µm being more abundant. PA and PVC predominated, but PE, PET, PP, PS, polyurethane (PU), and isoprene were also detected. Metataxonomic analysis showed that the genera *Flavobacterium*, *Simplicispira*, and *Pseudomonas* were positively associated with PET, *Acinetobacter* with PP, *Hydrogenophaga* with PS, *Pseudarcicella* with isoprene, and *Synechococcus* with PU. The ARGs *sul*1 and *erm*B were specifically targeted and showed a prevalence of about 94% and 99%, respectively, in all samples [42].

PHB, low-density PE (LDPE) and high-density PE (HDPE) deployed for 11 days in an Arctic lake on the Svalbard islands (Norway) were colonized by Proteobacteria and the much less abundant phyla Cyanobacteria, Bacteroidetes, Actinobacteria and Verrucomicrobia. *Mycoplana* spp. predominated on both BDPs and NBPs, followed by *Erythromicrobium*, *Zymomonas*, Comamonadaceae and *Rhodobacter*. Additionally, Sphingomonadaceae, *Pseudanabaena*, and *Sphingomonas* were detected in NBPs, while Moraxellaceae and *Polaromonas* were found in BDPs. The ARGs *sul*I and *erm*B were specifically targeted by qPCR and detected in all polymers, with higher abundance than in water and rock biofilm; both were particularly abundant in HDPE [43].

MPs, water, sediment, and wood particles were collected at eleven sites along the Ganjiang River (China), where the most abundant MPs were PE, PP, and polybutadiene (PBD). Metataxonomic analysis showed an enrichment of the genera *Flavobacterium*, *Rhodoferax*, cyanobacteria, an *Unindentified_bacterium*, and *Pseudomonas* spp., including the potentially pathogenic *P*. *protegens* and *P. stutzeri*. ARGs were not enriched in MPs, but their relative abundance and that of the *intI*1 gene were positively correlated, suggesting HGT. The genes *erm*F and *erm*B were found only in MPs and their most probable host was *Streptococcus mitis* [44].

Along the entire Beilun River at the China–Vietnam border, which is characterized by areas with varying degrees of urbanization, the abundance of ARGs in MPs, as defined by HT-qPCR, ranged from 10^3^ to 10^6^ copies/g among 14 sites. These levels were higher than those in water and conferred resistance to beta-lactams, aminoglycosides, multiple drugs, MLS, tetracyclines, sulfonamides, vancomycin, and chloramphenicol, in order of abundance. IS6100, IS26, and *tnp*A-6 were the most frequently detected MGE indicators. ARG and MGE abundance and number increased from rural to urban regions, particularly aminoglycoside, MDR and tetracycline ARGs, and decreased slightly along the estuary. Thirty ARGs, including *tet*A, *tet*B, *sul*1, and *qac*H, predominated and were detected at all sites. In peri-urban and urban areas, some genes such as *aac*(6′)-Ib, *bla*_VEB_, *qnr*B4, *erm*B, *pbr*T, *tet*E, the integron-encoded trimethoprim resistance dihydrofolate reductase *dfr*A1, *van*G, and *mex*B were newly identified. In rural regions, ARGs were more abundant in PE; in peri-urban regions, they were more abundant in PE and PF; and in urban regions, they were more abundant in PP. Aminoglycoside ARGs were enriched in MPs compared to water, and mainly on PE, while the most abundant ARGs in PP were *tet*B and *tet*G. Network correlation analysis showed that IS6100 co-occurred with 20 ARGs in peri-urban and urban areas, and that the genera *Vibrio*, *Flavobacterium*, and *Chryseobacterium* carried at least five ARGs. The latter genus occurs in clinical settings. MDR bacteria such as the genera *Muriicola*, *Robiginitalea*, and *Woeseia*, which are potential hosts of up to 39 ARGs, occurred in urban areas, while bacteria in rural areas showed at most 16 co-occurring ARGs. Socioeconomic factors such as population density, presence of hospitals, and levels of domestic sewage were positively correlated with ARG occurrence. In particular, population density was positively correlated with aminoglycoside and sulfonamide ARGs. These factors were also correlated with MGEs and explained 88% of the variance in ARG occurrence. In rural areas, ARGs were better correlated with bacterial composition compared to peri-urban and urban regions. The projection pursuit regression (PPR) model highlighted that all MP types except pentafluorophenyl acrylate (PFP) showed an increased risk of ARG dissemination with urbanization, following the order PP > PE > PS > PF > PFP [45].

In PE and PS MPs deployed for 30 days at two sites in the Houxi River watershed (China), the dominant taxa in the plastisphere were Proteobacteria, Bacteroidetes, and Cyanobacteria. Oxyphotobacteria were enriched on MPs in the natural reserve area, while α-Proteobacteria were enriched in the bay area. The number of ARGs in the plastisphere was significantly lower than in water, and even lower in the natural reserve area, but a positive correlation was found between the relative abundance of ARGs and MGEs. The bay MP samples carried a higher abundance of potential pathogens than water, among which *K. pneumoniae* and *Enterobacter cloacae* were associated with at least two ARGs [46].

In the Huangpu River (China), sampled at ten sites, PET fibers were the main MP component, followed by PA, polymethyl methacrylate (PMMA), PE, and PP. Metagenome sequencing showed that the relative abundance of ARGs for tetracycline and chloramphenicol was higher, and ARGs for rifamycin and vancomycin were selectively enriched in MPs. ARG profiles were more diverse in MPs than in water, with a total of 313 subtypes. Subtypes of the lincosamide resistance gene *lnu*A, an unspecified tetracycline resistance gene, the integron-encoded ribosyltransferase *arr* conferring rifampicin resistance, the efflux pump component *mex*I, *bla*_LRA-12_, and the efflux pump gene *ros*A were selectively enriched. MGEs associated with MPs were less diverse than those in water, but the plasmid genes *Rep*13, *Rep*21, *Rep*7, and *tnp*AB, linked to 13 subtypes of ARGs, as well as ISRj1, were more abundant. Moreover, *rep*21 and *lnu*A, *Rep*7 and the tetracycline resistance gene showed strong positive correlations, indicating HGT potential for these ARGs. Correlation network analysis showed that the Proteobacteria genus *Afipia* was highly correlated with 28 ARGs, including the aminoglycoside acetyltransferase *aac*(2′)-*I*, *arr*, the chloramphenicol acetyltransferase *cat*, *mex*I, *bla*_TEM-1_, and *tet*V [47].

PE, PVC and PET MPs were incubated in bottles containing filtered river water for one month in the presence of antibiotic-resistant bacteria (ARB) carrying the ARGs *tet*A, *tet*C, *tet*O, *sul*1, and *intI*1, or in the presence of extracellular ARGs *tet*A and *bla*_TEM_ in plasmids. The ARB studied had been selected by incubating the river water samples with 0.1 mg/L tetracycline for five days and isolating the bacteria grown on LB medium supplemented with antibiotics. After five days, all the intracellular ARGs were detected on the MPs, with higher relative abundance in PET and PVC, and with *sul*1, *tet*A, and *intI*1 at three orders of magnitude higher than *tet*C and *tet*O. Extracellular *tet*A decreased on the MPs in the first 15 days and increased later, while *bla*_TEM_, already at initial levels of 9 Log copy number/g, further increased. The bacterial genera identified on the MPs by metataxonomy and positively correlated with the intracellular *tet*A, *tet*C, and *sul*1 were *Pseudomonas*, *Solobacterium*, *Achromobacter*, *Aeromonas*, *Beggiatoa*, *Propionivibrio*, and *Paludibacter*, with the latter three genera also positively correlated with *tet*O, while *intI*1 was positively correlated with *Tolumonas*, *Pseudorhodobacter*, and *Rhodoferax* [48].

PLA and PVC MP biofilms incubated for 14 days in bioreactors containing water from the Haihe River (China) were analyzed by metataxonomy, shotgun metagenomic and metatranscriptomics. The PLA biofilm was the most diverse, with enrichment of Planctomycetes, Spirochaetes, Gemmatimonadetes, *Deinococcus-Thermus*, Tenericutes, Fibrobacteres, and Cyanobacteria. In the PVC biofilm *Deinococcus-Thermus*, Tenericutes, Epsilonbacteraeota, Actinobacteria, Cyanobacteria, and Bacteroidetes were enriched. Metagenomic analysis identified the macrolide resistance gene *mac*B and MDRGs, particularly *ceo*B, which is part of an efflux system conferring resistance to chloramphenicol, trimethoprim, and ciprofloxacin, as highly abundant in the plastisphere. The diversity of ARGs was highest on PLA, with 173 ARGs detected, of which 75 encoded resistance to 18 antibiotics and were predominantly MDRGs. These were transcribed and showed higher expression level in MP biofilms. Twenty-nine ARGs and 13 ARG transcripts were detected exclusively in the plastisphere. Alignment with the plasmid protein database indicated that some ARGs, expressed to a higher extent in the plastisphere than in water, were plasmid-encoded and thus had HGT potential. Correlation networks showed that ARGs were distributed mainly in the phylum Proteobacteria, followed by Firmicutes, Bacteroidetes, Desulfobacteraeota, Actinobacteria, and Desulfuromonadaeota. Among the identified bacterial hosts, *E. cloacae*, found only in PLA, expressed the *tet*G gene [49].

PET and PBAT incubated in water samples collected from West Lake, a canal, and the Qiantang River (China) for two weeks showed an increased relative abundance of the ARGs initially present in the water, with a more pronounced increase in PBAT, particularly for the *tet* genes. When the MPs were transferred to another water sample, some ARGs such as *tet*C, *tet*G, *sul*1 and *sul*2 were released into the receiving water. Conversely, *cml*A1, which was present in the water, increased in the PBAT MPs and was no longer detected in the water. Positive correlations of ARGs with *intI*1, particularly in PET, and with *tnp*A05 were observed [50].

The pathogenic bacterium *Morganella morganii*, which can cause severe infections in various organs, was isolated from Artificial Plastic Substrates (APSs) submerged in Lake Bracciano (Italy) for one month. The isolates carried *intI*1 and the ARGs *tet*C, *sul*1, *sul*3, the chloramphenicol resistance genes *cml*A1 and *cmx*A, and the extended-spectrum beta-lactamases (ESBL) *bla*_CTX-M-01_ and *bla*_CTX-M-02_ [51].

PP and PET MPs, after 30 days of incubation in microcosm experiments with river water, showed a more abundant biomass compared with gravel rock, reaching numbers on the order of 10^5^ CFU/g. The gene *tet*B was present in PP, as were the ARB genera *Pedobacter* and *Pseudomonas* [52].

The biofilms on PVC, PE, polycaprolactone (PCL), and PLA MPs deployed at ten sites with varying levels of urbanization in the Houxi River (China) and analyzed by metagenomic sequencing showed an abundance of MDRGs, as well as ARGs for macrolide–lincosamide–streptogramin (MLS), sulfonamide and polymyxin, and MGEs increasing with bacterial population density. The most prevalent ARGs in urbanized sites were the bacitracin resistance gene *bac*A, the polymyxin B resistance gene *ugd* (*pmr*E), *sul*1, *sul*2, the multidrug resistance transporter *msb*A and the efflux pump component *acr*B. The most prevalent MGEs were *tnp*A, *tnp*A1, *tnp*A2, *tnp*A3, *tnp*A5 and *intI*1. Aminoglycoside resistance genes increased significantly on PCL and PVC, while quinolone resistance genes increased in PLA and PE. The abundance of MGEs in MPs followed the order PVC < PCL < PE < PLA. Forty-eight metagenome-assembled genomes (MAGs) assigned to Burkholderiales, Pseudomonadales, Enterobacterales, and the genera *Pseudomonas* and *Aeromonas* presented the highest ARG risk. The human fecal marker crAssphage was positively correlated with ARGs and *intI*1, indicating that human fecal pollution caused the increase in ARGs [53].

Metataxonomic analysis of the plastisphere of PET, PP, and Mater-Bi (Mater-Bi, Novamont, Italy) submerged in Lake Maggiore (Italy) for 43 days showed a distinct composition of the microbiota and a higher abundance of potential pathogens, including the genera *Streptococcus*, *Rickettsia*, *Shewanella*, and *Sphingomonas*, compared to water. Microbial diversity was greater in plastic materials than in cellulose, but lower than in wood. ARGs were more abundant on PET and PP, particularly *tet*A, while the *intI*1 gene was more abundant on Mater-Bi and PET [54].

MPs collected from the surface water of Poyang Lake, the largest freshwater lake in China and a National Natural Reserve, consisted mainly of PE and PP. Shotgun metagenomics revealed that these MPs harbored a less diverse microbiota than the surrounding water, which was dominated by Proteobacteria, followed by Actinobacteria. *P. fluorescens* was the most abundant pathogen, and *A. junii*, *A*. *caviae*, *A*. *sobria*, *Brevundimonas diminuta*, *K*. *pneumoniae* and *Rahnella aquatilis* were also detected. The relative abundance of ARGs for bacitracin, beta-lactams, polymyxin, fosfomycin, quinolones, and sulfonamides, as well as their subtype numbers, was significantly higher on MPs, with 184 unique subtypes in PE and 46 in PP. The MDRG *mex*C, *bla*_LRA-13_, and *fos*A7, were detected only on MPs. The genes *bac*A, *bla*_THIN-B_, and the MDRGs *mdt*B and *mex*E-*mex*F-*opr*N were among those most enriched in PP, while *mdt*B was enriched in PE. Network correlation analysis showed that *A. veronii* was associated with the beta-lactamases *bla*_OXA-12_, *cph*A6, *cph*A8, and *cph*A3, and *Arthrobacter* spp. with many ARGs, including the MDRG *mds*B, the novobiocin exporter *nov*A, the rifampin phosphotransferase *rph*A, and *emr*K. Moreover, three novel beta-lactamases were identified with the fARGene software [55]. These exhibited extended spectrum beta-lactamase (ESBL) and carbapenemase activities after cloning and expression in *E*. *coli* BL21 [56].

Metagenome sequence analysis, showed that PBAT, polybutylene succinate (PBS), PHA, PLA, PP, PE, PVC, and PS MPs deployed in situ in four lakes in Wuhan (China) for two months contained a more diverse microbiota and a greater number of opportunistic pathogens than the surrounding water. Proteobacteria predominated on all the polymers; *Firmicutes*, *Nitrospiria*, and *Verrucomicrobia* were enriched on PE, PP, PS, PVC, and PLA, while Proteobacteria and *Candidatus* Parcubacteria were enriched on PHA, PBS, and PBAT. Among pathogens, *Cystobacter fuscus* was the most abundant in all MPs, *Ralstonia solanacearum*, *Burkholderia cenocepacia*, and *B. glumae* were enriched on the PHA, PBS, and PBAT, while *Coxiella burnetii*, *Legionella pneumophila*, and *Xylella fastidiosa* were enriched on the other plastics. A null-model-based stochasticity index indicated that ARG assembly was dominated by stochastic processes in PP and by deterministic processes in the other MPs. MGEs, mainly transposases, were enriched and distributed differently among the plastic polymers. Network analysis revealed a higher number of potential ARG hosts in BDPs, among which *Riemerella anatipestifer*, an avian pathogen, was associated with the MGEs IS621, ISBaba6, ISBf10, *ist*A, and *tnp*A27, as well as the ARGs *nov*A, the macrolide exporter *mac*B, *mds*B, the multidrug efflux pump *sav1866*, the tiamulin efflux pump *tae*A, the polymyxin resistance gene *arn*C, and the quinolone resistance gene *mfd. Vibrio campbellii* was associated with the MGEs ISBaba6 and *tnp*A and with the ARGs *nov*A, *bla*_CRP_, *tae*A, the polymyxin resistance gene *pmr*E and *tet*4 while *V. cholerae* was associated with the amoxicillin resistance gene *pbp1a*, the fosfomycin resistance gene *mur*A, the efflux pump *efr*A, the macrolide efflux pump *mtr*A, *tet*PB, and the MGE *ist*A15 [57].

Twenty-two bacterial isolates obtained from plastic substrates in lake water and identified by 16S rRNA gene sequencing as *Lysinibacillus* spp., *Exiguobacterium acetylicum*, *Klebsiella pneumoniae*, *K. oxytoca* and *K. michiganensis* were submerged in a lake for 30 days in the presence of MPs and adhered to the particles at rates decreasing in the order: PS, styrene acrylonitrile resin (SAN), and, equally, PP and PET. *Klebsiella* spp. was re-isolated only from PS and SAN. After re-isolation, all five bacterial groups shared the gene *bla*_TEM_. *Lysinibacillus* spp. and *Klebsiella* spp. shared *bla*_SHV_, *Lysinibacillus* spp., *E. acetylicum* and *K. michiganensis* shared the MDRG *ade*A, *Lysinibacillus* spp., *E. acetylicum* and *K. pneumoniae* shared *tet*A, *Lysinibacillus* spp., *K. pneumoniae* and *K. oxytoca* shared the efflux pump component *acr*B, *Lysinibacillus* spp., *K. pneumoniae* and *K. michiganensis* shared *sul*1, *Lysinibacillus* spp. and *K. pneumoniae* shared *bla*_CTX-M_, *Lysinibacillus* spp. and *E. acetylicum* shared the beta-lactam resistance gene *mec*A, *Lysinibacillus* spp. and *K. michiganensis* shared *tet*W and the efflux pump *acr*F, *E. acetylicum* and *K. pneumoniae* shared *cmx*A and *K. pneumoniae* and *K. oxytoca* shared *sul*2. In addition, *K. oxytoca* harbored *bla*_CTX_ and the MDRG *acr*R, and *K. michiganensis* harbored *tet*B. The genes *intI*1 and *intI*1V were detected in almost all isolates. These results indicate that HGT events occurred for most of the ARGs detected and involved all the bacteria studied [58].

MPs are distributed at different depths in water according to their density, size, and shape. Therefore, the vertical distribution of ARGs in PET and PLA MPs deployed in the Qinhuai River (China) for 60 days was investigated. At higher depths, biofilm formed more rapidly on PLA, but the biofilm biomass was lower compared to shallower depths due to lower temperature and oxygen availability. However, the relative abundance of ARGs, including *qnr*S, *bla*_NDM-1_, which encodes the superbug New Delhi metallo-beta-lactamase-1 conferring resistance to most beta-lactams and carbapenems, *flo*R, *sul*1, *qnr*A, *tet*G, and the colistin resistance gene *mcr*1, encoding an enzyme that modifies the affinity of lipid A for this antibiotic, increased more markedly in MPs than in water, particularly in PLA. Metataxonomic analysis showed that the genera *Kouleothrix* and *Nitrospira* were enriched in PLA, while *Hydrogenophaga* and *Flavobacterium* were enriched in PET. Correlation analysis linked *Hydrogenophaga*, *Nitrospira*, *Methyloversatilis*, and *Ellin6067* to ARGs in PLA, and *Dinghuibacter*, *Ahniella*, *Dechloromonas*, and *Acinetobacter* to ARGs in PET. The potential for HGT, indicated by the abundance of *intI*1 and *tnp*A05, increased with depth. The *intI*1 gene was predominant and more abundant in PET [59].

A subsequent study conducted in the same river found that proximity to the sediment led to a greater increase in ARG abundance and HGT probability in PET than in PLA MPs after incubation in cages in deep water for 30 days, followed by a further 30 days of incubation at the sediment–water interface. The abundance of ARGs determined by HT-qPCR in deep water was nearly double in PLA compared to PET, while at the sediment–water interface, ARG abundance in PLA decreased by more than 50%, and in PET it increased more than threefold. The concentrations of nitric and ammonia nitrogen, and phosphates were positively correlated with most ARGs, and these increased at the sediment–water interface, indicating that nutrient availability favored ARG increase in PET. Network correlation analysis showed that the genera *Sphingomonas*, *Nitrospira*, *Nitrosomonas*, *Flavobacterium*, and *Kouleothrix* were positively correlated with most ARGs. The total abundance of MGEs in PLA slightly decreased, while it increased by about 1.5-fold in PET, indicating a higher probability of HGT in this polymer. The MGEs *tnp*A1, *tnp*Acp2, *tni*A, IS91, and *intI*1 were positively correlated with ARGs, including the aminoglycoside nucleotidyltransferase *aad*A, *sul*1, *sul*2, *efr*B, and *erm*F [60].

Processes favoring HGT were upregulated in PET and downregulated in PLA. These included QS, as indicated by an increased amount of AHL autoinducers and the upregulation of *arg*AB, *arg*D, *arg*J, *lsr*G, *rpf*C, and *ahl*D; membrane permeability, as indicated by a higher release of the intracellular enzyme lactate-dehydrogenase (LDH) and upregulation of *sec*A, *gsp*D, *yid*C, *sec*Y, *srp*54, *fts*Y, *tol*C, and *gsp*E; ROS production, as indicated by the upregulation of *kat*G, *kat*E, *gpx*, *alk*B, *sox*X, *SOD*1, and *SOD*2; stress response as indicated by the upregulation of DNA integration and repair genes *rec*A, *uvr*A, *uvr*B, *dna*E, and *pol*A; polysaccharide synthesis, as indicated by the upregulation of *lpx*A, *lpx*B, *lpa*D, and *lpx*H; and protein export, as indicated by the upregulation of *tat*A, *tat*B, and *tat*C. Moreover, 11 components of the T4SS system, in particular *vir*B10, which encodes an inner and outer membrane translocase, and *vir*D4, which encodes a receptor for substrate conjugation including ARGs, were upregulated in PET [60].

In MPs sampled at multiple sites from the middle to the final part of the Red River (Vietnam), the abundance of biofilm biomass was not correlated with the plastic polymer type, except for PET and polytridecanolactone (PTDL). Bacteria were isolated on MacConkey agar supplemented with 1 µg/mL cefotaxime from almost all sampling sites, with the highest concentration being of the order of 10^5^ CFU/mL on MPs from industrial sites, and more abundant in freshwater. The presence of chlorophyll-a, originating from primary production, was positively correlated with ARB abundance on MPs. Among 207 isolates, 99% were identified by 16S rRNA gene sequencing as *Aeromonas* spp., including *A. veronii* and *A. caviae* while *E. coli* was isolated from only one site. ESBL genes were detected in 23 isolates by PCR: *bla*_SHV_ from one site, *bla*_TEM_ and *bla*_SHV_ from another site, and these genes plus *bla*_CTX-M_ from a site receiving hospital effluents [61].

PE, PP, PS, PLA, and PHA MPs were incubated for 35 days in Central Lake (China), and EPS formation and biomass increased over four weeks to multiples of 10^7^ cells/g. The order of biomass abundance was PHA > PLA > grit used as NS > PP and PS. Shotgun metagenomic analysis showed that Plantomycetes were present only in MPs, Alphaproteobacteria were enriched in PE, PP, and PS, and Betaproteobacteria were enriched in PHA and PLA. The pathogens *P*. *aeruginosa*, *P. fluorescens*, *S*. *enterica*, *Mycobacteroides abscessus*, *B*. *pseudomallei*, and *K. pneumoniae* showed lower relative abundance on NBPs than in water and BDPs. *Shigella flexneri* and *E. coli* were enriched in PLA, and *P. aeruginosa*, a WHO priority 1 pathogen [62], was enriched in PHA. The relative abundance of ARGs was slightly higher on MPs than in water, in the order PHA > PLA > PS > PE > PP. The relative abundance of MGEs was higher on BDPs with ICE *SXT/R391* enriched in PHA and *intI*1 in both BDPs. Co-occurrence network analysis showed that 74 ARGs and 29 MGEs shared positive correlations. Proteobacteria, mainly *P. fluorescens*, *Xanthomonas campestris* and *X*. *citri*, were the main ARG and MGE hosts and were more abundant in MPs, particularly in BDPs. ARGs classified as rank I risk, based on co-occurrence with MGEs and presence in pathogens [63], namely *bac*A, *mec*A, *qac*A, *tol*C, *dfr*A1, and the macrolide phosphotransferases *mph*A and *mph*B, were more abundant in PHA [64].

In six lakes sampled in the Jiuzhaigou karst plateau (China) in 2023, fibrous MPs predominated and were identified as PE, PET, PP, and PS. Metagenome sequencing revealed a predominance of Proteobacteria, Bacteroidota, and Actinobacteria in the plastisphere, with a higher abundance of Actinobacteria and Chloroflexi in MPs from the sediment. ARGs in the plastisphere were shared with those in water and sediment but exhibited higher alpha-diversity in the MPs. The most abundant ARG was the MDR gene *evg*S [65].

Urban water bodies, specifically an urban park lake, an urban river stretch, and the urban–rural lake in Chengdu (China), were contaminated by PP, PE, PS, and nylon MPs, respectively. At all three sites, metataxonomic analysis showed Proteobacteria as the most abundant phylum, followed by Chloroflexi, Firmicutes, Actinobacteriota, and Cyanobacteriota, as well as the genera *Acinetobacter*, *Rhizobium* and *Exiguobacterium*. *Pseudomonas* spp. and *Acinetobacter* spp. were particularly enriched in the river. Metagenome sequencing revealed more than 100 ARGs at each site, with 72 shared among them. The most abundant ARG at all sites was *bac*A, followed by *sul*1, *sul*2, *ceo*B, the MDR efflux pump component *sme*E, *acr*B, *tet*39 and *tet*C. The aminoglycoside O-nucleotidyltransferases genes *ant*(2″)-Ia and *ant*(3″)-Ia were detected in the river. Based on the presence of MGEs, mainly transposases and recombinases, the risk of HGT was similar in the three environments. Integrases were detected only in the river. Among the bacterial groups carrying MGEs *Acinetobacter* spp. prevailed in the river and *Rhodopseudomonas* spp. in the rural lake. Aminoglycoside, bacitracin, and tetracyclines ARGs were mostly chromosomal, while sulfonamide and rifampicin ARGs were mainly plasmid-encoded. Network correlation analysis showed positive correlations between pathogenic Gammaproteobacteria, Pseudomonadota, Acidobacteriota, and Actinomycetota and *bac*A and *sul*1 in the urban lake; between pathogenic Deltaproteobacteria, Desulfobacterales, and *sul*1, *sul*2, *acr*B, and *ant*(2″)-Ia in the river A; and between pathogenic Gammaproteobacteria and *sul*1, *sul*2, and *sme*E in the rural lake. Moreover, pathogenic Gammaproteobacteria and Pseudomonadota in the urban lake were positively correlated with *ist*A, *ist*B, *tnp*A, and *tnp*A-1; pathogenic Pseudomonadota and Hyphomicrobiales in the river were positively correlated with *ist*A3, *ist*B1, IS91, and *tnp*A; and pathogenic Pseudomonadota in the rural lake were positively correlated with *tni*B, *intI*1, and *qac*Edelta MGEs [66].

Metagenome sequencing of the plastisphere in additive-free BDPs polycaprolactone (PCL) and PHB MPs, and the NBPs PP and PS, and the biofilm on wood used as NS control deployed in a river in Shenzhen (China) for four weeks revealed that the BDP plastisphere poses a higher risk score (risk I–III) than the microbiota in the other sample types, due to its highest ARG dissemination risk. In the study, the CompRanking workflow was developed to assess the contribution of the plastisphere resistome to environmental and health risks. This resource is freely available at https://github.com/GaoyangLuo/CompRanking (accessed on 29 September 2025). The risk scale used classified as risk III contigs comprising ARGs, as risk II contigs with co-located ARGs and MGEs, and as risk I contigs with co-located ARGs and MGEs from pathogens. The risks derived for each contig type for the MPs were expressed as the ratio of contig type number to total contig number. It was observed that the MDRGs *omp*R, *opr*M, *mex*E, *gol*S, *emr*B, *mtr*D, *ceo*B, *ade*F, and the ARGs polymyxin resistance efflux pump *ros*B, the beta-lactamase IND-6, *bac*A, the aminoglycoside ARG *kdp*E, and the kasugamycin ARG *ksg*A were enriched in BDPs. Among these, *bac*A was particularly abundant and only *ceo*B and *ros*B were also moderately enriched in NBPs, along with *oqx*B, compared to water and wood. The defined AMR risk was BDPs > Water > Wood > NBPs. All particles, including wood, showed a higher prevalence of ARGs associated with MGEs than water, indicating a higher HGT risk. Moreover, in the BDPs, ARGs were mainly distributed among phages, while in the NBPs, they were mainly linked to plasmids, indicating a different contribution of transduction and conjugation to HGT in the two plastispheres. Generalized transduction by phages could lead to more efficient ARG spread in BDPs than conjugation in NBPs. The SOS-related genes *rec*A, *lex*A, *uvr*A, and the ROS-related genes *sod*B, *aph*C and *sox*R, significantly correlated with the AMR Risk Score, were more abundant in BDPs than in other sample types and a higher percentage of MAGs from BDPs carried virulence factors. Some identified genomes, such as *SYFN01*, *Xanthobacter*, and *Allorhizobium* contained MGEs close to ARGs, and are most probably involved in ARG dissemination [67].

#### The Effect of WWTP Effluents on the ARG Content of the Plastisphere

Hotspots for the association of ARGs with MPs are WWTP effluents, which can pollute water bodies if MP removal is incomplete [68]. WWTP effluents are the main source of MPs in rivers, and even those with 99.9% removal efficiency release approximately 10^5^ MP particles per day, while those with 98% removal efficiency release multiples of 10^9^ MP particles per day [69]. MPs can persist after the wastewater treatment process due to their resistance to sedimentation [70]. Moreover, MPs reduce the efficiency of ARG removal in WWTPs, which are hotspots for drug-resistant bacteria, ARG concentration, and selection [69]. In addition, it was reported that UV disinfection enhanced the conjugation frequency between the donor *E. coli* DH5α harboring plasmid RP4 and streptomycin-resistant enterotoxigenic (ETEC) *E. coli* in a solution simulating wastewater in the presence of 0.02 g/L and 0.1 g/L of PS and PLA MPs, particularly for PS at the lower MP concentration. The *E. coli* ARGs *tet*W, and *tet*C, and *int*I1 disappeared after UV treatment in the absence of MPs, which exerted protective effects on the bacterial host. Protection was attributed to the light-shielding effect of the MPs, which was more pronounced for PLA. The conjugative transfer was favored by the adhesion of bacteria to MPs, especially on PLA, which released a higher amount of carbon nutrients, and was enhanced by the upregulation of EPS export genes and the mating pair formation and replication genes *tra*F, *tra*G and *trf*Ap. Moreover, the generation of NP fragments upon irradiation increased oxidative stress and membrane permeability in bacteria, particularly for PS, which released smaller NPs [71].

A highly infectious potential of the biofilms formed on PE MPs submerged for 14 days in a WWTP effluent was demonstrated using *Galeria meillonella* larvae, into which the detached biofilm was injected. Metataxonomic analysis showed that the human pathogens *Serratia marcescens*, *K. pneumoniae*, *A. hydrophila*, *Leclercia adecarboxylata* and *Enterobacter sichuanensis* which were detected at 0.5% relative abundance before injection, became dominant in the insect 24 h post-injection, with *Serratia* spp. and *Klebsiella* spp. reaching relative abundances of 73% and 76%, and a survival reduction of 30% compared to the biofilm formed upstream from the WWTP discharge [72].

In a chemostat experiment simulating the introduction of treated wastewater into waters from Lake Maggiore (Italy), PS MPs favored the persistence of bacteria originating from the treated wastewater. Automated ribosomal intergenic spacer analysis (ARISA) showed that bacterial diversity was higher on the MPs than in the water, where diversity decreased as MP concentration increased. Quantification by qPCR of the 16S rRNA gene copies and the integrase gene *intI*1 showed that the relative abundance of MGEs in MPs increased with MP concentration in the plastisphere, but not in the water [73].

Tetracycline, which was frequently detected in WWTP effluents, was tested for degradation in the plastisphere of PVC and PLA MPs incubated for 28 days in river water. PLA and PVC adsorbed more tetracycline than quartzite, which was used as a control, and MP biofilms exhibited a simpler microbial composition than those formed on quartzite particles. The ARGs *tet*A, *tet*C, *tet*M, and *tet*X, as well as the integrase gene *intI*1, detected by qPCR, were more abundant in MP biofilms, as were the probable bacterial carriers of these genes, identified by metataxonomy as *Pseudomonas* spp., Flavobacteriaceae, and Actinobacteria [74].

Principal coordinate analysis (PCoA) of metagenomic data from LDPE and waste LDPE (W-LDPE) incubated for one week in the River Sowe (United Kingdom) showed that the most abundant bacterial genus in all biofilms was *Sphaerotilus*, an iron catcher and EPS producer that promotes the adhesion of other microorganisms. *Acinetobacter* spp. and *Aeromonas* spp. were abundant in all biofilms, while *Pseudomonas* spp., including *P. aeruginosa*, were significantly more abundant on W-LDPE, which also exhibited a higher relative abundance of ARGs. HT-qPCR targeting 48 ARGs showed that *qep*A was mainly enriched in wood and PE, and sul1 in PE and PP, incubated ex situ in a river water and sediment microcosm in the presence of azithromycin, ciprofloxacin, and sulphamethoxazole. These antibiotics were present at concentrations three orders of magnitude lower than the European Committee on Antimicrobial Susceptibility Testing (EUCAST) breakpoints (www.eucast.org, accessed on 25 July 2025), simulating concentrations found in WWTP effluent [75].

Vancomycin-resistant *A. hydrophila* and *P. aeruginosa* and sulfamethazole-resistant *B. cereus* isolated from the effluent of a WWTP in Madrid (Spain) were incubated with MPs in synthetic WWTP effluent at different total organic carbon (TOC) concentrations. On day 7, SEM showed biofilm maturation and detachment, indicating ARB dispersion. The addition of 20 µg/L vancomycin, ciprofloxacin, sulfamethoxazole, and ampicillin led to a reduction in ARB on MPs, but *sul1*, the vancomycin resistance gene *van*A, and *intI*1 were detected only in the MP biofilm and increased at TOC values above 15 mg/L. The addition of 80 µg/L antibiotics caused the disappearance of *intI*1, a decrease in *sul*1, and an increase in *van*A. In real WWTP filtered effluent, greater survival of naturally present bacteria and higher concentrations of ARGs and *intI*1 than in water were observed in PS MP biofilm. SEM showed biofilm formation after 30 days for MPs incubated in tap water, indicating that MPs can promote ARG and *intI*1 selection even in household water [76].

In PE, PET, and PP MPs, deployed individually or in mixtures upstream and downstream from a WWTP discharge for 28 days in the Mondego River (Portugal), ciprofloxacin-resistant bacteria were more abundant than in sand, particularly at the downstream site, and on PP. These bacteria belonged mainly to the genus *Aeromonas*, while those resistant to cefotaxime were mainly from the genus *Pseudomonas*. Fifty-four Enterobacteriaceae isolates resistant to antibiotics and with unique BOX-PCR profiles derived from the MP mix were identified by 16S rRNA gene sequencing, matrix-assisted laser desorption ionization (MALDI) mass spectrometry, and phenotypic tests as *E. coli*, *Citrobacter* spp., *Enterobacter* spp., *K. pneumoniae* and *Shigella* species. All except one were strong biofilm formers and most were resistant to ciprofloxacin, levofloxacin, piperacillin, ticarcillin, ticarcillin/clavulanic acid, gentamicin, sulfamethoxazole/trimethoprim, tetracycline, aztreonam, and cefepime. These harbored the ARGs *aac*A4-cr, *qnr*S, *qnr*B, *qnr*VC, and *bla*_CTX-M_, which was the most prevalent gene, with the subtypes *bla*_CTX-M-15_, *bla*_CTX-M-32_ and *bla*_CTX-M-55_ flanked upstream by an IS*Ecp1* insertion sequence and downstream by *orf*477. Two *Enterobacter* spp., five *E. coli*, and three *K. pneumoniae* strains were able to transfer the gene *bla*_CTX-M_ by conjugation to *E. coli* CV601, which acquired resistance to piperacillin, ticarcillin, aztreonam, ceftazidime, and cefotaxime [77].

Using the same experimental set-up and metataxonomy, it was shown that PP harbored a more complex microbiota than water both upstream and downstream of the WWTP, with clear PCoA separation between upstream and downstream samples, between PP and PET, and between the MP mixture and each polymer. Comamonadaceae and Weeksellaceae increased in downstream MP samples, that harbored the fecal bacterial genera *Bifidobacterium*, *Enterobacter*, and *Escherichia*-*Shigella*. The pathogenic genera *Acinetobacter*, *Aeromonas*, *Arcobacter*, *Clostridium*, *Flavobacterium*, *Legionella*, *Mycobacterium*, and *Pseudomonas* were associated with all MPs, while *Bacillus* spp. and *Treponema* spp. were associated with at least one MP but not with water. The genera *Clostridium*, *Mycobacterium*, and *Flavobacterium* were significantly enriched in PET, while *Arcobacter*, *Pseudomonas*, *Citrobacter*, and *Clostridium* were enriched in the MP mixture. The genes *aad*A2, *bla*_GES_, the quaternary ammonium resistance genes *qac*E∆1 and *qac*H, and the transposase gene *tnp*A were detected only in MPs. The total abundance of beta-lactam and sulfonamide ARGs and integrases was higher in the plastisphere than in water. The genes *bla*_CTX-M-5_, *bla*_VEB_, *bla*_VIM_, *sul*2, *ere*A, the macrolide resistance system *mat*A/*mel*, and *intI*1 were enriched in the MP mixture and in PET, which showed the highest abundance of MDRGs at both sites. The genes *aad*A2, *bla*_IMP_, *bla*_OXA-10_, *bla*_SHV_, and *sul*1 were enriched only in the MP mixture. ARGs for sulfonamides and MGEs were more abundant on PP [78].

LDPE, PET, PS, and PVC items, along with control substrates of glass and rock, were deployed for one year at a site surrounded by natural areas (site 1) and another downstream of a WWTP discharge (site 2) in the Henares River (Spain). Seven antibiotics were detected at a total concentration of about 300 ng/L at site 1, while ten antibiotics were found at nearly one hundred times higher concentration at site 2. SEM showed that biofilm began to form during the first month, with EPS present after three months, and appeared multilayered after six and twelve months. Metataxonomic analysis indicated higher diversity of bacterial populations on plastics at site 2. At site 1, diversity increased on all polymers over time except for PVC, possibly due to the release of toxic substances from this material, while at site 2, bacterial diversity decreased except for LDPE. Proteobacteria, followed by Bacteroidetes and Cyanobacteria, dominated all particles. Among the ARGs specifically sought, *erm*F was present at the highest concentrations on plastics after six months, and *qnr*SrtF11A was more abundant on plastics and glass after three months [79].

Metataxonomy showed higher microbial diversity on HDPE, LDPE, PP, and PS MPs than in water after 10 weeks of incubation in treated wastewater microcosms. In the MP biofilms *Pseudomonas* spp., *Hyphomicrobium* spp., *Mycobacterium* spp., *Flavobacterium longum*, and Rhizobiaceae were enriched, while *Stenotrophomonas maltophilia* and *sul*1 increased significantly [80].

Among PE, PET, PBAT, PLA, PBAT/PLA MP mixture, and gravel incubated in a WWTP effluent at an unspecified geographic site in China for 30 days, PE and PET became more hydrophobic and porous due to degradation. The carbonyl index (CI), defined as the absorbance ratio of carbonyl moieties to methylene moieties, increased for the BDPs. PCoA of shotgun metagenomic data showed that the microbial populations in MPs and gravel were distinct from each other and from those in water. Pathogens *P. aeruginosa*, *S. enterica*, *S. aureus*, *E. coli*, and *M*. *tuberculosis* were more abundant in the plastisphere and ARGs were four to five times higher in the biofilms. In PE, *aad*A and *ere*A, which are located on MGEs and therefore prone to HGT, as well as the rifamycin resistance gene *rbp*A, were particularly abundant. The MGE-encoded genes *qac*E1, *sul*2, *tet*X2, the aminoglycoside phosphotransferase genes *aph*(6)-Id and *aph*(3″)-Ib, and the florfenicol resistance gene *flo*R were detected in PET, PBAT/PLA, and PLA. The number of contigs encoding both ARGs and MGEs was higher in biofilms, indicating more efficient HGT. The health risks assessment (HRA) of ARGs was highest in PET, followed by PE, due to the high abundance of the gene *erm*B, a MDRG, and quinolone resistance. *Candidatus Microthrix* carrying *tet*A48, *kdp*E, and the multidrug resistance gene *rpo*B2 was the dominant host of ARGs followed by unclassified *Burkholderiales* and unclassified *Pseudomonadota*. *Acinetobacter* spp. carrying 44 ARGs, including *aad*A, *ksg*A, and *aph*(3′)-I, was associated with PET and PLA. QS functions were approximately two-fold enriched and positively correlated with the HRA in the plastisphere [69].

In the biofilm on plastic debris from the South African Msunduzi River, which exhibited total dissolved solids (TDS) and specific conductivity (SC) well above permissible limits, shotgun metagenomics revealed that Pseudomonadota was the most abundant phylum, significantly more so than in water at all sampling sites. *Pseudomonas* and *Flavobacterium* dominated the plastisphere in the industrial area, while *Acinetobacter*, *Acidovorax*, *Limnohabitans* and *Polynucleobacter* were prevalent in the agricultural area, *Undibacterium* and *Sphaerotilus* in urban areas, and *Klebsiella* spp. and *Zoogloea* at WWTP outlet sites. Alpha diversity in the plastisphere was lower than in water, possibly due to the selective pressure exerted by MPs and associated pollutants, but 26 ARG types with 372 subtypes showed an average relative abundance significantly higher than in water. These included *aac*(6′)-Im, the aminoglycoside nucleotidyltransferase gene *aad*A, *bac*A, the beta-lactamases *car*O, *cfx*A2 and *cfx*A6, the ciprofloxacin phosphotransferase *crp*P, *dfr*A1, *dfr*A14, *dfr*B2, *dfr*A2d, *qac*H, *fos*A, *fos*A5, *fos*C2, *fos*X, *ros*A, v*an*R, *van*S, *ere*A, *erm*F, *lnu*C, *mac*A, *mac*B, *msr*E, the MLS resistance genes *vat*, *vat*F and *vga*C, *rpo*B2, *abe*M, *abe*S, *acr*B, *ade*B, *ade*F, *ade*K, *ade*N, *ade*R, *mdf*A, *mex*A, *mex*B, *mex*D, *mex*F, *mex*J, *mex*L, *mex*N and *mex*T, *ugd* (*pmr*E), *arr*, s*ul*1, *sul*2, *tet*34, *tet*39, *tet*40, *tet*A, *tet*O, *tet*R, *tet*W, *tet*X, and the triclosan resistance genes *tri*A, *tri*B, and *triC*. The ARGs were mostly associated with plasmids and phages and were positively correlated with Pseudomonadota, Bacteroidetes, Actinomycetota, Bacillota, Cyanobacteria, Verrucomicrobia, Chloroflexota, Planctomycetes and Nitrospirae. Among water quality parameters, pH was associated with mupirocin and nitroimidazole ARGs, salinity with mupirocin, nitroimidazole and rifamycin ARGs, and TDS and SC with mupirocin, diaminopyrimidine, fosfomycin, diaminopyrimidine, β-lactams and tetracycline ARGs. SC was also positively correlated with nitroimidazole ARGs [81].

Long-read metagenomics using Nanopore sequencing technology (Oxford Nanopore Technology, Oxford, UK) showed that in biofilms developed over 30 days on MPs and rocks deployed in situ, as well as in water samples from the Jiuxiang River and Taihu Lake (China), alpha-diversity was highest in the plastisphere, followed by the stone biofilm. The genera *Variovorax*, *Rubrivivax* and *Thauera*, which contribute to tetracycline ARG selection, were enriched in the biofilms, and ARGs were more abundant in the MP biofilm than in water and rock, with enrichment of *mex*F, a class B beta-lactamase, *dfr*A, and *ompR* uniquely detected. Plasmid-encoded ARGs were more abundant on MPs, as were genes related to plasmid activity, DNA integration, transposition, and adhesion, indicating higher HGT efficiency. The most prevalent ARGs were rRNA adenine methyltransferases, resistance to elfamycin, and resistance to tetracyclines, associated with the taxa *Herbaspyrillum* and *Limnohabitans* [82].

At a WWTP outlet site in the Yangtze River (China), the amounts of MPs were highest, followed by those in the estuary and then at upstream sites. Smaller particles of 0.1–0.5 µm predominated and were also found downstream, while bigger particles were present only at the outlet site and not at other locations. PE, PP, PS, and PA were identified, with PE also found at other sites. Fibrous MPs were enriched, constituting about 70% of the MPs downstream. The intracellular ARGs determined by HT-qPCR were twice as abundant as upstream and were positively correlated with intracellular MGEs, including transposases, plasmids, *tnp*A, IS91, *tnp*A7 and ISI247. The extracellular ARGs were about 2000-fold higher than upstream. Intracellular ARGs *aph*-VIII and *bla*_svh11_ were detected only in the plastisphere, and extracellular ARGs *bac*A, *bla*_GES_, *cml*A1, *dfr*A1 and *qnr*S2 were introduced. Metagenome analysis identified 12 contigs with high-risk ARGs adjacent to MGEs; 8 of these were associated with MPs and 5 with the WWTP discharge site, with the *sul*1 and *tnp*A association being the most frequent. The intracellular ARGs were positively correlated mainly with the fiber shape of MPs, followed by fragments. The *Pseudomonas* genus most frequently carried *bac*A; the three genera *CAMDGX01*, *PHCI01*, and *Shewanella*, associated with fibrous MPs carried *bac*A, *nov*A, *mex*F, *mcr*-4,3, and *bla*_OXA-541_, while the genera *Pseudomonas* and *Serratia*, associated with fragments, carried *bac*A, *arn*A, *mex*B, the *P. aeruginosa* efflux pump component *mux*B, *aac*(6′)-Ic, and the aminoglycoside efflux pump *acr*D. Quantitative microbial risk assessment (QMRA) indicated that the infection risk from priority risk AMR pathogens was significantly increased by the WWTP effluent [83].

Some of the reviewed studies established associations between specific ARGs and bacterial hosts in plastic debris from freshwater, and these associations shed light on the identity of the main ARG donors and partners in HGT, with the possibility of tracing their origin based on knowledge of their typical habitats. The established ARG-bacteria associations are reported in Table 1.

### 4.2. ARG Presence in the Plastisphere in Seawater

An early study on the occurrence of ARGs in the plastisphere in seawater involved the analysis of metagenome datasets: 12 from MPs, 12 from macroplastics, and 16 from seawater, collected in the North Pacific Gyre and available from the Sequence Read Archive (SRA) of the National Center for Biotechnology Information (NCBI, https://www.ncbi.nlm.nih.gov/, accessed on 2 July 2025). ARGs were detected in all plastic-associated microbial communities but in only one fourth of the water samples. MPs and macroplastics harbored a more diverse microbiota than water and exhibited a relative abundance of ARGs approximately one order of magnitude higher, with about 10^−3^ copies per 16S rRNA gene copy. Sixty-four ARGs conferring resistance to thirteen antibiotics were identified on plastics, with MDRGs and genes for aminoglycoside resistance being the most abundant; there was no significant difference in diversity or copy number between different plastic particle sizes. ARGs were significantly associated with Flavobacteriaceae, Cyanobacteria subsection III family l, Sneathiellaceae, and Flammeovirgaceae. The identified genes were *aac*(3)-I, *aad*E, *aac*(2′)-I, *aph*(3′)-I, *bac*A, the bacitracin resistance gene *bcr*A, *bla*_TEM_ variants 1 and 185, *bla*_VEB_ variants 1, 2, 3, 4, 5, 6, and 8, *cat*, *fos*X, *fos*A, *ros*A, *ksg*A, the macrolide efflux genes *mac*A and *mac*B, *vat*A and *vat*C and D, *mex*B, *mex*E, *mex*F, *mex*I, *mex*T, *mex*W, *omp*R, *acr*B, the MDRGs *mdt*K, *bpe*F, *acr*A, *tol*C, *opr*C, *ceo*B, *sme*B, *ade*J, *ade*B, *cme*B, *mdt*B, *mdt*C, *mdt*F, *amr*B, and *cpx*R, and ARGs *tet*A, *tet*C, *tet*P, *tet*V, *tet*34, *tet*35, *tet*39, *tet*41, *van*B and *van*R. Genes for beta-lactam and tetracycline resistance showed co-occurrence in the correlation network [84].

Among 37 bacteria isolated from macroplastic samples collected in the intertidal zone of Vestland county, isolates of the fish pathogen *A. salmonicida* were resistant to ampicillin, and isolates of the opportunistic human pathogens *M. morganii* and *A. beijerinckii* were resistant to at least three classes of antibiotics. Whole-genome sequencing (WGS) highlighted the presence of class C beta-lactamases and chloramphenicol acetyltransferase *cat*B in all isolates, and class B2 metallo-beta-lactamase *cph*A in three *A. salmonicida* isolates, with a new variant in two cases. Moreover, all *Aeromonas* isolates carried the quinolone resistance gene *qnr*A. The *A. beijerinckii* isolate harbored new variants of class A beta-lactamases, an aminoglycoside acetyltransferase, and *cat* [85].

PE fragments of 5–10 cm retrieved from a stream in Vallone Casteldaccia (Italy) and from seawater in front of the stream were analyzed by metataxonomic analysis. Hundreds of operational taxonomic units (OTUs) were detected in all samples. In PE from freshwater and the surrounding water, their numbers were similar, while in PE from seawater the number of OTUs was about four times higher than in water. Bacterial phyla were not enriched in PE MPs compared to water except for Dadabacteria, Elusimicrobia, and Hydrogenedentes in MPs from seawater. The PE from the seawater and the surrounding water formed a separate cluster from PE in freshwater and the surrounding water in PCoA analysis. The ARGs *erm*B, *tet*A, *tet*W, *sul*2, *bla*_TEM_, *bla*_CTX-M_, and *qnr*S, and *int*1 were targetet by PCR. The gene *bla*_TEM_ was detected in all samples; *ermB*, *qnrS*, *sul*2, and *tetA* were found on PE from both environments; *bla*_CTX-M_ was found in PE from seawater; and *tet*W was found in PE from freshwater [86].

It was demonstrated that leachate from PVC, which contains organic and inorganic substances as well as zinc, can enrich ARGs in coastal surface water microcosms in the absence of PVC solid particles. This leachate was prepared by acid washing plastic containers and tested at two concentrations during a six-day incubation. An increase in ARG abundance, but not diversity, was observed with increasing amounts of leachate. Aminoglycoside ARGs, mainly efflux pumps, target modification, the beta-lactamase *amp*C, and MDRGs were enriched, while MLS ARGs decreased. ARGs were associated with the genera *Tritonibacter*, *Alteromonas*, a genus that can grow in plastic leachate, and *Alcanivorax*. Among these, *Alteromonas* can transmit integrative conjugative elements (ICEs) to human pathogens [87].

In a study conducted at two sites on the coast of Barcelona (Spain), where waters from WWTPs, two rivers, and recreational facilities merge, MPs collected during two cruises in 2022 contained 16S rRNA gene copies of the order of 10^6^/mm^2^. The genes *sul*1, *tet*W, and *bla*_TEM_ were detected in three to five of six MP samples collected at each site, at levels ranging from tens to hundreds of copies/mm^2^. The number of positive samples and ARG levels were lower for MPs from the sediment [88].

In PE, PP, PS, and PVC MPs deployed in a marine environment not heavily impacted by human activities near Busan City (South Korea) for 102 days, a strong biofilm formed after 63 days, particularly on PE and PP. Metataxonomic analysis showed higher microbial diversity in the MPs than in the water, with enrichment of Acidimicrobiia and Planctomycetes, while Anaerolineae were present only in MPs. The bacterial genera detected on MPs were *Methylotenera*, involved in EPS formation, *Granulosicoccus*, *Maritimimonas*, *Ketobacter*, *Pseudahrensia*, *Aquibacter*, and *Aquimarina*. MP ARGs were significantly more abundant in water except for tetracycline ARGs, including *tet*M and *tet*S. ARGs detected in MPs, in order of abundance, were *tet*A, *sul*1, *tet*C, *ermB*, *tet*Q, and *qnr*S. The genes *tet*A and *sul*1 were more abundant on PVC. The MGE *intI*1 and ARGs *erm*B, *sul*1, and *tet*C increased on PE, PP, and PS until day 63 and decreased later, while *tet*A increased until day 102. Network correlation analysis suggested that *Coxiella* spp. and *Pseudahrensia* spp. were the possible hosts of *tet*A and *tet*Q, respectively, while the genera *Fuerstia*, *Methylotenera*, *Halioglobus*, *Ahrensia*, *Rubritalea*, and *Algibacter* were potential hosts of *erm*B [89].

Shotgun metagenomics showed that in two coastal sites of the Tyrrhenian Sea (Italy)—one with minimal human impact and the other with high anthropogenic pollution—and in a pelagic site, the genera *Vibrio* and *Alivibrio* spp., *Shewanella*, and *Buchnera* spp. were the most abundant components of the biofilm on both natural organic particles and MPs sampled from surface waters. The ARG *qnr*S was present only in particles, and among MAGs associated with MPs, one identified as Rhizobiales contained *bac*A, a *Photobacterium* spp. encoded two copies of *tol*C, *acr*B, and *tet*34, and *Pleurocapsa* spp. encoded *vat*F next to MGEs [90].

MPs sampled at two sites near the seashore on Réunion Island (Madagascar)—one affected by human pollution and the other almost free from it—showed, in metataxonomic analysis, lower microbial diversity and a different abundance of genera compared to water, with cultivable bacteria counts of about 10^7^ CFU/g. Sixteen out of 105 isolates were identified by Matrix-Assisted Laser Desorption Ionization Time-of-Flight (MALDI-TOF) as belonging to the genera *Bacillus*, *Enterococcus*, *Pseudomonas*, and *Pantoea*, and exhibited non-intrinsic resistance to penicillin, ampicillin, and ticarcillin [91].

Coral reefs formed by scleractinian corals are complex ecosystems hosting at least 25% of marine organisms, which have been found to be polluted by MPs, mainly PET and cellophane (CF). PET MPs were submerged for 18 days at two coral reef sites in Hainan province (China), where the antibiotics trimethoprim, sulfaquinoxaline, florfenicol, norfloxacin, and enrofloxacin were detected, with ofloxacin and ciprofloxacin also found at one site. Biofilm formation on the MPs was confirmed by SEM, and a metataxonomic analysis highlighted a microbial population distinct from that of the surrounding water, including the genera *Acinetobacter*, *Desulfovibrio*, *Desulfopila*, IheB3-7, *Lutibacter*, *Hellea*, *Halarcobacter*, *Candidatus*_Falkowbacteria, *Neptuniibacter*, and *Rhodovulum*. Seventeen genera and five ARGs showed positive correlations in the plastisphere, among which *sul*2, the most abundant, correlated with nine bacterial genera, while the least abundant ARG was *qnr*B. All bacterial genera were correlated with at least two ARGs, and *Vibrio* spp. with *sul*1 [92].

In the touristic and fishing coastal areas of Monastir and Mahdia (Tunisia) 66 isolates were obtained from MPs separated by density from the sediment. These were distinguished by 16S—23S rRNA internal transcribed spacer fingerprinting and assigned by 16S rRNA sequencing to the genera *Acinetobacter*, *Pseudomonas*, *Bacillus*, *Staphylococcus*, *Shewanella*, *Aeromonas*, *Vibrio*, *Stutzerimonas*, *Exiguobacterium* and *Enterobacter.* Phenotypic antibiotic resistance testing identified 41 MDR profiles, comprising resistance to beta-lactams, glycopeptides, aminoglycosides, rifampicins, polymyxins and quinolones. A strain of *S. arctica*, a species able to form biofilms, showed the highest level of MDR and resistance to beta-lactams due to the presence of the *bla*_TEM_ gene, which occurred in 10% of the isolates [93].

PE and PVC panels were deployed for up to 12 months at depths of 5 m and 20 m in two bays of the Ross Sea (Antarctica), one exposed to human pollution and the other not impacted by human activities, during an Italian Antarctic campaign (2017–2018). The plastisphere biofilm was quantified using a staining technique, and bacteria were isolated and identified by sequencing a region of the 16S rRNA gene. The PVC biofilm was more abundant at the human-impacted site, while at the non-impacted site it was less abundant than that on PE. Antimicrobial resistance was determined phenotypically towards three antibiotic classes. The highest number of antibiotic-resistant bacteria in PVC was unexpectedly found at the non-impacted site at 20 m depth. On the PE panels deployed at the site impacted by human pollution, antibiotic-resistant bacteria represented 70% and 90% of the isolates from panels submerged at 5 m and 20 m depth, respectively, while at the control site without human impact, these percentages were about half. The multiple antibiotic resistance (MAR) index, defined as the number of resistances divided by the number of antibiotics tested, was generally higher for PE than for PVC; however, for PVC, a higher MAR index was calculated in the non-impacted bay. For PVC, resistance to quinolones, lincosamides, and rifamycins mostly influenced the MAR index, while for PE, resistance to beta-lactams, cephalosporins, oxazolidinones, and glycopeptides predominated. Additionally, seawater contained antibiotic-resistant bacteria (ARB) that accounted for 62–72% of isolates, indicating the presence of a resistome in an environment not contaminated by antibiotics [94].

Mangroves act as sources of pollutants, directly connecting land and sea due to their capacity to retain MPs and associated antibiotics, pathogens, and ARGs [34]. These ecosystems contribute MPs to aquatic organisms, as demonstrated by their isolation from a mudskipper fish (*Periophthalmus waltoni*) in southern Iran [95]. PP, high-density PE, PS, PET, and PCL MPs were buried in mangrove soil at three sites in Guangdong Province, China, for 30 days and reached about 10^8^ ARG copies per gram, a higher abundance than in sediment at two sites. The most frequently detected ARGs were *sul*1 and *sul*2, and at a site with higher human impact, *erm*F. The *msb*A gene was also enriched. PET showed the highest ARG enrichment; PP and PE consistently showed high ARG abundance, and PS showed greater *tet*A and *tet*T enrichment than other MPs. The *intI*2 gene was the most abundant MGE gene in PLC at one site. ARGs on MPs were significantly correlated with *intI*1 and *intI*2, as well as with human impact and environmental factors. Proteobacteria and Firmicutes predominated in most MPs and the genera *Acinetobacter*, *Bacillus*, *Desulfobulbus*, *Fonticella*, and *Vibrio* were positively correlated with ARGs [96].

At 42 coastal mangrove sites, including urban areas, aquaculture zones, and natural reserves along the southern coastline of China between April and November 2021, debris from PP, PE, polyadiohexylenediamine (PA66), polymethyl methacrylate (PMMA), PS, PET, and PVC was identified. The ARG *sul*2 was the most abundant in the plastisphere, especially in aquaculture zones. The genes *tet*A, *tet*T, *sul*2, *msb*A, and *erm*F were detected by qPCR, with absolute abundances in the range of 10^6^–10^9^ copies/g, and a total ARG level of approximately 10^17^ copies at all sites. ARG abundance was higher in urban areas, where *erm*F predominated. Among MGEs, *intI*1 was detected at absolute abundances of the order 10^4^–10^6^ copies/g. Most ARGs, except *msb*A, were positively correlated with *intI*1, indicating the potential for HGT spread [97].

After one month of incubating PE, PS, and PVC MPs in the National Mangrove Reserve in Zhangzhou (China), a significant separation was observed between the bacterial communities on the MPs and in the soil, as well as among the three types of MPs. There was a higher abundance of the Proteobacteria families Sphingomonadaceae and Rhodobacteraceae and of ARGs, mainly MDRGs, *bac*A, and *bcr*A on the MPs. High-risk ARGs of Ranks I and II, defined by the classification system of Zhang et al. [63], were more abundant in the plastisphere, with a predominance of quinolone resistance, followed by MDR and aminoglycoside resistance. The relative abundance of plasmids, integrons, and insertion elements was higher in MPs and these were positively associated with ARGs. MPs, particularly PS, showed a higher relative abundance of the pathogens *S*. *aureus*, *P*. *aeruginosa*, *Listeria monocytogenes*, *M*. *tuberculosis*, *Bordetella pertussis*, *E. coli*, and *Salmonella enterica.* The first four species predominated and were positively correlated with high-risk ARGs. Contigs of ARG-carrying plasmids were more abundant on MPs, particularly on PE, indicating HGT potential. ARGs, adhesion virulence factors, and MGEs were colocalized in pathogenic bacteria, mostly in *Pseudomonas* spp. and only in MPs [98].

Some of the reviewed studies established associations between ARGs and bacterial hosts in plastic debris in seawater, as reported in Table 2.

### 4.3. ARG Presence in the Plastisphere in Estuaries and Brackish Waters

Estuaries are characterized by salinity gradients and the presence of multiple pollution sources. PS, PP, PE, PET, and PVC macroplastics sampled at seven sites in the Yangtze Estuary (China) were found to harbor the genes *intI*, *sul*1, *tet*A, *tet*W, *aac*(6′)-Ib, and *chl*, as determined by qPCR, at higher levels than in sediment and water. The ARGs ranged from multiples of 10^6^ copies/g to 10^9^ copies/g across the sites. The average abundance of *intI*1 was about 10^8^ copies/g in the MP biofilm. The copy number of the 16S rRNA gene ranged from multiples of 10^9^ to 10^11^/g in plastic biofilms, explaining the high absolute abundance of ARGs in the plastisphere. The same polymer exhibited different biofilm communities at different sites, and the correlation between antibiotic concentrations and the absolute abundance of the corresponding ARGs was not significant. All ARGs were negatively correlated with salinity [99].

Among plastic polymers deployed for 153 days at the water/sediment interface in the Laguna Madre, a coastal lagoon and estuarine area in Mexico, PHA showed a higher relative abundance of ARGs than water, ceramic used as a control, and PET, with *dfr*E among the most abundant genes and the multidrug efflux pumps *crp*, and *mac*B, and *pmr*E among the enriched genes. The *sul* genes were present only in water and PET. MAGs and the reconstructed resistomes confirmed that the identified ARGs were abundant in PHA. In addition, the ARGs *bac*A, *fos*X and *vat*B were found in some samples. Among the isolates from PHA submitted to WGS, two close neighbors of the *B*. *cereus* and *B. thuringiensis* groups harbored ARGs for resistance to 10 and 20 antibiotic classes, respectively, and were phenotypically resistant to ampicillin, carbenicillin, cephalothin, penicillin, vancomycin, and bacitracin, with intermediate resistance to clindamycin and erythromycin [100].

MPs collected near the estuaries of the Yangtze, Sheyang, Guanhe, and Xinyi rivers (China) contained 16S rRNA gene copy numbers on the order 10^9^/g. Among the intracellular ARGs investigated by qPCR, *tet*A, *tet*M, *tet*X, *sul*1, *sul*2, *bla*_TEM_, *bla*_NDM-1_, *ere*A, and *erm*B were detected, with *sul*1 being the most enriched. Extracellular ARGs obtained from the filtrate of the cell suspension used for intracellular DNA extraction showed higher enrichment on MPs, possibly influenced by selective pressure and aging. The numbers of intracellular and extracellular ARGs were on the order of 10^8^ copies/g and 10^7^ copies/g, respectively, and both decreased at sites farther from the coast, possibly due to increased salinity. The genera *Bacillus*, *Lactobacillus*, *Pseudomonas*, *Streptococcus* and 32 bacterial species were positively correlated with ARGs. The genes *bla*_NDM-1_, *tet*A, *tet*X, *sul*1, and *sul*2 were correlated with Proteobacteria, while *bla*_TEM_, *erm*B, *ere*A, *tet*X, and *sul*1 were correlated with Bacteroidota. Redundancy analysis highlighted a positive correlation of intracellular *tet*A and *sul*1 with pH, and a negative correlation of *sul*1 and *sul*2 with total nitrogen. The extracellular ARGs t*etM*, *sul*1, *bla*_TEM_, and *bla*_NDM-1_ were negatively correlated with pH and positively correlated with total nitrogen and total phosphorus, indicating a connection with nutrient availability [101].

In the Haihe Estuary, which connects the Haihe River to the Bohai Sea (China), PET MPs of approximately 3 mm were incubated sequentially at three sites progressively closer to the sea, thus defining a “mobile plastisphere”. This plastisphere was consistently dominated by Proteobacteria, which accounted for more than 50% of the microbiota identified by metagenomic analysis, followed by Bacteroidota at roughly half that proportion. The phyla Planctomycetota, Verrucomicrobia, Gemmatimonadetes, and Nitrospirae were enriched. Thermodesulfobacteria increased during the transfer towards higher salinity, while other phyla decreased, but species-level diversity increased. MDRGs and ARGs conferring resistance to glycopeptides, beta-lactams, pleuromutilin, fluoroquinolones, triclosan, aminoglycosides, rifamycin, and bicyclomycin decreased in the sea, and the number of ARG subtypes was higher at the site just upstream. Beta-lactam and fluoroquinolone ARGs increased at the second site, and the number of subtypes remained stable at site 3. In all cases the number of subtypes followed the order beta-lactams > multidrug > glycopeptides > MLS. Among 814 ARG subtypes detected 655 persisted at all sites. Proteobacteria were the main ARG hosts, and at downstream sites, Bacteroidota hosts increased. Moreover, at the species level, bacterial ARG hosts differed between the mobile plastisphere and the sea. Pathogenic ARG carriers detected at all sites were *E. cloacae*, *K. pneumoniae*, *M. tuberculosis*, and *V. parahaemolyticus*, which decreased during transit and were not detected in the sea. Other detected pathogenic ARG hosts that also decreased were *P. aeruginosa*, *Burkholderiales*, and *Y. enterocolitica*. The PPR model indicated that estuarine environments reduce the ARG risk originating from land sources. Nevertheless, the AMR risk at site 3 remained high and could be transferred to the marine ecosystem [102].

At four sites in a pharmaceutical and chemical industrial park in the Yangtze River Delta region (China) rubber, polyisoprene chlorinated (PLC), and PE were detected in groundwater samples obtained from wells, representing approximately 61%, 9%, and 8% of the MP abundance, respectively. Rubber and PLC were positively correlated with the antibiotics roxithromycin, sulfamethazine, and clindamycin. PE and PLC were positively correlated with *sul*1, *erm*F, and *intI*1, while rubber and PA were negatively correlated with the ARGs. The microbial groups identified by metataxonomic analysis—Comamonadaceae, *Acinetobacter*, *Pseudomonas*, *Simplicispira*, and *Proteiniphilum*—showed significant positive correlations with ARGs [103].

In the fresh and brackish waters near the metropolitan areas of Tokyo, Saitama and Chiba (Japan), the highest levels of MPs were found near the outlet of the Arakawa River. The most abundant MPs were PE and PP, followed by PA, with smaller amounts of PS, PET, PVC and PU. Shotgun metagenome analysis revealed lower bacterial diversity on MPs than in the water, with selected bacterial populations including the genera *Exiguobacterium*, *Eubacterium*, *Dolichospermum*, *Anabaena*, *Gloeothece*, *Nodularia* and *Planktothrix*. Among 1110 ARGs detected, 38 were found exclusively on MPs, with different relative abundances in the Tone River and the Arakawa River. The most abundant ARG in MPs was the MDRG *rsm*A, followed by *qac*G, *van*T, *van*Y, *van*W, *van*H, *bla*_OXA-53_, *bla*_OXA-912_, and *bla*_IND-6_. ARG subtypes present only in MPs included *bla*_OXA-912_, *bla*_OXA-726_, *fos*A8, and the aminoglycoside resistance genes *rmt*F and *apm*A. MGEs, including subtypes of *is*_*tn* and phage genes, such as *rev*, *rev*_3, phage_integrase, *tnp*R, *Relaxase*, *Int_CTnDOT*, Tn916, and *Phage_GPA*, were more abundant in the plastisphere than in water. Correlation network analysis showed that the genera *Citrobacter*, *Aeromonas*, *Sulfitobacter*, and *Lacinutrix* were potential hosts for *bac*A, *Acr*AB-*Tol*C, a mutated *mar*R conferring resistance to ciprofloxacin and tetracycline, the *acr*AB inducer *mar*A, *van*G, *ade*F, *qac*G, and *van*H. *K. pneumoniae* was identified as the probable host of a mutated *kpn*F efflux pump conferring resistance to most antibiotics [104].

PE, PP, and PS MPs with sizes of 3 mm, 200 µm, and 30 µm, deployed for two months in Xinglin Bay (China), had a z potential, which depends on the electric charge at the interface particle/water, ranging from −48 mV for 30 µm particles to −5 mV for 3 mm particles. The specific surface area, calculated using the Brunauer–Emmett–Teller method, was up to ten times larger for the 30 µm MPs. The biofilm detached from the smaller particles showed an OD_595_ value five to nine times higher than that from the 3 mm MPs. The most abundant ARGs in the MP biofilms encoded MDR, bacitracin, and sulfonamide resistance, and the ARG profiles changed with MP particle size for PE and PP. Shotgun metagenomics showed that the composition of the microbiota did not vary significantly with MP particle size, and the dominant bacterial groups—Proteobacteria, Actinobacteria, Firmicutes, and Bacteroidetes—exhibited higher diversity than in water. The pathogens *P. aeruginosa*, *L. pneumophila*, *K. pneumoniae*, *M. tuberculosis*, *A. hydrophila*, *A. baumannii*, *B. pseudomallei*, and *Brucella melitensis* were more abundant in the MPs. The co-occurrence of *intI*1 with the genes *aac*(6′)-I, and *bac*A, and *ICEKpn342*-1, as well as *intI*1 with *mex*T on the same contigs, was observed. The z potential, and contact angle, a measure of the liquid–solid interface tension, were negatively correlated with ARG abundance. The risk score, calculated by considering the occurrence of ARGs, MGEs, and pathogens, increased with particle size for PS and PP, while the opposite trend was observed for PE [105].

PE, PLA, PS, PVC, MPs, and Tetra Pak incubated in the same bay for 50 days showed a higher risk of ARG transmission compared to wood, rock, and glass, as demonstrated by combining single-cell spectroscopy and HT-qPCR performed on a SmartChip Real-time PCR system with 384 primer pairs for ARGs, taxonomic markers, MGEs, and the 16S rRNA gene. The biofilm on wood was the most abundant, but positive co-occurrence correlations were more numerous and correlation networks less complex among microbial groups in MPs. Metataxonomic analysis of bacteria and fungi, along with the neutral community model (NCM), indicated that deterministic processes such as species interaction and niche differentiation influenced microbial assembly on MPs, which presented a ten-fold higher health risk for the proportion of metabolically active hosts of high-risk ARGs [106].

D_2_O-labeled single-cell Raman spectroscopy applied to microbial communities challenged with ciprofloxacin and the last-resort antibiotics colistin and meropenem showed that ARB for these antibiotics were more abundant on MPs than on natural surfaces. Tetra Pak, PS, and PLA showed a higher abundance of ciprofloxacin-resistant bacteria, PLA a higher abundance of meropenem-resistant bacteria, and all MPs a higher abundance of colistin-resistant bacteria. Moreover, the colistin resistance gene *mcr*1 was connected to 32 bacterial genera, some of which positively correlated with the MGEs *mob*A, *Inc*Q_*ori*T, and *orf* 37-IS26, indicating HGT potential. The genotypic health risk index (GHRI) for ARGs, which depends on abundance in the environment, mobility, association with pathogens, and resistance to antibiotics of clinical relevance, was highest for Tetra Pak, followed by wood and then the MPs, and was mainly influenced by ARGs for aminoglycosides, tetracycline, and MDRGs. The integrated health risk index (IHRI), which takes into account biofilm biomass, metabolic activity, ARGs present, phenotypic AR, and pathogen abundance, was nine- to twenty-fold higher for Tetra Pak, PLA, and PS, mainly influenced by phenotypic AMR as the dominant factor, followed by surface hydrophobicity [106].

Some studies have established associations between ARGs and bacterial hosts in plastic debris in estuarine waters, as reported in Table 3.

### 4.4. The Role of Viruses in ARG Transmission in the Plastisphere

A role for transduction in the HGT of ARGs in plastic debris was indicated by their presence in the genomes of viruses detected in the plastisphere, as observed in a study on PE, PP, PS, PE-fiber (PF), and PE-fiber-PE (PFP) deployed at five sites upstream and downstream from the estuary of the Bei-Lun River (Vietnam) for 30 days. Metagenomic analysis showed that the viral community was distinct among polymers but not among sites, and the abundance of each virus varied among sites. For example, the circular DNA virus-25, associated with sewage, was more abundant in the estuary. Eleven ARGs were detected in viral genomes, among which the fluoroquinolone resistance gene *gy*rB4 was the most abundant. Moreover, 90% of the virus-associated ARGs were found in PP upstream of the estuary and in PE downstream and in the estuary. The viruses detected in PP harbored *vat*B, *dfr*E, *rpo*B, *rpo*C, *gyr*B1, and *gyr*B2, while those detected in PE harbored *gyr*B3, *par*C, *par*E, *gyr*B4, and *gyr*A. The PPR model, by taking into account the ARGs and virulence factors in the viral genomes, indicated that the potential risk related to the MPs was highest for PP and positively correlated with its highly positive z potential which favors the binding of negatively charged DNA [29]. Moreover, network correlation showed more numerous interactions between viruses and bacteria on PP [107].

In the biofilm formed over 21 days on PE MPs deployed in the Xixuan Island sea (China), metagenomic analysis showed the presence of 4999 phages, of which 609 were associated with the genera *Vibrio* and *Bacillus*. Twenty-eight ARGs encoding resistance to MLS, aminoglycosides, beta-lactams, bicyclomycin, fosfomycin, and multiple drugs were found in the genomes of the DNA viruses, while in the RNA viruses the ARG *omp*K37, encoding a beta-lactamase from *K. pneumoniae*, was identified [108].

From the analysis of 180 publicly available plastisphere metagenome datasets from different environments, 4062 phage contigs were identified. These were significantly associated with pathogens and ARB, mostly in BDPs, and encoded ARGs that can be horizontally transferred [109]. This underlines the importance of further investigating the role of viruses in ARG HGT in the plasisphere in aquatic environments.

**Figure 1:**
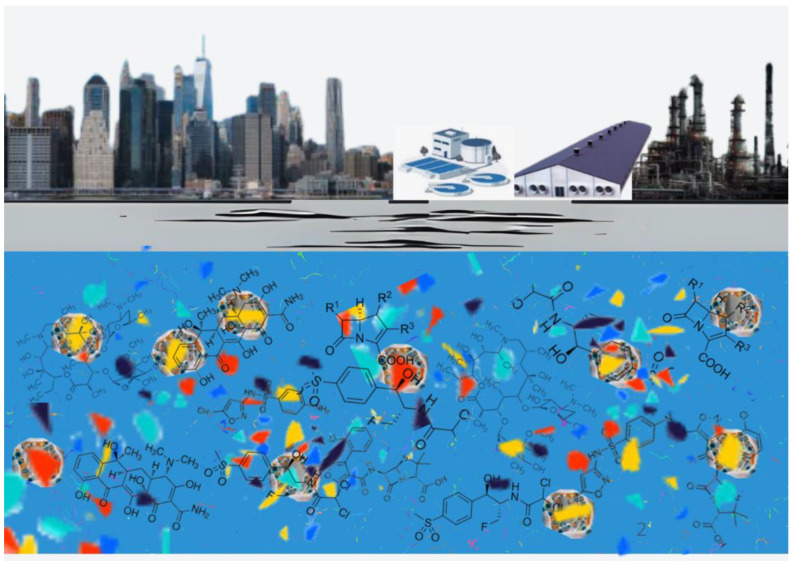
Various pollution sources contribute plastic debris (colored particles) and antibiotic residues (dispersed molecules), leading to the selection of ARB (coating on plastic debris) in the plastisphere in water bodies [45,46,53,61,66,69,77,78,79,81,83,88,91,94,102,104].

Figure 1 summarizes the multiple lines of evidence for the effect of plastic debris and antibiotic pollution from various sources on the selection of ARB in the plastisphere in water bodies.

## 5. Effects of MPs on ARG Selection in Aquatic Animals and Fishery Products

### 5.1. Presence of ARGs in MPs in Aquaculture Farms

MPs colonized by ARB in aquatic environments can directly contaminate fishery products and transmit ARGs or induce ARG selection in the intestinal tract of animals, but this connection has been investigated only to a limited extent. Aquatic animals are a major source of MPs in the human diet, as fish ingest MPs by mistaking them for food and through trophic transfer. Additionally, MPs accumulate in filter-feeding molluscs. Notably, the European Food Safety Authority, in a scientific advice on contaminants in the food chain, warned that a 225 g portion of Chinese mussels can contain up to 900 MP particles [110]. MP contamination can reach human consumers through various stages of aquatic trophic transfer, in which mussels, clams, and crabs are primary consumers, small fishes are secondary consumers, and predatory fishes are tertiary consumers [111]. Fishes that feed on a wide range of prey are more likely to accumulate MPs than those with selective feeding habits [108]. Moreover, MPs have shown detrimental synergistic effects with the veterinary antibiotics oxytetracycline, florfenicol, and sulfamethoxazole on *Mytilus coruscus* mussels, including increased antibiotic accumulation in tissues [112].

Aquaculture is a significant source of MPs in aquatic environments due to the extensive use of plastic items such as fishing nets, floats, and packaging materials [113]. The effects of MPs in aquaculture include environmental degradation, toxicity, and the selection of ARGs which ultimately lead to reduced productivity in these systems [114]. Mariculture, that is, aquaculture practiced near the coasts, is a hotspot for ARG selection because of the substantial use of antibiotics, which are often added to the feed. In some countries, mariculture effluent, which may contain antibiotic residues, is discharged directly into the sea, where these residues are diluted to sublethal concentrations that promote the enrichment of ARGs such as *sul*1, *sul*2, and the *tet* genes [115].

Antibiotics are adsorbed by plastic debris, as demonstrated by a study on PS, PP, PE, PET, and PVC MPs aged for eight weeks in a scallop farm, an abalone farm, and at a site without mariculture farms in Dongshan Bay (China). A maximum antibiotic concentration of 26 ng/g was detected in MPs, with PP showing the highest adsorption rate for sulfanitran, followed by danofloxacin, marbofloxacin, josamycin, tetracycline, and doxycycline. The diversity of the MP-associated bacteria was higher than in water, with a predominance of Proteobacteria and Bacteroidetes, and the genus *Vibrio*, followed by Firmicutes and Actinobacteria. PCoA indicated the distinctness of the MP microbiota from that of water and between the two mariculture farms, but not among the MP types. The human health risk quotient (HQ) posed by antibiotics adsorbed on MPs was derived from the estimated daily intake (EDI) and the acceptable daily intake (ADI), and josamycin in scallops reached an HQ value of risk [116].

In a recirculating mariculture plant in Fujian Province (China), PET fibers were the dominant MP type in biofilter water, recycled water, and fish ponds, and were released by the biofilter made of this material. The number of copies of the 16S rRNA gene on the MPs from the three compartments was of the order 10^7^–10^8^/g, and the absolute abundance of ARGs determined by qPCR was of the order of 10^9^ copies/g with *sul*, *tet*, *qnr* and *erm* genes at 3–4 orders of magnitude higher than in water. The genes *tet*X, *qnr*A and *erm*F predominated but *tet*B, *tet*G, *qnr*B, and *qnr*S were also detected. The *intI*1 indicator of HGT potential was three orders of magnitude higher on MPs than in water. The microbial phyla detected—namely Proteobacteria, Bacteroidetes, Planctomycetes, Chloroflexi, Actinobacteria, Firmicutes, Chlorobi, Cyanobacteria, and Spirochaetae—were more abundant on MPs, and species richness was higher than in water. Twenty-five bacterial genera were present only on MPs or were enriched on MPs, and were positively correlated with *intI*1 and at least five ARGs, including *sul*1, *sul*2, *tet*G, *erm*F, and *qnr*S [117].

Similar results were obtained for a recirculating mariculture farm in Yantai (China), where fibrous PET MPs derived from the filter were the most abundant MPs in all compartments. Cultivable bacteria reached multiples of 10^8^ CFU/g on MPs, three orders of magnitude higher than in water, at all sampling points, and showed a higher percentage of ARB resistant to chloramphenicol, tetracycline, and sulfafurazole, with a minority resistant to gentamicin, ciprofloxacin, penicillin and erythromycin. Based on metataxonomic analysis of bacteria grown in the presence of antibiotics, the microbial population of ARB on MPs was more diverse than in water, with a predominance of Proteobacteria and Bacteroidetes, and the genera *Vibrio*, *Muricauda*, *Ruegeria* and *Sunxiuqinia*. Among these, *Maricauda* carried the highest number of ARGs, followed by *Ruegeria* and *Vibrio* and the most frequently detected ARGs were *flo*R, *sul*2, and *bla*_SHV_. Multiresistant bacteria (MARB), among which the pathogen *V. alginolyticus* predominated, were also more abundant on MPs. The most frequent multiple resistance profile included resistance to tetracycline, sulfurazole, erythromycin, and penicillin, followed by the same profile plus resistance to chloramphenicol. The integrase gene *intI*1 was detected in 83.8% of MARB isolates and was associated with *aad*A2, *aad*A5, *aad*B, *dfr*A1, *dfr*A27, *ere*A and *arr*2 [118].

In another study on a mariculture plant, the distribution and relative abundances of ARGs and MDRGs in water and MPs were not significantly different. The most abundant ARG encoded chloramphenicol resistance, and *intI*1 was the most abundant MGE indicator [119].

Oysters, an important aquaculture product, accumulate MPs through filtration. Oysters were exposed for 30 days to PE, PP, PET, polyhydroxybutyrate (PHB), PLA, and control particles of wood and glass at a farm in Zhenhai Bay (China). Biomass growth was slower on NBPs but after 14 and 30 days it was comparable to that on BDPs and SEM imaging highlighted the formation of EPS. Metataxonomic analysis showed that microbial diversity on MPs became higher than in water by day 14. The Proteobacteria families Rhodobacteraceae, Comamonadaceae, and Pseudomonadaceae, and by day 30 also Flavobacteriaceae and Sphingomonadaceae, were the most abundant on MPs and were more prevalent than in water. Biomarker genera for PE were the hydrocarbon-degrading *Alcanivorax*, the polycyclic aromatic hydrocarbon degrader *Erythrobacter*, and the potentially pathogenic genus *Tenacibaculum*; for PLA, *Hydrogenophaga* and the potential pathogen *Pseudoalteromonas*; and for PP, *Candidatus Amoebophilus*. The relative abundance of *sul*1, *qnr*S, and *bla*_TEM_ was lower in MPs than in water due to the higher biomass, but the relative abundance of *intI*1 was higher in MPs, indicating a high risk of ARG HGT [120]. Moreover, *P. aeruginosa*, a Priority 1 ARB pathogen [121], was enriched in MPs [120].

PLA MPs and florfenicol were found to alter the ARG profile of the phytoplankton component *Chlorella pyrenoidosa*, whose phycosphere hosts bacteria and ARGs and plays a role in their spread in rivers. The exposure lasted 21 days in fish pond water, and qPCR showed that treatment with PLA MPs and florfenicol increased the abundance of *sul*1, *sul*2, *flo*R, *fex*A, *fex*B, the oxazolidinone resistance gene *optr*A, *cfr*, and the phenicol resistance gene *pex*A, and MGEs *int*I1, IS613, *tnp*A01, *tnp*A02, and *tnp*A03 in the algal phycosphere by more than two-fold, reaching multiples of 10^10^ copies/L, with a relative abundance increase of two orders of magnitude. Metataxonomic and co-occurrence analyses revealed that, in the phycosphere, *Devosia*, *Flavobacterium*, *Hydrogenophaga*, and *Methylophylus* were potential hosts for ARGs [122].

Samples of water, sediment, plastics, and fish intestines were collected from a *Sciaenops ocellatus* farm in Mauritius, where fish were reared in open cages at varying distances from a river estuary and a lagoon channel. Metataxonomic analysis revealed that Proteobacteria abundance in floating macroplastics was higher than in water, sediments, and fish gut, with higher diversity of pathogenic microorganisms at the estuarine site and 30 bacterial species shared with the fish microbiota. Among these, *V*. *alginolyticus*, *Photobacterium damselae* and *S*. *epidermidis* were isolated. Most Vibrionaceae exhibited high phenotypic resistance to ampicillin and tircacillin, and about 3% were resistant to norfloxacin, ciprofloxacin, ofloxacin, and levofloxacin, but none were resistant to the antibiotics used on the farm. The MAR index was higher for plastic-associated bacteria [123].

In mariculture sediments, MPs can reach concentrations of the order of 10^5^ particles per kg, at least ten times higher than in other sediments. PS and PVC particles, ranging in size from 0 to 120 µm and 0.5–2.0 mm, were incubated for 60 days in the presence of tetracycline and sulfamethoxazole at concentrations typically found in water and sediment collected from an aquaculture farm in Jiaozhou Bay (China). The microbiota present in the sediments was pre-adapted to these antibiotics, and the abundance of 13 ARG, determined by qPCR, increased to a maximum of 88%, reaching the order of 10^8^ copies/g. Among these was the *flo*R gene, known to be harbored by *Salmonella* spp. and previously reported in seafood products. The greatest distinctness in ARG profiles was observed between the PS and PVC particles. In PS, *tet*W was selectively enriched, while in PVC, the absolute abundance of *sul*1, *sul*2, and *sul*3 increased. These findings are consistent with previous reports of preferential adsorption of tetracycline on PS and sulfamethoxazole on PVC. The ARGs *qac*H01 and *flo*R were significantly enriched in PS, whereas *mat*A, *bla*_TEM_, *amp*276, *aad*A5, and *aad*E were significantly enriched in PVC. The relative abundance of *intI*1 and the transposase gene *tnp*A increased in the presence of MPs and was positively correlated with the ARGs [29]. 

Metataxonomic analysis showed significantly lower bacterial diversity in mariculture sediments exposed to PVC, while exposure to PS enriched Proteobacteria, Bacteroidetes, Gemmatimonadetes, and Firmicutes. The neutral and null models indicated that the PS treatment enhanced the migration of microbiota components and ARGs to the sediment. Both polymers, PVC to a higher extent, promoted the assembly of the microbiota through deterministic processes, likely due to selective pressure exerted by the release of Zn, Cu, phthalates, and BPA, and both polymers favored an increase in ARG hosts. Network correlation analysis indicated that PS enriched *Ralstonia*, *Planctomicrobium*, *Vulcanibacillus*, *Desulfitibacter*, and *Conexibacter* carrying *flo*R, *aad*A5, and *bla*_TEM_, while PVC enriched *Tistlia*, Subgroup_10, *Candidatus*_*Alysiosphaera*, and *Pelagibius* carrying *tet*W, *sul*3, *mat*A, and *bla*_TEM_. At the biofilm maturation stage, both polymers were completely coated with bacteria, and the 16S rRNA gene copy number reached multiples of 10^12^/g, but was significantly lower for PVC and higher on smaller particles, which are most likely ingested by benthic invertebrates such as mussels and clams [29].

In a high-density farm producing the shrimp *Penaeus vannamei* on the Guangdong coast (China), PE MPs of three sizes were deployed to investigate the presence of ARGs after two months. On the large particles level-I-risk ARGs, including the genes *bla*_LCR-1_, *cml*A5, the MLS resistance gene *lin*G, *aph*(3″)-Ib, *ant*(6)-Ia, *aph*(6)-Id, and *aac*(6′)-Ib9, were significantly more abundant than in water. On MPs, the dominant bacterial genera differed from those in water, with the most abundant being *Paracoccus*, *Thauera*, *Thiobacillus*, *Halothiobacillus* and *Ruegeria*. The pathogenic genera *Brucella*, *Brevundimonas*, *Escherichia*, *Enterococcus*, and *Pseudomonas* were significantly more abundant than in water. The genera *Brucella* and *Brevundimonas* were positively correlated with *cat*B3, and *Pseudomonas* with *qac*H. On smaller MPs, Sphingomonadales, Pirellulales, Desulfobulbaceae, and Desulfocapsaceae, and the genera *Vibrio*, *Kangsaoukella*, *Roseobacter*, *Roseovarius*, and *Shimia*, were enriched while Rickettsiales, and the genus *Granulosicoccus* were enriched on medium-sized particles. A significant positive correlation was found between TOC and ARG abundance, and a negative correlation was found between MP roughness and ARG abundance [114].

PBAT and PP MPs were deployed for three months in a *Tilapia* aquaculture farm in Haikou (China), where 3000 fish were raised and antibiotics were used rarely, only in cases of disease during the study period. The giant viruses *Pandoraviruses* which undergo gene duplications and are prone to HGT with their bacterial hosts, prevailed on MPs. The Chao1 index and the Abundance-based Coverage Estimator (ACE) were higher for PBAT than for other MPs, and the biomass was higher for PHA and PLA. Bacterial assembly in MPs was driven by deterministic processes, as shown by the Raup–Crick box plot, compared to the other matrices analyzed, i.e., water, sediment, and fish gut. The abundance and number of subtypes of ARGs were higher in MPs, and *bac*A and *sul*2 were particularly abundant in PP. Moreover, MGEs were more abundant in PP, and the *tnp*A transposase was more abundant in PBAT and PP. Rank I ARGs associated with the ESKAPE pathogens (*E. faecium*, *S. aureus*, *K. pneumoniae*, *A. baumannii*, *P. aeruginosa* and *Enterobacter* spp.) and pathogens classified as Priority 1 (carbapenem-resistant *A. baumannii*, carbapenem-resistant *P. aeruginosa*, carbapenem- and third-generation cephalosporin-resistant Enterobacteriaceae), Priority 2 (vancomycin-resistant *E. faecium*, methicillin- and vancomycin-resistant *S. aureus*, clarithromycin-resistant *Helicobacter pylori*, fluoroquinolone-resistant *Campylobacter* spp., fluoroquinolone and third-generation cephalosporin-resistant *Neisseria gonorrhoeae*) [62], and Priority 3 fluoroquinolone-resistant *Shigella* spp. were found at higher levels in PBAT. Network correlation analysis showed stronger correlations between ARGs and microbial groups in MPs [124].

### 5.2. Selection of ARGs in Aquatic Animals Induced by MPs

An indirect mechanism by which MPs drive ARG selection and transmission in animal hosts is through inducing changes in the gut microbiota, leading to the emergence of ARG carriers and activation of pathways that favor ARG spread. The zebrafish, *D*. *rerio*, serves as a model for studying the impact of various factors on the intestinal microbiota, as it is easy to breed, has a short life cycle, and its intestinal structure and function are similar to those of the human gut. Oxytetracycline was combined with MPs and NPs to evaluate toxicity effects in this animal. After a 30-day exposure, HT-qPCR with 384 primer sets targeting ARGs, MGEs, and the 16S rRNA gene showed that the normalized copy numbers of ARGs and MGEs in the intestinal microbiota were significantly higher than in the control for treatments with NPs, MPs, and NPs combined with oxytetracycline. The enriched ARGs in treatments with MPs and NPs alone, and in those combined with oxytetracycline, differed, with MDRGs more abundant in the MP and NP treatments. The microbiota composition was determined by metataxonomic analysis, and Pseudomonadales and Burkholderiales were positively correlated with ARGs [125].

Exposure of the crucian carp *Carassius auratus*, a freshwater fish, either separately or in combination to roxithromycin and PS MPs, not degraded or exposed to UV light with the formation of hydroxyl groups and modification of the carbonyl region in the Attenuated Total Reflectance FTIR (ATR-FTIR), resulted in an increased concentration of the antibiotic in the fish intestine, which was higher in the presence of MPs and even greater with aged MPs. Moreover, inflammatory cell infiltration was observed in the intestine after exposure to the combined aged PS MPs and roxithromycin, while cilia defects were observed after exposure to the antibiotic combined with non-degraded PS MPs. Metagenome sequence analysis showed that the genera *Gemmobacter*, *Bosea*, *Rhizobium*, and *Shinella* increased in the intestinal microbiota after exposure to the MPs/antibiotic combination. Roxithromycin exposure led to an increased number of ARGs encoding resistance to bacitracin, tetracycline, sulfonamide, and chloramphenicol, and its combination with MPs led to an increase of *sul*1, which was positively correlated with different microbial genera [126].

In *C*. *auratus* exposed to aged MPs (AMPs) and roxithromycin for 14 days, antibiotic accumulation in tissues and organs, especially the gut, brain and kidney, was higher with smaller MPs. Compared with exposure to the antibiotic alone, the presence of AMPs of 0.5 µm, 5 µm and 50 µm led to an increase in *Proteobacteria*, *Fusobacteriota* and *Firmicutes* in the fish gut and reduced the abundance of *Cetobacterium*, a probiotic genus that produces vitamin B12, butyrate and acetate, while *Shinella*, *Paracoccus*, *Gemmobacter*, *Pseudorhodobacter*, *Vibrio* and *Aeromonas* increased [127].

*Oryzias melastigma*, or marine medaka, euryhaline fishes, were exposed for four weeks to PS MPs at approximately 10^8^ particles/m^3^ and tetracycline at 43 ng/mL—doses possibly found in the environment—either individually or in combination. Metataxonomic analysis showed that exposure to tetracycline, with or without MPs, increased the levels of Proteobacteria and Bacteroidetes, decreased the levels of Firmicutes and Actinobacteria, and altered the relative abundance of 15 bacterial genera in the intestinal microbiota. Among 35 tetracycline resistance genes assessed by HT-qPCR, *tet*D, *tet*G-02, *tet*O, *tet*Q, *tet*R, *tet*W, *tet*35 and *tet*34 were detected in the group exposed only to MPs, while *tet*O, *tet*R, *tet*W and *tet*35 were found in the tetracycline/MP combination group. Therefore, it was concluded that the increase in the number of *tet* ARGs in the group exposed to PS MPs alone was a consequence of their effect on the intestinal microbiota [128].

Biofilms in MPs with adsorbed oxytetracycline were artificially produced and tested for their effects on zebrafish after 30 days of exposure, compared with biofilms on MPs without adsorbed oxytetracycline, MPs alone, and a control. Metagenome sequence analysis showed that the relative abundance of intestinal Proteobacteria increased after exposure to MPs with biofilm, with or without oxytetracycline, while Fusobacteria increased mainly after exposure to MPs with biofilm and adsorbed oxytetracycline. The number of ARG subtypes significantly increased in all MP treatments, with or without biofilm, reaching 104 in the group exposed to MPs with biofilm and 101 in the group exposed to MPs with biofilm and oxytetracycline. MDRGs were the most numerous resistance genes in each group, followed by ARGs for macrolides, vancomycin, and tetracyclines, in that order. Furthermore, a strong correlation was found between pathogenic bacteria and ARGs [129].

Exposure for eight weeks to PVC MPs combined with either sulfamethazine or oxytetracycline had no significant effects on the body weight or length of carp (*Cyprinus carpio*), but metataxonomic analysis revealed effects on the composition of the intestinal microbiota. Groups exposed separately to PVC, sulfamethazine, oxytetracycline, and PVC/sulfamethazine showed higher gut microbiota diversity than the group exposed to PVC/oxytetracycline, with the PVC/sulfamethazine group exhibiting the highest alpha-diversity. Exposure to PVC or the antibiotics alone induced an increase in *Fusobacterium* spp. and Firmicutes, which was further enhanced in the PVC/oxytetracycline group. The genus *Enterobacter*, which hosts the MDRGs *acr*A-05, *acr*R-01, *tol*C-02, *tol*C-03 and tetracycline ARGs *tet*D-01, *tet*D-02, and *tet*R-02, was more abundant in the groups exposed separately to PVC and sulfamethazine. HT-qPCR showed that 101 ARGs and eight MGEs were present in all groups, but in higher numbers in the single antibiotic and PVC/sulfamethazine groups. Treatment with PVC/oxytetracycline enriched tetracycline resistance genes, mainly *tet*M-01, *tet*M-02, and *tet*PB-03, while PVC/sulfamethazine treatment led to an increase in *cmx*A. Most ARGs were associated with Proteobacteria and Actinobacteria, and the composition of the microbial population had a greater influence on ARG distribution than on MGE occurrence [130].

ARG HGT in the mussel *M*. *galloprovincialis* was investigated following exposure to PE MPs covered by a biofilm formed in a seawater-simulating solution by *E. faecium* UC7251 and *L*. *monocytogenes* strains Scott A and DSM 15675. The *E. faecium* strain harboring *ant*(6)-Ia, *aph*(3′)-III, *aad*(6)-Ia, *ant1*, *erm*B, *mrs*C, *sat*A, *isa*E, *lnu*B, and *tet*L in an integrative and conjugative element (ICE) present on a mobilizable megaplasmid, and the transposon Tn916 carrying *tet*M on the chromosome, was used as the MDR ARG donor, while *L. monocytogenes* strains were used as pathogenic recipient representatives. Conjugation with HGT of *tet*M and *erm*B did not occur between planktonic cells, but transfer of *tet*M occurred in the MP biofilm and in mussels exposed to the MPs, to a higher extent than in water. It was therefore concluded that the MP concentration resulting from the filtering activity of mussels favored ARG HGT [131].

The widely used antibiotic ciprofloxacin has been detected in various aquatic environments at concentrations ranging from a few to hundreds of µg/L, and between 9.1 and 14.54 µg/kg in Asian green mussel *Perna viridis* farmed along the Chinese coastline. In shellfish aquaculture, the presence of MPs is mainly due to the use of plastic structures, such as rope cultivation supports. The intestine of *P*. *viridis* can accumulate MPs at average levels of 10 particles per mussel, with a surface area of about 3 m^2^/g, pores of approximately 20 µm, and prevailing PP composition. Exposure to artificial MP particles at a concentration of 0.6 mg/L and to 1 µg/L ciprofloxacin, reflecting environmental levels, resulted in a 60% higher concentration of the antibiotic in mussels compared to exposure to ciprofloxacin alone, with an average final concentration of 142 µg/kg wet weight. Metagenomic analysis revealed a decrease in the genus *Spirochaeta* and lactic acid bacteria, and an increase in the pathogenic genera *Treponema* and *Vibrio* in mussels exposed to the antibiotic. Rather than a change in the types of ARGs, a shift in ARG carriers was observed as an effect of selective pressure; for example, MLS ARG carriers shifted from Paenibacillaceae to Lachnospiraceae. The target hazard quotients (THQs) and cancer risk (CR) indicated a negligible toxic risk associated with frequent or occasional consumption of the contaminated mussels by children and adults, but these risks were significantly increased. After cooking, mussels exposed to MPs and ciprofloxacin exceeded the lower value of the Minimal Selective Concentration (MSC) of the antibiotic, which is a concentration below the MIC that can select for resistant bacteria as the dietary exposure dose for the human gut microbiome (DEGM) [132].

The enhancement of veterinary antibiotics oxytetracycline and florfenicol accumulation in edible bivalve molluscs upon exposure to MPs was observed and explained by the suppression of detoxification genes. The level of accumulation was well below the estimated THQs, but the DEGMs for the antibiotics tested were greater than or similar to the MSC, thus indicating a risk for AMR development [133].

Macrobenthic invertebrates such as shrimp, shellfish, and snails retain plastic particles, mostly NPs, from the surrounding water at a rate proportional to their biomass. Different species of aquatic invertebrates were found to carry ARGs. In spiral shells at different sites, NPs were observed only in the animal gut, while bigger particles were present in significantly lower amounts than in the sediment. PET, PP, PU, PVC, PA and PS MPs were found in comparable concentrations in the shellfish gut and in the sediment. All benthic macroinvertebrates, particularly *Chironomidae* larvae, showed higher MP concentrations than those found in water and in the guts of benthic fish. Among ARGs specifically detected by qPCR, *tet*A, *sul*1, *sul*2, and *sul*3 were consistently found at higher levels in spiral shells, and the gene *tet*A showed a higher ratio between copy number and number of plastic particles, as well as a negative correlation between copy number and particle size. To elucidate the effect of MP degradation on the increase in ARG copy number, *Chironomidae* larvae were fed with PE MPs of approximately 42 µm and *E. coli* K12 MG1655 harboring the conjugative plasmid RP4. The number of MP particles and their size decreased during the experiment as a result of degradation. The copy number of the plasmid gene *tra*G, determined by qPCR, increased in the presence of MPs, particularly when the *E. coli* strain was fed after 15 days of MP exposure, indicating enhanced conjugative transfer following MP degradation. Moreover, the conjugative genes *trf*Ap and *trb*Bp, and the SOS response genes *rec*A, *pol*B, *ruv*A, *rec*N, and *uvr*B were strongly upregulated in the gut of the *Chironomidae* larvae upon exposure to MPs and *E. coli*, more so than upon exposure to *E. coli* alone, especially after 15 days of MP feeding. In an *E. coli deletion* mutant for the *lex*A *repressor*, the HGT of plasmid RP4 was more efficient than in the native strain, confirming the involvement of the SOS response [134].

An in situ exposure experiment of *M. coruscus* to PE MPs was conducted on Xixuan Island (China) for 21 days to evaluate the effect on the viral community composition in the digestive gland. Shotgun metagenomics showed that 25 viral operational taxonomic units (vOTUs) in the MP microbial biofilm carried ARGs predominantly associated with MDR and MLS resistance, comprising a total of 22 subtypes. The relative abundance of ARGs in the digestive gland of *M. coruscus* after MP ingestion significantly decreased, except for MDR genes such as *efr*A and *pat*B, which increased [135].

PE and PET MPs aged for three months in a flow-through *Magallana angulata* oyster aquaculture farm harbored approximately 10^10^ copies/g of the 16S rRNA gene, and copy numbers of *intI*1, *sul*1, *sul*2, *tet*C, and the plasmid-encoded multi-resistance efflux pump *oqx*B at copy numbers of 10^6^/10^7^, 10^8^, 10^7^, 10^6^ and 10^5^ copies/g, respectively. The gene *tet*G was present at about 10^7^ copies/g on PET and the copy numbers of *intI*1, *sul*1, *erm*B, and the chloramphenicol exporter *fex*A differed significantly between the two plastic types. In the oysters exposed to PE for 14 days, the genes *oqx*B and *tet*A increased by approximately 8- and 12-fold, while in those exposed to PET, *oqx*B, *tet*G, and *intI*1 increased by 6-, 5- and 1-fold, respectively. The copy number of *sul*1 was significantly higher in the PET group compared to the PE group. In oyster excreta, all ARGs were enriched except for *tet*C, indicating that the excreta can transmit ARGs to other aquatic organisms, leading to their introduction into the food chain. The *intI*1 gene increased in oysters exposed to MPs and in their excreta, and in oysters it was positively correlated with *sul*2 and *tet*G, while in the excreta it was positively correlated with *sul*1 [136].

One study analyzed the effect of trophic transfer of MPs on ARG selection in aquatic animals by examining the effect of PP-MPs on the transfer of oxytetracycline from the shrimp *Neocaridina denticulata* to the crucian carp *Carassius carassius*. Shrimps were exposed to 200 µg/L oxytetracycline, a concentration within the range found in aquaculture environments, either alone or in combination with 100 µg/L PP-MPs, and then fed to carps under controlled conditions. After 14 days of exposure, oxytetracycline levels in the shrimp gut and in the carp liver and gut were significantly higher in the presence of the MPs/antibiotic combination compared to the antibiotic alone. Oxytetracycline induced an increase in Actinobacteria and Firmicutes in the shrimp, and Bacteroidetes in the carp, while its combination with MPs induced an increase in Actinobacteria in the shrimp and Firmicutes in the carp. Exposure to oxytetracycline and MPs from water increased the abundance of *Gemmobacter*, *Rhizobium*, and *Shinella*, and pathogenic bacteria in both animals compared to exposure to the antibiotic alone. Trophic uptake of oxytetracycline and MPs by the fish led to an increase in the genera *Gemmobacter*, *Pseudorhodobacter*, *Cetobacterium*, and *Acinetobacter*, and a greater decrease in *Akkermansia* and *Bacteroides* to a greater extent than when exposure occurred directly from water. MPs enhanced the increase of *tet*A, *cml*_e3, *sul*1, *tet*G, and *aph33i*B in the shrimp gut induced by oxytetracycline, while *tet*E, *tet*A, *sul*1, and *tet*M increased in the carp gut after exposure to MPs and oxytetracycline through water, whereas only *tet*E increased after exposure to oxytetracycline alone. This increase was further enhanced when the carp ingested the antibiotic by eating shrimp exposed to oxytetracycline, and even more so when the shrimp was also exposed to MPs. In this case, *tet*A and streptomycin ARGs increased, suggesting that MPs adsorbed oxytetracycline, enhancing its trophic transfer. Based on correlation networks, the ARGs *tet*A, *tet*G, *sul*1, *cat*b3, *cml*_e3, *aph*33iB, and *aph*6iD had a higher number of potential hosts in the shrimp intestine, while *tet*M, *tet*B, *tet*36, *tet*C, and *aac*6iB had a higher number of potential hosts in the intestines of crucian carp, including *Clostridium*, *unclassified_ f_* Clostridiaceae and *Bacteroides* [137].

**Figure 2:**
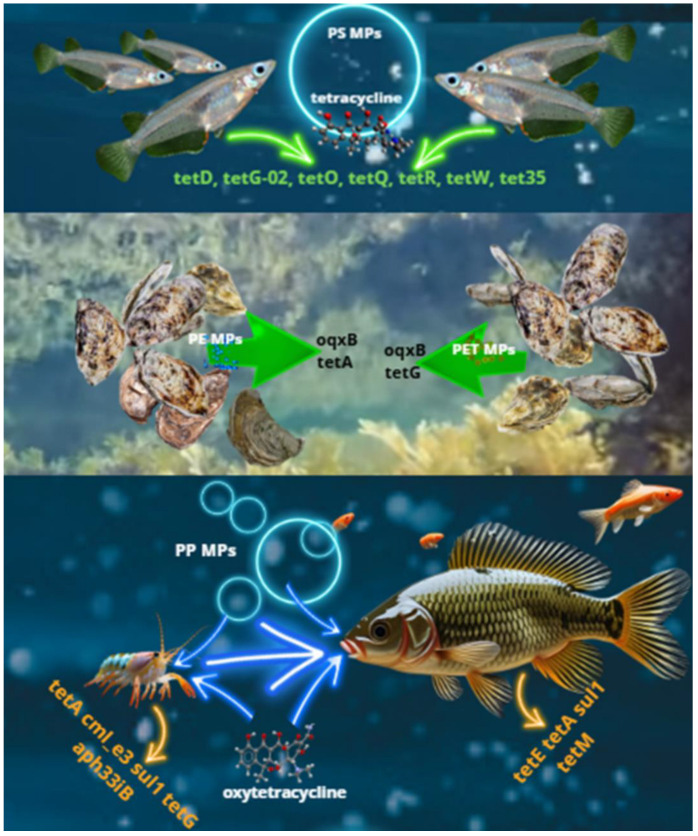
The demonstrated effects of exposure to MPs and antibiotics on the enrichment of ARGs in fishery products. PE (polyethilene), PET (polyethylene therephtalate), PP (polypropylene) [128,136,137].

According to the studies reviewed here, some connections were established between exposure to MPs, with or without associated antibiotics, and ARG selection in aquatic animals. The most notable are illustrated in Figure 2.

## 6. Discussion

This comprehensive review collected evidence from numerous recent studies showing that plastic waste of various sizes favors the selection and enrichment of ARB and ARGs in aquatic environments. This mainly occurs because MPs serve as a solid support for the formation of bacterial biofilms, which promote HGT events involving MGEs. The abundance of MGEs is often found to be positively correlated with that of ARGs and MDRGs and/or enriched in the plastisphere. Although some studies reported a lower abundance of ARGs in plastic biofilms than in water, positive correlations between ARGs, MGEs, and bacterial carriers were still found in the plastisphere, indicating an increased HGT risk in MPs. In particular, the occurrence of ARGs in MPs being lower than in water, as found in a few studies, contradicts the findings of other investigations. However, this inconsistency is most likely due to the use of targeted detection of relatively few ARGs by qPCR, which may have missed other ARGs present [29,40,44,45,46,47,49,50,60,64,67,69,73,74,76,77,78,82,83,84,89,90,96,97,98,99,103,104,105,106,107,108,117,118,120,122,124,136].

The number of studies addressing the MP route of AMR transmission increased annually, from 1 in 2018 and 2 in 2019 to 5 in 2020, 7 in 2021, 14 in both 2022 and 2023, and 24 in 2024. In the current year, 21 studies have been published, indicating growing scientific interest in the concern posed by plastic waste in the spread of AMR in aquatic environments, where fauna, including edible species, are directly exposed to these contaminants. Although only eight studies have analyzed the effect of exposure to MPs/NPs and antibiotics on the AMR profile of aquatic animals, ARG selection by MPs in the gut microbiota of fishes, crustaceans, and mollusks has been demonstrated [125,127,128,129,131,132,136,137]. Moreover, an increase in ARGs and MDRGs in aquatic organisms due to NPs has also been demonstrated in *D. rerio* [125]. Therefore, the widespread presence of ARGs and MDRGs in plastic debris in aquatic environments such as rivers, lakes, estuaries, mangroves, seas, coral reefs, and aquaculture farms indicates undesired effects occurring in aquatic animals and highlights the need to reduce their exposure to MPs to limit the risk of ARG introduction into the food chain [29,39,40,41,42,43,44,45,46,47,49,50,51,52,53,54,55,56,57,58,59,60,61,62,63,64,65,66,67,69,70,73,74,75,76,77,78,79,80,81,82,83,84,85,86,87,88,89,90,91,92,93,94,95,96,97,98,99,100,101,102,103,104,105,106,107,108,116,117,118,119,120,121,122,123,124,125,126,127,128,129,130,131,132,133,134,135,136,137].

ARG selection and HGT in the plastisphere occur in water bodies with varying degrees of salinity and have been reported in different countries, including those with extreme climates, demonstrating that plastic waste is a global hotspot for AMR spread [43,94]. Most in situ and ex situ studies on the presence of ARGs and MDRGs in the plastisphere in aquatic environments have been conducted in China, covering both water bodies and aquaculture farms, while relatively few studies have been conducted in Europe, four in Asian countries other than China, four in African countries, one in Antarctica, and one in America [29,40,42,43,44,45,46,47,48,49,50,51,53,54,56,57,59,61,64,65,66,67,68,69,73,75,76,77,78,79,81,82,83,85,86,88,89,90,91,92,93,94,96,97,98,99,100,101,102,103,104,105,107,108,114,116,117,118,120,125]. This uneven distribution of studies indicates that a significant portion of the globe remains unmonitored, and the issue of ARG enrichment by plastic waste in water bodies is still not addressed compared to other measures adopted to contain the AMR pandemic [138].

Nevertheless, the presence of MPs and the development of the plastisphere in rivers, lakes, and seas, which serve as final recipients of wastewater, are of great concern. MPs, due to their low density, remain suspended in water and are transferred from WWTPs, as well as agricultural and industrial runoff, to water bodies along with their microbial load [59,69,72]. Several studies have clearly demonstrated the significant role of WWTPs in reshaping the composition of microbial consortia and ARG assembly in MP biofilms [45,72,73,76,78,79,81,88]. It has also been reported that MPs can be covered by a biofilm even in tap water, where they may promote ARG enrichment and spread [76]. Furthermore, plastic aging due to exposure to light and fragmentation increases the rate of bacterial adhesion and ARG enrichment [15,126,134].

Some specific ARGs and MDRGs were detected in multiple studies, and most of these were found in all aquatic environments considered in this review: freshwater, seawater, estuarine sites, and aquaculture farms. Among those detected in all four environments were *sul*1, reported in 31 instances, s*ul*2 in 21 instances, *tet*A in 17 instances, *bla*_TEM_, *erm*B and *tet*W in nine instances and *erm*F in eight instances. MDRGs were detected in 25 studies, and in most cases, more than one representative was found in the same environment. Therefore, it can be stated that MDR was more widespread than resistance to single antibiotic classes. ARGs not found in all environments but detected in both freshwater and seawater, indicating little influence from salinity, included *bac*A, reported in 14 studies, *tet*C in 11 studies, *qnr*S in eight studies, *tol*C in six studies, and *qnr*A in four studies. Other genes were detected in freshwater and aquaculture farms, with some also present in estuarine environments. These, ordered by decreasing number of reports from eight to five, were *tet*G, *dfr*A1, *ere*A, *tet*B, and *tet*M. Other genes present in both freshwater and seawater, and some also detected in estuarine waters but not in aquaculture farms, included the efflux pump gene *acr*B in seven studies, *mac*AB, *mex*F, and *ceo*B in four studies and the MDR transporters *msb*A, *ros*A, and *fos*X in three studies [29,38,39,40,41,42,43,44,45,47,48,49,50,51,52,53,54,56,58,59,60,61,64,66,67,69,71,74,75,76,78,79,80,81,83,84,86,87,88,89,90,92,93,96,97,98,99,100,101,102,103,104,105,106,115,117,118,119,120,122,123,124,125,126,128,129,130,134,136,137].

As an MGE indicator, the integrase gene *intI*1 was most frequently detected in all environments, as reported by 29 studies. The next most frequently detected MGE indicator in both freshwater and seawater was *tnp*A, with its variants found only in freshwater [29,39,40,44,45,47,48,50,51,53,54,57,58,59,60,64,66,71,73,74,76,78,79,83,89,96,97,99,103,105,117,118,119,120,122,124,136].

Notably, various studies reported the detection of genes for ESBLs ARGs of major global burden [139] in plastic biofilms [29,41,47,48,51,58,59,61,77,78,83,84,86,88,93,101,104,114,118,120], and the ability of bacteria isolated from the plastisphere to disseminate ESBL via conjugation was demonstrated [56,77]. Moreover, the species *S. aureus*, including the MRSA ARB of global concern, was detected in the plastisphere from a WWTP effluent and from mangroves, while the associated ARGs were detected on PBAT in a tilapia farm [81,98,124].

Compared to the systematic review by Zhu et al. [38], which considered only metagenome datasets, predominantly from PVC, this literature survey also included results obtained using other molecular techniques and experimental approaches. The results agreed on the higher abundance of ARGs in BDPs compared to NBPs, but not on the ranking of NBPs based on ARG abundance. The findings on the frequency of ARG types detected were consistent in showing the predominance of MDRGs, sulfonamide, and tetracycline ARGs. Aminoglycoside ARGs, identified as the most abundant after tetracycline ARGs by Zhu et al. [38] and predominant in the study by Yang et al. [84], were reported by 23 studies discussed here and were represented by a diverse group of genes [29,41,60,66,67,69,78,81,83,84,85,104,105,114,137].

According to several studies, the type of plastic polymer influences transformation and conjugation frequency, as well as the selection or abundance of different bacterial groups, ARG classes, and MGEs [22,27,28,29,33,38,39,42,43,45,48,49,50,52,53,54,56,57,58,59,60,64,67,69,75,78,79,89,94,96,98,100,103,106,107,120,124,136]. PP was identified in various studies as the polymer with the highest tendency to promote biofilm formation. In particular, it showed higher biomass abundance compared to PE and PS in a study of mixed biofilm formation and HGT between *B. subtilis* and *A. baylyi* [22]. Moreover, the MCDM method VIKOR indicated PP as the material with the highest risk of HGT, as biomolecule interaction prediction showed that PP stress strengthened the binding of active codons by the relaxase and VirB5 protein involved in conjugation [28]. The ARG assembly in PP in freshwater was found to be dominated by stochastic processes [57], and ciprofloxacin-resistant bacteria were more abundant on PP both upstream and downstream from a WWTP in the Mondego River (Portugal) [77,78,96]. In addition, PP, followed by PE, was the polymer harboring more viral ARGs and was positively correlated with the highly positive z potential that favors the binding of negatively charged DNA [107]. Network correlation showed more numerous interactions between viruses and bacteria on PP [107], and PP also exhibited the highest antibiotic adsorption rates in mariculture [116]. Based on these findings, it appears that PP binds microorganisms, environmental DNA, and antibiotics more efficiently than other NBPs, thus representing a high-risk material for ARG HGT and spread.

Some studies have identified PVC as the polymer with the highest tendency to release toxic components. In particular, bacterial diversity was lower than on other polymers deployed in the Henares River (Spain), possibly due to the release of inhibitory substances [79]. Similarly, in mariculture sediments exposed to PVC, a significantly lower bacterial diversity was observed. Moreover, it was shown that PVC, more than other polymers, promoted microbiota assembly driven by deterministic processes, which could depend on selective pressure possibly exerted by the release of Zn, Cu, phthalates, and BPA [29]. On the other hand, it was demonstrated that PVC leachate promoted an increase in ARGs [87] and HGT, with a synergistic effect with PVC particles. In a study using green fluorescing transconjugants, their appearance was observed on the surface of PVC earlier than on PS, and with a higher ratio of transconjugants to donors. The conjugal transfer on PVC was possibly favored by oxidative stress, as inferred from the higher abundance of ROS in bacteria adhering to PVC [29].

BDPs, among which PLA is the most widely used and whose quantity is increasing globally, also cause ARB and ARG enrichment, favored by their high tendency to fragment, forming small particles. Various studies have shown that BDPs can pose a higher risk than NBPs in increasing and spreading ARGs [49,50,57,59,64,71]. Moreover, a synergistic effect with the toxic plasticizer DBP has been demonstrated for PLA [32].

Some of the in situ studies discussed here established vague relationships between bacterial hosts and ARGs or MDRGs, while others associated bacterial groups, and in some cases specific bacterial genera or species, with the detected ARGs through network correlation analysis or experimental methods (Table 1, Table 2 and Table 3). Moreover, predictive models were developed to associate specific ARGs with their respective bacterial hosts [38]. Precisely establishing these connections could help limit the probability of ARG HGT in the plastisphere by reducing or preventing contamination by particular microbial groups capable of transmitting or acquiring AMR genes. Indeed, the sources of these microorganisms could be identified and more strictly and efficiently controlled. Since various studies have demonstrated the contribution of WWTP effluents and urbanization to ARG enrichment in the plastisphere, improving the water purification process is pivotal in mitigating the related risks. Therefore, further efforts should be dedicated to studying the effects of wastewater treatments in reducing the persistence of specific ARB [69]. In particular, action is required to improve the effectiveness of constructed wetlands in eliminating ARB, ARGs, and MPs implicated in their selection [140].

Another source of ARG-harboring MPs is aquaculture plants, and the task of simultaneously eliminating MPs and ARB/ARGs from aquaculture effluents is challenging. Various chemical, physical, and biological technologies, as well as their combinations, have been tested but still require optimization. Feasible treatments include physical removal of MPs by coagulation and filtration using materials such as polyaluminium chloride, magnetic biochar modified with quaternary phosphonium salt, and reverse osmosis, as well as ARG/MGE elimination through chemical oxidation by Fenton or H_2_O_2_ oxidation, ozone or polysulphate treatment, or anaerobic combined oxidation. Promising technologies include advanced oxidation processes combining UV light and H_2_O_2_, or high-frequency electromagnetic fields with low-dose chlorine [141].

A biological purification method utilizes the symbiosis between bacteria and algae in constructed wetlands (CWs), which can remove both MPs and ARGs. In this consortium, algae use CO_2_ to produce organic matter, which bacteria can transform into inorganic matter used by the microalgae. The algae generate large amounts of oxygen through photosynthesis, facilitating the degradation activities of aerobic bacteria. The use of suspended algae such as *Scenedesmus* and *Chlorella* has been shown to rapidly remove high levels of MPs and ARGs from aquaculture water. However, research on the removal of MPs by bacteria and algae consortia remains limited. Another promising biological strategy involves microbial fuel cells (MFCs), in which electricity-producing microorganisms and antibiotic-degrading bacteria form a biofilm on the anode. In this system, antibiotics are degraded while electricity is generated, enabling aquaculture wastewater decontamination along with bioelectric energy production. The efficiency of these decontamination processes is strongly influenced by environmental factors, so further optimization is needed [141].

An aspect that requires further investigation is the role of MPs in increasing ARG content in natural matrices such as water and sediment. A recent study using long-read metagenome analysis of river water and sediment microcosms showed that the presence of MPs increased the diversity of ARGs in the surrounding matrices. In the presence of MPs, after 7 and 14 days, a higher number of ARGs were detected in the sediment, including *bla*_TEM-116_, the oxytetracycline resistance gene *otr*C, the MLS efflux pump gene *ole*C, and *oqx*B at day 7 and *dfr*B3, the tetracycline efflux pump *tcr*3, *ole*C, and *otr*A at day 14. Moreover, MPs led to an increase in *Aeromonas* spp., specifically *A. salmonicida*, *A. hydrophila*, and *A. veronii*. In water samples, changes in the relative abundance of Proteobacteria classes were observed, and ARGs *dfr*B3, *mph*E, *ole*C, *tcr*3, and *otr*A were detected at day 7, and *qep*A4, *oqx*B, and *otr*A at day 14, while in the absence of MPs only the *vat*F gene responsible for resistance to dalfopristin, pristinamycin, and virginiamycin was detected [142].

## 7. Conclusions

Most of the studies reviewed reported the selection and enrichment of ARBs and ARGs in water bodies on plastic waste. All studies agreed that plastic biofilms promote HGT, leading to enrichment of MGEs and their positive correlations with ARGs and/or their bacterial hosts. As a result, aquatic animals directly exposed to plastic particles such as MPs and NPs not only suffer from their toxicity but also experience gut microbiota disruption, with selection for ARG carriers. These carriers can be propagated through trophic transfer and may pose risks to human health if present in edible species, especially those consumed raw. New studies linking plastic contaminants with ARB and ARG selection should aim to clarify the factors that favor ARB establishment and ARG propagation in aquatic environments to develop strategies that mitigate the associated risks. More studies evaluating the effects of MPs covered by a plastisphere on ARG selection in aquatic organisms and their transmission through the food chain are needed to better estimate the scale of the problem.

## Figures and Tables

**Table 1:** ARGs and the most probable or confirmed bacterial hosts identified in plastic polymers in freshwater, with polymer types and study sites.

Plastic Polymer	Site	Bacterial Host	ARG	Reference
PBAT, PET	microcosm	*Afipia*, Rhizobiaceae	*qnr*S	[39]
		*Bacillus* spp.	*sul*2, *bla*Q	
		*Gemmobacter*, *Conexibacter*, *Lamia*	*tet*A, *tet*C, *tet*X, *ere*B	
PE, PP, PBD	Ganjiang river (China)	*Streptococcus mitis*	*erm*F, *erm*B	[44]
Mixed polymers	Huangpu River (China)	*Afipia* spp.	*aac*(2′)-I, *arr*, *cat*, *mex*I, *bla*_TEM-1_, *tet*V	[45]
PVC, PLA	freshwater microcosm with tetracycline	*Pseudomonas* spp., Flavobacteriaceae, Actinobacteria	*tet*A, *tet*C, *tet*M, and *tet*X	[21]
PET, PVC	freshwater microcosm with tetracycline	Genera *Pseudomonas*, *Solobacterium*, *Achromobacter*, *Aeromonas*, *Beggiatoa*, *Propionivibrio*, *Paludibacter*	*tet*A, *tet*C, *sul*1, *tet*O	[48]
Mixed polymers	Haihe River (China)	*Enterobacter cloacae*	*tet*G	[49]
APSs	Bracciano Lake (Italy)	*Morganella morganii*	*tet*C, *sul*1, *sul*3, *cml*A1, *cmx*A, *bla*_CTX-M-01_, *bla*_CTX-M-02_	[51]
Mixed polymers	Mondego river (Portugal)	*E. coli*, *Citrobacter* spp., *Enterobacter* spp., *Klebsiella pneumonia*, *Shigella* spp.	*aac*A4-*cr*, *qnr*S, *qnr*B, *qnr*VC, *bla*_CTX-M_, *bla*_CTX-M-15_, *bla*_CTX-M-32_, *bla*_CTX-M-55_	[77]
PE, PP	Poyang Lake (China)	*A. veronii*	*bla*_OXA-12_, *cph*A6, *cph*A8, *cph*A3	[23]
		*Arthrobacter* spp.	*mds*B, *nov*A, *rph*A, *emr*K	
BDPs	Lakes in Wuhan (China)	*Riemerella anatipestifer*	*nov*A, *mac*B, *mds*B, *sav*1866, *tae*A, *arn*C, *mfd*	[57]
		*Vibrio campbellii*	*nov*A, *bla*_CRP_, *tae*A, *pmr*E, *tet*4	
		*V. cholerae*	*pbp1a*, *mur*A, *efr*A, *mtr*A, *tet*PB	
Mixed polymers	Lake water	*Lysinibacillus* spp., *Exiguobacterium acetylicum*, *K. pneumoniae*, *K. oxytoca*, *K. michiganensis*	*bla*_TEM_ (shared by all bacteria), *bla*_SHV_, *ade*A, *tet*A, *acr*B, *sul*1, *mec*A, *tet*W, *acr*F, *cmx*A, *sul*2	[58]
Mixed polymers	Red River (Vietnam)	*Aeromonas* spp.	*bla*_TEM_, *bla*_SHV_, *bla*_CTXM_	[61]
PHA	Central Lake (China)	Proteobacteria	*bac*A, *mec*A, *qac*A, *tol*C, *dfr*A1, *mph*A, *mph*B	[64]
Mixed polymers	treated wastewater microcosm	*Stenotrophomonas maltophilia*	*sul*1	[80]
PET, PLA	Treated wastewater	*Candidatus Microthrix*	*tet*A48, *kdp*E, *rpo*B2	[69]
		*Acinetobacter* spp.	44 ARGs including *aad*A, *ksg*A, *aph*(3′)-I	
Mixed polymers	Jiuxiang River and Taihu Lake (China)	*Variovorax*, *Rubrivivax*, *Thauera*	ARGs for tetracycline	[71]
		*Herbaspyrillum*, *Limnohabitans*	ARGs for MLS, elfamycin and tetracyclines	
Not specified	Urban lake in Chengdu (China)	Gammaproteobacteria, Pseudomonadota, Acidobacteriota, Actinomycetota	*bac*A, *sul*1	[66]
	Urban river trait Chengdu (China)	Deltaproteobacteria, Desulfobacterales	*sul*1, *sul*2, *acr*B, *ant*(2″)-Ia	
	Rural lake Chengdu (China)	Gammaproteobacteria	*sul*1, *sul*2, *sme*E	
Not specified	Yangtze river (China)	genera *CAMDGX01*, *PHCI01*, *Shewanella*	*bac*A, *nov*A, *mex*F, *mcr*-4,3, *bla*_OXA-541_	[83]
		genera *Pseudomonas*, *Serratia*	*bac*A, *arn*A, *mex*B, *mux*B, *aac*(6′)-Ic, *acr*D	

APSs, Artificial Plastic Substrates; PBAT, poly butyleneadipate-co-terephthalate; PBD, polybutadiene; PE, polyethylene; PET, polyethylene therephtalate; PP, polypropylene; BDPs, biodegradable plastics; PHA, polyhydroxyalkanoate; PLA, polylactic acid; PVC, polyvinylchloride.

**Table 2:** ARGs and their most probable or confirmed bacterial hosts identified in plastic polymers from seawater, with polymer types and study sites.

Plastic Polymer	Site	Bacterial Host	ARG	Reference
Mixed polymers	Vestland county, Norway	*A. salmonicida*, *M. morganii*, *A. beijerinckii*	class C beta-lactamases, *cat*B	[85]
		*A. salmonicida*	*cph*A	
		*Aeromonas* spp.	*qnr*A	
		*A. beijerinckii*	class A beta-lactamase, aminoglycoside acetyltransferase, *cat*	
PBAT, PET	microcosm	*Labrenzia*, Vicinamibacteraceae	*tet*A, *bla*Q	[39]
PBAT, PET	microcosm	*Labrenzia*, Vicinamibacteraceae, Acidobacteriota, *Coxiella*, *Croceibacter*, *Tumebacillus*	*qnr*B	
Not specified among PE, PP, PS and PVC	Busan City (South Korea)	*Coxiella* spp.	*tet*A	[89]
		*Pseudahrensia* spp.	*tet*Q	
		Genera *Fuerstia*, *Methylotenera*, *Halioglobus*, *Ahrensia*, *Rubritalea*, *Algibacter*	*erm*B	
Mixed polymers	Tyrrhenian Sea (Italy)	Rhizobiales	*bac*A	[90]
		*Photobacterium* spp.	*tol*C, *acr*B, *tet*34	
		*Pleurocapsa* spp.	*vat*F	
PET	Coral reef, Hainan (China)	Nine bacterial genera	*sul*2	[92]
		*Vibrio* spp.	*sul*1	
Mixed polymers	Monastir, Mahdia (Tunisia)	*Shewanella arctica*	*bla*_TEM_	[93]

PBAT, poly butyleneadipate-co-terephthalate; PE, polyethylene; PET, polyethylene therephtalate; PP, polypropylene; PS, polystyrene; PVC, polyvinyl chloride.

**Table 3:** ARGs and their respective bacterial hosts identified in plastic polymers from estuarine and brackish waters, along with polymer types and study sites.

Plastic Polymer	Site	Bacterial Host	ARG	Reference
Mixed polymers	Laguna Madre (Mexico)	*Bacillus cereus*	ARGs for 10 antibiotic classes	[100]
		*B. thuringiensis*	ARGs for 20 antibiotic classes	
Mixed polymers	Yangtze, Sheyang, Guanhe and Xinyi rivers (China)	Proteobacteria	*bla*_NDM-1_, *tet*A, *tet*X, *sul*1, *sul*2	[101]
		Bacteroidota	*bla*_TEM_, *erm*B, *ere*A, *tet*X, *sul*1	
Mixed polymers	Tokyo, Saitama, Chiba (Japan)	Genera *Citrobacter*, *Aeromonas*, *Sulfitobacter*, *Lacinutrix*	*bac*A, *acr*AB-*Tol*C, mutated *mar*R, *acr*AB inducer *mar*A, *van*G, *ade*F, *qac*G, *van*H	[104]
		*K. pneumoniae*	*kpn*F	

## Data Availability

Not applicable.

## Abbreviations

ACE	Abundance-based Coverage Estimator
ADI	Acceptable daily intake
AHL	Acyl-homoserine lactones
AMR	Antimicrobial resistance
APS	Artificial plastic substrate
ARB	Antibiotic-resistant bacteria
ARG	Antibiotic resistance gene
ARISA	Automated ribosomal intergenic spacer analysis
BDP	Biodegradable plastic
BPA	Bisphenol A
CF	Cellophane
CR	Cancer risk
DBP	Dibutyl phthalate
DCFH-DA	2′,7′-Dichlorofluorescein diacetate
DEGM	Dietary exposure dose for the human gut microbiome
DEHP	(2-ethylhexyl) Phthalate
EDI	Estimated daily intake
EPS	Extracellular polysaccharides
ESBL	Extended spectrum beta-lactamase
ESKAPE	*E. faecium*, *S. aureus*, *K. pneumoniae*, *A. baumannii*, *P. aeruginosa*, *Enterobacter* spp.
FTIR	Fourier transform infrared
GBDT	Gradient boosting decision tree
GHRI	Genotypic health risk index
HDPE	High-density PE
HGT	Horizontal gene transfer
HQ	Human health risk quotient
HT-qPCR	High-throughput qPCR
HTS	High-throughput sequencing
ICE	Integrative conjugative element
IHRI	Integrated health risk index
LDPE	Low-density PE
LGBM	Light Gradient Boosting machine
LSCM	Laser scanning confocal microscopy
MAG	Metagenome assembled genome
MALDI	Matrix-assisted laser desorption ionization
MALDI-TOF	Matrix-Assisted Laser Desorption Ionization Time-of-Flight
MAR	Multiple antibiotic resistance
MARB	Multiresistant bacteria
MCDM	Multiple criteria decision making
MD	Molecular dynamics
MDR	Multidrug resistance
MDRG	Multidrug resistance gene
MGE	Mobile genetic element
MIC	Minimum inhibitory concentration
ML	Machine learning
MLP	Multilayer perceptron
MLR	Multiple linear regression
MLS	Macrolide–lincosamide–streptogramin
MP	Microplastic
MRSA	Methicillin-resistant *S. aureus*
MSC	Minimal Selective Concentration
NBP	Non-biodegradable plastic
NCM	Neutral community model
NP	Nanoplastic
OTU	Operational taxonomic unit
PA	Polyamide
PAH	Polycyclic aromatic hydrocarbon
PBAT	Poly-butyleneadipate-co-terephthalate
PBD	Polybutadiene
PBS	Polybutylene succinate
PCB	Polychlorinated biphenyl
PCL	Polycaprolactone
PCoA	Principal coordinate analysis
PDMS	Polydimethylsiloxane
PE	Polyethylene
PET	Polyethylene therephthalate
PF	Phenol formaldehyde
PF	PE-fiber
PFP	Pentafluorophenyl acrylate
PFP	PE-fiber-PE
PHA	Polyhydroxyalkanoate
PHB	Polyhydroxybutyrate
PLA	Poly lactic acid
PLC	Polyisoprene chlorinated
PMMA	polymethyl methacrylate
PP	Polypropylene
ppLFER	Poly-parameter Linear Free-Energy Relationships
PPR	Projection pursuit regression
PS	Polystyrene
PTDL	Polytridecanolactone
PU	Polyurethane
PVC	Polyvinyl chloride
QS	Quorum sensing
QSPR	Quantitative Structure Property Relationship
RF	Random forest
RM	Random forest
RMSD	Root-mean square deviation
RMSE	Root Mean Square Error
ROS	Reactive oxygen species
SAN	Styrene acrylonitrile resin
SC	Specific conductivity
SEM	Scanning electron microscopy
SHAP	SHapley Additive exPlanation
SRA	Sequence Read Archive
SSA	Specific surface area
T4SS	Type IV secretion system
TAOC	Total antioxidant capacity
TDS	Total dissolved solids
THQ	Target hazard quotients
TOC	Total organic carbon
TWP	Tire wear particles
vOTU	Viral operational taxonomic unit
WGS	Whole-genome sequencing
W-LDPE	Waste LDPE
WWTP	Wastewater treatment plant
XGB	eXtreme Gradient Boosting

## References

[1] Global Burden of Disease 2021 Antimicrobial Resistance Collaborators (2024). Global Burden of Bacterial Antimicrobial Resistance 1990–2021: A Systematic Analysis with Forecasts to 2050. Lancet.

[2] Food and Agriculture Organization of the United Nations Assessment of Agricultural Plastics and Their Sustainability a Call for Action. Rome 2021. https://openknowledge.fao.org/items/94eb5786-232a-496f-8fcf-215a59ebb4e3.

[3] Khare T., Mathur V., Kumar V. (2024). Agro-Ecological Microplastics Enriching the Antibiotic Resistance in Aquatic Environment. Curr. Opin. Environ. Sci. Health.

[4] Gigault J., Halle A.T., Baudrimont M., Pascal P.-Y., Gauffre F., Phi T.-L., El Hadri H., Grassl B., Reynaud S. (2018). Current Opinion: What Is a Nanoplastic?. Environ. Pollut..

[5] Alberghini L., Truant A., Santonicola S., Colavita G., Giaccone V. (2022). Microplastics in Fish and Fishery Products and Risks for Human Health: A Review. Int. J. Environ. Res. Public Health.

[6] Santonicola S., Volgare M., Rossi F., Castaldo R., Cocca M., Colavita G. (2024). Detection of Fibrous Microplastics and Natural Microfibers in Fish Species (*Engraulis encrasicolus*, *Mullus barbatus* and *Merluccius merluccius*) for Human Consumption from the Tyrrhenian Sea. Chemosphere.

[7] Volgare M., Santonicola S., Cocca M., Avolio R., Castaldo R., Errico M.E., Gentile G., Raimo G., Gasperi M., Colavita G. (2022). A Versatile Approach to Evaluate the Occurrence of Microfibers in Mussels *Mytilus galloprovincialis*. Sci. Rep..

[8] Santonicola S., Volgare M., Schiano M.E., Cocca M., Colavita G. (2024). A Study on Textile Microfiber Contamination in the Gastrointestinal Tracts of *Merluccius merluccius* Samples from the Tyrrhenian Sea. Ital. J. Food Saf..

[9] Li Y., Ling W., Hou C., Yang J., Xing Y., Lu Q., Wu T., Gao Z. (2025). Global Distribution Characteristics and Ecological Risk Assessment of Microplastics in Aquatic Organisms Based on Meta-Analysis. J. Hazard. Mater..

[10] Li J., Zhang K., Zhang H. (2018). Adsorption of Antibiotics on Microplastics. Environ. Pollut..

[11] Zettler E.R., Mincer T.J., Amaral-Zettler L.A. (2013). Life in the “Plastisphere”: Microbial Communities on Plastic Marine Debris. Environ. Sci. Technol..

[12] Galloway T.S., Cole M., Lewis C. (2017). Interactions of Microplastic Debris throughout the Marine Ecosystem. Nat. Ecol. Evol..

[13] Zhang G., Chen J., Li W. (2022). Conjugative Antibiotic-Resistant Plasmids Promote Bacterial Colonization of Microplastics in Water Environments. J. Hazard. Mater..

[14] Zhou R., Huang X., Xie Z., Ding Z., Wei H., Jin Q. (2024). A Review Focusing on Mechanisms and Ecological Risks of Enrichment and Propagation of Antibiotic Resistance Genes and Mobile Genetic Elements by Microplastic Biofilms. Environ. Res..

[15] Tian Y., Zhu J., Ying C., Luo H., Zhang S., Zhang L., Li R., Li J. (2023). Photoaging Processes of Polyvinyl Chloride Microplastics Enhance the Adsorption of Tetracycline and Facilitate the Formation of Antibiotic Resistance. Chemosphere.

[16] Oberbeckmann S., Kreikemeyer B., Labrenz M. (2018). Environmental Factors Support the Formation of Specific Bacterial Assemblages on Microplastics. Front. Microbiol..

[17] Stapleton M.J., Ansari A.J., Hai F.I. (2023). Antibiotic Sorption onto Microplastics in Water: A Critical Review of the Factors, Mechanisms and Implications. Water Res..

[18] Gao Z., Kong L., Han D., Kuang M., Li L., Song X., Li N., Shi Q., Qin X., Wu Y. (2025). Deciphering the Adsorption Mechanisms between Microplastics and Antibiotics: A Tree-Based Stacking Machine Learning Approach. J. Clean. Prod..

[19] Abdollahi S., Raissi H., Farzad F. (2025). The role of microplastics as vectors of antibiotic contaminants via a molecular simulation approach. Sci. Rep..

[20] Müller N.D., Kirtane A., Schefer R.B., Mitrano D.M. (2024). EDNA Adsorption onto Microplastics: Impacts of Water Chemistry and Polymer Physiochemical Properties. Environ. Sci. Technol..

[21] Wang X., Li H., Chen Y., Meng X., Dieketseng M.Y., Wang X., Yan S., Wang B., Zhou L., Zheng G. (2022). A Neglected Risk of Nanoplastics as Revealed by the Promoted Transformation of Plasmid-Borne Ampicillin Resistance Gene by *Escherichia coli*. Environ. Microbiol..

[22] Wang H., Xu K., Wang J., Feng C., Chen Y., Shi J., Ding Y., Deng C., Liu X. (2023). Microplastic Biofilm: An Important Microniche That May Accelerate the Spread of Antibiotic Resistance Genes via Natural Transformation. J. Hazard. Mater..

[23] Liu X., Huang C., Yu H., Yang Y., Ma L., Zhao B., Zhong T., Zhang L., Peng W., Gong W. (2024). Quorum Sensing Regulating the Heterogeneous Transformation of Antibiotic Resistance Genes in Microplastic Biofilms. J. Environ. Chem. Eng..

[24] Arias-Andres M., Klümper U., Rojas-Jimenez K., Grossart H.-P. (2018). Microplastic Pollution Increases Gene Exchange in Aquatic Ecosystems. Environ. Pollut..

[25] Jaffer Y.D., Abdolahpur Monikh F., Uli K., Grossart H.-P. (2024). Tire Wear Particles Enhance Horizontal Gene Transfer of Antibiotic Resistance Genes in Aquatic Ecosystems. Environ. Res..

[26] Luo Y., Xu T., Li B., Liu F., Wu B., Dobson P.S., Yin H., Chen Z., Qiu Y., Huang X. (2024). The Effects of Small Plastic Particles on Antibiotic Resistance Gene Transfer Revealed by Single Cell and Community Level Analysis. J. Hazard. Mater..

[27] Zhou Y., Zhang G., Zhang D., Zhu N., Bo J., Meng X., Chen Y., Qin Y., Liu H., Li W. (2024). Microplastic Biofilms Promote the Horizontal Transfer of Antibiotic Resistance Genes in Estuarine Environments. Mar. Environ. Res..

[28] Xiao B., Pu Q., Ding G., Wang Z., Li Y., Hou J. (2025). Synergistic Effect of Horizontal Transfer of Antibiotic Resistance Genes between Bacteria Exposed to Microplastics and per/Polyfluoroalkyl Substances: An Explanation from Theoretical Methods. J. Hazard. Mater..

[29] Liu Y., Liu L., Wang X., Shao M., Wei Z., Wang L., Li B., Li C., Luo X., Li F. (2025). Microplastics Enhance the Prevalence of Antibiotic Resistance Genes in Mariculture Sediments by Enriching Host Bacteria and Promoting Horizontal Gene Transfer. Eco-Environ. Health.

[30] Aßhauer K.P., Wemheuer B., Daniel R., Meinicke P. (2015). Tax4Fun: Predicting Functional Profiles from Metagenomic 16S rRNA Data. Bioinformatics.

[31] Zhao W., Sachsenmeier K., Zhang L., Sult E., Hollingsworth R.E., Yang H. (2014). A New Bliss Independence Model to Analyze Drug Combination Data. J. Biomol. Screen..

[32] Jiang Z., Zeng J., Wang X., Yu H., Yue L., Wang C., Chen F., Wang Z. (2025). Biodegradable Microplastics and Dissemination of Antibiotic Resistance Genes: An Undeniable Risk Associated with Plastic Additives. Environ. Pollut..

[33] Liu X., Wang X., Wang R., Guo S., Ahmad S., Song Y., Gao P., Chen J., Liu C., Ding N. (2023). Effects Comparison between the Secondary Nanoplastics Released from Biodegradable and Conventional Plastics on the Transfer of Antibiotic Resistance Genes between Bacteria. Environ. Pollut..

[34] Li Y., Liu X., Guo S., Wang L., Tang J. (2025). The Combination of Polystyrene Microplastics and Di (2-Ethylhexyl) Phthalate Promotes the Conjugative Transfer of Antibiotic Resistance Genes between Bacteria. Ecotoxicol. Environ. Saf..

[35] Gross N., Muhvich J., Ching C., Gomez B., Horvath E., Nahum Y., Zaman M.H. (2025). Effects of Microplastic Concentration, Composition, and Size on *Escherichia coli* Biofilm-Associated Antimicrobial Resistance. Appl. Environ. Microbiol..

[36] Shen H., Yang M., Yin K., Wang J., Tang L., Lei B., Yang L., Kang A., Sun H. (2023). Size- and Surface Charge-Dependent Hormetic Effects of Microplastics on Bacterial Resistance and Their Interactive Effects with Quinolone Antibiotic. Sci. Total Environ..

[37] Shruti V.C., Kutralam-Muniasamy G., Pérez-Guevara F. (2024). Diagnostic Toolbox for Plastisphere Studies: A Review. Trends Analyt. Chem..

[38] Zhu T., Li S., Tao C., Chen W., Chen M., Zong Z., Wang Y., Li Y., Yan B. (2024). Understanding the Mechanism of Microplastic-Associated Antibiotic Resistance Genes in Aquatic Ecosystems: Insights from Metagenomic Analyses and Machine Learning. Water Res..

[39] Zhou Q., Zhang J., Fang Q., Zhang M., Wang X., Zhang D., Pan X. (2023). Microplastic Biodegradability Dependent Responses of Plastisphere Antibiotic Resistance to Simulated Freshwater-Seawater Shift in Onshore Marine Aquaculture Zones. Environ. Pollut..

[40] Wang J., Qin X., Guo J., Jia W., Wang Q., Zhang M., Huang Y. (2020). Evidence of Selective Enrichment of Bacterial Assemblages and Antibiotic Resistant Genes by Microplastics in Urban Rivers. Water Res..

[41] Yang K., Chen Q.-L., Chen M.-L., Li H.-Z., Liao H., Pu Q., Zhu Y.-G., Cui L. (2020). Temporal Dynamics of Antibiotic Resistome in the Plastisphere during Microbial Colonization. Environ. Sci. Technol..

[42] Mughini-Gras L., van der Plaats R.Q.J., van der Wielen P.W.J.J., Bauerlein P.S., de Roda Husman A.M. (2021). Riverine Microplastic and Microbial Community Compositions: A Field Study in the Netherlands. Water Res..

[43] González-Pleiter M., Velázquez D., Casero M.C., Tytgat B., Verleyen E., Leganés F., Rosal R., Quesada A., Fernández-Piñas F. (2021). Microbial Colonizers of Microplastics in an Arctic Freshwater Lake. Sci. Total Environ..

[44] Hu H., Jin D., Yang Y., Zhang J., Ma C., Qiu Z. (2021). Distinct Profile of Bacterial Community and Antibiotic Resistance Genes on Microplastics in Ganjiang River at the Watershed Level. Environ. Res..

[45] Li R., Zhu L., Yang K., Li H., Zhu Y.-G., Cui L. (2021). Impact of Urbanization on Antibiotic Resistome in Different Microplastics: Evidence from a Large-Scale Whole River Analysis. Environ. Sci. Technol..

[46] Li H., Luo Q., Zhao S., Zhao P., Yang X., Huang Q., Su J. (2022). Watershed Urbanization Enhances the Enrichment of Pathogenic Bacteria and Antibiotic Resistance Genes on Microplastics in the Water Environment. Environ. Pollut..

[47] Xu C., Lu J., Shen C., Wang J., Li F. (2022). Deciphering the Mechanisms Shaping the Plastisphere Antibiotic Resistome on Riverine Microplastics. Water Res..

[48] Zhang S., Liu X., Qiu P., Chen B., Xu C., Dong W., Liu T. (2022). Microplastics Can Selectively Enrich Intracellular and Extracellular Antibiotic Resistant Genes and Shape Different Microbial Communities in Aquatic Systems. Sci. Total Environ..

[49] Wu X., Liu Z., Li M., Bartlam M., Wang Y. (2022). Integrated Metagenomic and Metatranscriptomic Analysis Reveals Actively Expressed Antibiotic Resistomes in the Plastisphere. J. Hazard. Mater..

[50] Zhou Q., Zhang J., Zhang M., Wang X., Zhang D., Pan X. (2022). Persistent versus Transient, and Conventional Plastic versus Biodegradable Plastic?—Two Key Questions about Microplastic-Water Exchange of Antibiotic Resistance Genes. Water Res..

[51] Ferheen I., Spurio R., Mancini L., Marcheggiani S. (2023). Detection of *Morganella morganii* Bound to a Plastic Substrate in Surface Water. J. Glob. Antimicrob. Resist..

[52] Li Y.-Q., Zhang C.-M., Yuan Q.-Q., Wu K. (2023). New Insight into the Effect of Microplastics on Antibiotic Resistance and Bacterial Community of Biofilm. Chemosphere.

[53] Yan W., Bai R., Zhang Q., Jiang Y., Chen G., Zhang Y., Wu Y., Guo X., Xiao Y., Zhao F. (2024). Metagenomic Insights into Ecological Risk of Antibiotic Resistome and Mobilome in Riverine Plastisphere under Impact of Urbanization. Environ. Int..

[54] Sabatino R., Zullo R., Di Cesare A., Piscia R., Musazzi S., Corno G., Volta P., Galafassi S. (2024). Traditional and Biodegradable Plastics Host Distinct and Potentially More Hazardous Microbes When Compared to Both Natural Materials and Planktonic Community. J. Hazard. Mater..

[55] Berglund F., Österlund T., Boulund F., Marathe N.P., Larsson D.G.J., Kristiansson E. (2019). Identification and Reconstruction of Novel Antibiotic Resistance Genes from Metagenomes. Microbiome.

[56] Liu S., Cao J., Yu J., Jian M., Zou L. (2024). Microplastics Exacerbate the Ecological Risk of Antibiotic Resistance Genes in Wetland Ecosystem. J. Environ. Manage..

[57] Zhang W., Geng J., Sun M., Jiang C., Lin H., Chen H., Yang Y. (2024). Distinct Species Turnover Patterns Shaped the Richness of Antibiotic Resistance Genes on Eight Different Microplastic Polymers. Environ. Res..

[58] Ferheen I., Spurio R., Marcheggiani S. (2024). Emerging Issues on Antibiotic-Resistant Bacteria Colonizing Plastic Waste in Aquatic Ecosystems. Antibiotics.

[59] Chen Y., Yan Z., Zhou Y., Zhang Y., Jiang R., Wang M., Yuan S., Lu G. (2024). Dynamic Evolution of Antibiotic Resistance Genes in Plastisphere in the Vertical Profile of Urban Rivers. Water Res..

[60] Chen Y., Liu S., Ouyang T., Jiang R., Ma J., Lu G., Yuan S., Yan Z. (2025). Reshaping the Antibiotic Resistance Genes in Plastisphere upon Deposition in Sediment-Water Interface: Dynamic Evolution and Propagation Mechanism. J. Hazard. Mater..

[61] Thao L.T., Hien V.T.T., Tram N.T., Hieu V.H., Gutierrez T., Thi Thu Ha H., Dung P.M., Thi Thuy Huong N. (2024). First Evidence of Microplastic-Associated Extended-Spectrum Beta-Lactamase (ESBL)-Producing Bacteria in the Red River Delta, Vietnam. J. Hazard. Mater. Lett..

[62] Tacconelli E., Carrara E., Savoldi A., Harbarth S., Mendelson M., Monnet D.L., Pulcini C., Kahlmeter G., Kluytmans J., Carmeli Y. (2018). Discovery, Research, and Development of New Antibiotics: The WHO Priority List of Antibiotic-Resistant Bacteria and Tuberculosis. Lancet Infect. Dis..

[63] Zhang A.-N., Gaston J.M., Dai C.L., Zhao S., Poyet M., Groussin M., Yin X., Li L.-G., van Loosdrecht M.C.M., Topp E. (2021). An Omics-Based Framework for Assessing the Health Risk of Antimicrobial Resistance Genes. Nat. Commun..

[64] Hu Z., Wang W., Wang J., Xiao Y., Shi J., Wang C., Mai W., Li G., An T. (2024). Selective Enrichment of High-Risk Antibiotic Resistance Genes and Priority Pathogens in Freshwater Plastisphere: Unique Role of Biodegradable Microplastics. J. Hazard. Mater..

[65] Zhou M., Luo C., Zhang J., Li R., Chen J., Ren P., Tang Y., Suo Z., Chen K. (2025). Potential Risk of Microplastics in Plateau Karst Lakes: Insights from Metagenomic Analysis. Environ. Res..

[66] Shu J., Hou S., Cao H., Liu X., Cai W., Zeng Y., Luo X., Tu W., Zhang Y., Zhao C. (2025). Spread Performance and Underlying Mechanisms of Pathogenic Bacteria and Antibiotic Resistance Genes Adhered on Microplastics in the Sediments of Different Urban Water Bodies. Environ. Pollut..

[67] Luo G., Fan L., Liang B., Guo J., Gao S.-H. (2025). Determining Antimicrobial Resistance in the Plastisphere: Lower Risks of Nonbiodegradable vs. Higher Risks of Biodegradable Microplastics. Environ. Sci. Technol..

[68] Liu Y., Liu W., Yang X., Wang J., Lin H., Yang Y. (2021). Microplastics Are a Hotspot for Antibiotic Resistance Genes: Progress and Perspective. Sci. Total Environ..

[69] Wang Y., Liu X., Huang C., Han W., Gu P., Jing R., Yang Q. (2025). Antibiotic Resistance Genes and Virulence Factors in the Plastisphere in Wastewater Treatment Plant Effluent: Health Risk Quantification and Driving Mechanism Interpretation. Water Res..

[70] Akdogan Z., Guven B. (2019). Microplastics in the Environment: A Critical Review of Current Understanding and Identification of Future Research Needs. Environ. Pollut..

[71] Zhang X., Wang J., Yang Z., Zhang Z., Wang M., Zhang T., Chen Y., Wu X., Liu P., Jia H. (2025). Microplastics Exacerbated Conjugative Transfer of Antibiotic Resistance Genes during Ultraviolet Disinfection: Highlighting Difference between Conventional and Biodegradable Ones. Environ. Sci. Technol..

[72] Woodford L., Messer L.F., Ormsby M.J., White H.L., Fellows R., Quilliam R.S. (2025). Exploiting Microplastics and the Plastisphere for the Surveillance of Human Pathogenic Bacteria Discharged into Surface Waters in Wastewater Effluent. Water Res..

[73] Eckert E.M., Di Cesare A., Kettner M.T., Arias-Andres M., Fontaneto D., Grossart H.-P., Corno G. (2018). Microplastics Increase Impact of Treated Wastewater on Freshwater Microbial Community. Environ. Pollut..

[74] Wang J., Peng C., Dai Y., Li Y., Jiao S., Ma X., Liu X., Wang L. (2022). Slower Antibiotics Degradation and Higher Resistance Genes Enrichment in Plastisphere. Water Res..

[75] Zadjelovic V., Wright R.J., Borsetto C., Quartey J., Cairns T.N., Langille M.G.I., Wellington E.M.H., Christie-Oleza J.A. (2023). Microbial Hitchhikers Harbouring Antimicrobial-Resistance Genes in the Riverine Plastisphere. Microbiome.

[76] Perveen S., Pablos C., Reynolds K., Stanley S., Marugán J. (2023). Growth and Prevalence of Antibiotic-Resistant Bacteria in Microplastic Biofilm from Wastewater Treatment Plant Effluents. Sci. Total Environ..

[77] Silva I., Rodrigues E.T., Tacão M., Henriques I. (2023). Microplastics Accumulate Priority Antibiotic-Resistant Pathogens: Evidence from the Riverine Plastisphere. Environ. Pollut..

[78] Silva I., Tacão M., Henriques I. (2025). Wastewater Discharges and Polymer Type Modulate the Riverine Plastisphere and Set the Role of Microplastics as Vectors of Pathogens and Antibiotic Resistance. J. Water Proc. Eng..

[79] Martínez-Campos S., González-Pleiter M., Rico A., Schell T., Vighi M., Fernández-Piñas F., Rosal R., Leganés F. (2023). Time-Course Biofilm Formation and Presence of Antibiotic Resistance Genes on Everyday Plastic Items Deployed in River Waters. J. Hazard. Mater..

[80] Eytcheson S.A., Brown S.A., Wu H., Nietch C.T., Weaver P.C., Darling J.A., Pilgrim E.M., Purucker S.T., Molina M. (2025). Assessment of Emerging Pathogens and Antibiotic Resistance Genes in the Biofilm of Microplastics Incubated under a Wastewater Discharge Simulation. Environ. Microbiol..

[81] Malla M.A., Nomalihle M., Featherston J., Kumar A., Amoah I.D., Ismail A., Bux F., Kumari S. (2025). Comprehensive Profiling and Risk Assessment of Antibiotic Resistomes in Surface Water and Plastisphere by Integrated Shotgun Metagenomics. J. Hazard. Mater..

[82] Zhang Q., Fan Y., Qian X., Zhang Y. (2025). Unraveling the Role of Microplastics in Antibiotic Resistance: Insights from Long-Read Metagenomics on ARG Mobility and Host Dynamics. J. Hazard. Mater..

[83] Ni N., Qiu J., Ge W., Guo X., Zhu D., Wang N., Luo Y. (2025). Fibrous and Fragmented Microplastics Discharged from Sewage Amplify Health Risks Associated with Antibiotic Resistance Genes in Aquatic Environments. Environ. Sci. Technol..

[84] Yang Y., Liu G., Song W., Ye C., Lin H., Li Z., Liu W. (2019). Plastics in the Marine Environment Are Reservoirs for Antibiotic and Metal Resistance Genes. Environ. Int..

[85] Radisic V., Nimje P.S., Bienfait A.M., Marathe N.P. (2020). Marine Plastics from Norwegian West Coast Carry Potentially Virulent Fish Pathogens and Opportunistic Human Pathogens Harboring New Variants of Antibiotic Resistance Genes. Microorganisms.

[86] Sucato A., Vecchioni L., Savoca D., Presentato A., Arculeo M., Alduina R. (2021). A Comparative Analysis of Aquatic and Polyethylene-Associated Antibiotic-Resistant Microbiota in the Mediterranean Sea. Biology.

[87] Vlaanderen E.J., Ghaly T.M., Moore L.R., Focardi A., Paulsen I.T., Tetu S.G. (2023). Plastic Leachate Exposure Drives Antibiotic Resistance and Virulence in Marine Bacterial Communities. Environ. Pollut..

[88] Liang H., de Haan W.P., Cerdà-Domènech M., Méndez J., Lucena F., García-Aljaro C., Sanchez-Vidal A., Ballesté E. (2023). Detection of Faecal Bacteria and Antibiotic Resistance Genes in Biofilms Attached to Plastics from Human-Impacted Coastal Areas. Environ. Pollut..

[89] Kim H., Yoo K. (2024). Marine Plastisphere Selectively Enriches Microbial Assemblages and Antibiotic Resistance Genes during Long-Term Cultivation Periods. Environ. Pollut..

[90] Di Cesare A., Sathicq M.B., Sbaffi T., Sabatino R., Manca D., Breider F., Coudret S., Pinnell L.J., Turner J.W., Corno G. (2024). Parity in Bacterial Communities and Resistomes: Microplastic and Natural Organic Particles in the Tyrrhenian Sea. Mar. Pollut. Bull..

[91] Sababadichetty L., Miltgen G., Vincent B., Guilhaumon F., Lenoble V., Thibault M., Bureau S., Tortosa P., Bouvier T., Jourand P. (2024). Microplastics in the Insular Marine Environment of the Southwest Indian Ocean Carry a Microbiome Including Antimicrobial Resistant (AMR) Bacteria: A Case Study from Reunion Island. Mar. Pollut. Bull..

[92] Zhou Z., Tang J., Tang K., An M., Liu Z., Wu Z., Cao X., He C. (2024). Selective Enrichment of Bacteria and Antibiotic Resistance Genes in Microplastic Biofilms and Their Potential Hazards in Coral Reef Ecosystems. Chemosphere.

[93] Hassen W., Danioux A., Oueslati A., Santana-Rodríguez J.J., Sire O., Sedrati M., Ben Mansour H. (2024). Dissemination of Antibiotic-Resistant Bacteria Associated with Microplastics Collected from Monastir and Mahdia Coasts (Tunisia). Microb. Pathog..

[94] Caruso G., Azzaro M., Dell’Acqua O., Papale M., Lo Giudice A., Laganà P. (2024). Plastic Polymers and Antibiotic Resistance in an Antarctic Environment (Ross Sea): Are We Revealing the Tip of an Iceberg?. Microorganisms.

[95] Maghsodian Z., Sanati A.M., Ramavandi B., Ghasemi A., Sorial G.A. (2021). Microplastics Accumulation in Sediments and *Periophthalmus waltoni* Fish, Mangrove Forests in Southern Iran. Chemosphere.

[96] Sun R., He L., Li T., Dai Z., Sun S., Ren L., Liang Y.-Q., Zhang Y., Li C. (2022). Impact of the Surrounding Environment on Antibiotic Resistance Genes Carried by Microplastics in Mangroves. Sci. Total Environ..

[97] Sun R., Li T., Qiu S., Liu Y., Wu Z., Dai Z., Liao Y., Chen X., Chen S., Li C. (2023). Occurrence of Antibiotic Resistance Genes Carried by Plastic Waste from Mangrove Wetlands of the South China Sea. Sci. Total Environ..

[98] Li H.-Q., Wang W.-L., Shen Y.-J., Su J.-Q. (2025). Mangrove Plastisphere as a Hotspot for High-Risk Antibiotic Resistance Genes and Pathogens. Environ. Res..

[99] Guo X.-P., Sun X.-L., Chen Y.-R., Hou L., Liu M., Yang Y. (2020). Antibiotic Resistance Genes in Biofilms on Plastic Wastes in an Estuarine Environment. Sci. Total Environ..

[100] Di Cesare A., Pinnell L.J., Brambilla D., Elli G., Sabatino R., Sathicq M.B., Corno G., O’Donnell C., Turner J.W. (2021). Bioplastic Accumulates Antibiotic and Metal Resistance Genes in Coastal Marine Sediments. Environ. Pollut..

[101] Peng R., Xu Y., Li R., Wang W., Wang H., Zhang X., Yuan Q. (2024). Marine Microplastics Enrich Antibiotic Resistance Genes (ARGs), Especially Extracellular ARGs: An Investigation in the East China Sea. Mar. Pollut. Bull..

[102] Zhao X., Niu Z., Ma Y., Zhang Y., Li Y., Zhang R. (2024). Exploring the Dynamics of Antibiotic Resistome on Plastic Debris Traveling from the River to the Sea along a Representative Estuary Based on Field Sequential Transfer Incubations. Sci. Total Environ..

[103] Yashir N., Sun Q., Zhang X., Ma M., Wang D., Feng Y., Song X. (2025). Co-Occurrence of Microplastics, PFASs, Antibiotics, and Antibiotic Resistance Genes in Groundwater and Their Composite Impacts on Indigenous Microbial Communities: A Field Study. Sci. Total Environ..

[104] Guruge K.S., Goswami P., Kanda K., Abeynayaka A., Kumagai M., Watanabe M., Tamamura-Andoh Y. (2024). Plastiome: Plastisphere-Enriched Mobile Resistome in Aquatic Environments. J. Hazard. Mater..

[105] Lin W., Cao S., Wu Q., Xu F., Li R., Cui L. (2024). Size Effects of Microplastics on Antibiotic Resistome and Core Microbiome in an Urban River. Sci. Total Environ..

[106] Yang K., Xu F., Xing X., Wei J., Chen Q.-L., Su J.-Q., Cui L. (2025). Microplastics Pose an Elevated Antimicrobial Resistance Risk than Natural Surfaces via a Systematic Comparative Study of Surface Biofilms in Rivers. Environ. Sci. Technol..

[107] Li R., Zhu L., Cui L., Zhu Y.-G. (2022). Viral Diversity and Potential Environmental Risk in Microplastic at Watershed Scale: Evidence from Metagenomic Analysis of Plastisphere. Environ. Int..

[108] Ma Y., Dong X., Sun Y., Li B., Ma H., Li H., Zhao X., Ran S., Zhang J., Ye Y. (2025). Diversity and Functional Roles of Viral Communities in Gene Transfer and Antibiotic Resistance in Aquaculture Waters and Microplastic Biofilms. Environ. Pollut..

[109] Xia R., Yin X., Balcazar J.L., Huang D., Liao J., Wang D., Alvarez P.J.J., Yu P. (2025). Bacterium-Phage Symbiosis Facilitates the Enrichment of Bacterial Pathogens and Antibiotic-Resistant Bacteria in the Plastisphere. Environ. Sci. Technol..

[110] EFSA Panel on Contaminants in the Food Chain (CONTAM) (2016). Presence of Microplastics and Nanoplastics in Food, with Particular Focus on Seafood. EFSA J..

[111] Jangid H., Dutta J., Karnwal A., Kumar G. (2025). Microplastic Contamination in Fish: A Systematic Global Review of Trends, Health Risks, and Implications for Consumer Safety. Mar. Pollut. Bull..

[112] Han Y., Zhou W., Tang Y., Shi W., Shao Y., Ren P., Zhang J., Xiao G., Sun H., Liu G. (2021). Microplastics Aggravate the Bioaccumulation of Three Veterinary Antibiotics in the Thick Shell Mussel *Mytilus coruscus* and Induce Synergistic Immunotoxic Effects. Sci. Total Environ..

[113] Song K., Wang R., Yang G., Xie S., Chen Y., Yang F., Huang W., Zhang T., Feng Z. (2023). Pollution Concerns in Mariculture Water and Cultured Economical Bivalves: Occurrence of Microplastics under Different Aquaculture Modes. J. Clean. Prod..

[114] Su H., Xu W., Hu X., Xu Y., Wen G., Cao Y. (2025). The Impact of Microplastics on Antibiotic Resistance Genes, Metal Resistance Genes, and Bacterial Community in Aquaculture Environment. J. Hazard. Mater..

[115] Gong W., Guo L., Huang C., Xie B., Jiang M., Zhao Y., Zhang H., Wu Y., Liang H. (2024). A Systematic Review of Antibiotics and Antibiotic Resistance Genes (ARGs) in Mariculture Wastewater: Antibiotics Removal by Microalgal-Bacterial Symbiotic System (MBSS), ARGs Characterization on the Metagenomic. Sci. Total Environ..

[116] Yu X., Du H., Huang Y., Yin X., Liu Y., Li Y., Liu H., Wang X. (2022). Selective Adsorption of Antibiotics on Aged Microplastics Originating from Mariculture Benefits the Colonization of Opportunistic Pathogenic Bacteria. Environ. Pollut..

[117] Lu J., Zhang Y., Wu J., Luo Y. (2019). Effects of Microplastics on Distribution of Antibiotic Resistance Genes in Recirculating Aquaculture System. Ecotoxicol. Environ. Saf..

[118] Zhang Y., Lu J., Wu J., Wang J., Luo Y. (2020). Potential Risks of Microplastics Combined with Superbugs: Enrichment of Antibiotic Resistant Bacteria on the Surface of Microplastics in Mariculture System. Ecotoxicol. Environ. Saf..

[119] Lu J., Wu J., Wang J. (2022). Metagenomic Analysis on Resistance Genes in Water and Microplastics from a Mariculture System. Front. Environ. Sci. Eng..

[120] Xiao S., Zhang Y., Wu Y., Li J., Dai W., Pang K., Liu Y., Wu R. (2023). Bacterial Community Succession and the Enrichment of Antibiotic Resistance Genes on Microplastics in an Oyster Farm. Mar. Pollut. Bull..

[121] Antimicrobial Resistance Collaborators (2022). Global Burden of Bacterial Antimicrobial Resistance in 2019: A Systematic Analysis. Lancet.

[122] Jia J., Liu Q., Wu C. (2023). Microplastic and Antibiotic Proliferated the Colonization of Specific Bacteria and Antibiotic Resistance Genes in the Phycosphere of *Chlorella Pyrenoidosa*. J. Hazard. Mater..

[123] Naudet J., d’Orbcastel E.R., Bouvier T., Godreuil S., Dyall S., Bouvy S., Rieuvilleneuve F., Restrepo-Ortiz C.X., Bettarel Y., Auguet J.-C. (2023). Identifying Macroplastic Pathobiomes and Antibiotic Resistance in a Subtropical Fish Farm. Mar. Pollut. Bull..

[124] Xie S., Peng L., Zhou Z., Xu N., Li S., Feng Y. (2025). Biodegradable Microplastics Amplify Antibiotic Resistance in Aquaculture: A Potential One Health Crisis from Environment to Seafood. J. Hazard. Mater..

[125] Yu Z., Zhang L., Huang Q., Dong S., Wang X., Yan C. (2022). Combined Effects of Micro-/Nano-Plastics and Oxytetracycline on the Intestinal Histopathology and Microbiome in Zebrafish (*Danio rerio*). Sci. Total Environ..

[126] Zhang P., Lu G., Sun Y., Yan Z., Dang T., Liu J. (2022). Metagenomic Analysis Explores the Interaction of Aged Microplastics and Roxithromycin on Gut Microbiota and Antibiotic Resistance Genes of *Carassius auratus*. J. Hazard. Mater..

[127] Zhang P., Lu G., Sun Y., Zhang J., Liu J., Yan Z. (2022). Aged Microplastics Change the Toxicological Mechanism of Roxithromycin on *Carassius auratus*: Size-Dependent Interaction and Potential Long-Term Effects. Environ. Int..

[128] Zhao P., Lu W., Avellán-Llaguno R.D., Liao X., Ye G., Pan Z., Hu A., Huang Q. (2023). Gut Microbiota Related Response of *Oryzias melastigma* to Combined Exposure of Polystyrene Microplastics and Tetracycline. Sci. Total Environ..

[129] Yu Z., Qiu D., Zhou T., Zeng L., Yan C. (2024). Biofilm Enhances the Interactive Effects of Microplastics and Oxytetracycline on Zebrafish Intestine. Aquat. Toxicol..

[130] Li W., Zeng J., Zheng N., Ge C., Li Y., Yao H. (2024). Polyvinyl Chloride Microplastics in the Aquatic Environment Enrich Potential Pathogenic Bacteria and Spread Antibiotic Resistance Genes in the Fish Gut. J. Hazard. Mater..

[131] Milani G., Cortimiglia C., Belloso Daza M.V., Greco E., Bassi D., Cocconcelli P.S. (2024). Microplastic-Mediated Transfer of Tetracycline Resistance: Unveiling the Role of Mussels in Marine Ecosystems. Antibiotics.

[132] Li J., You L., Gin K.Y.-H., He Y. (2024). Impact of Microplastics Pollution on Ciprofloxacin Bioaccumulation in the Edible Mussel (*Perna viridis*): Implications for Human Gut Health Risks. Environ. Technol. Innov..

[133] Zhou W., Han Y., Tang Y., Shi W., Du X., Sun S., Liu G. (2020). Microplastics Aggravate the Bioaccumulation of Two Waterborne Veterinary Antibiotics in an Edible Bivalve Species: Potential Mechanisms and Implications for Human Health. Environ. Sci. Technol..

[134] Ma J., Lv C., Gong Z., Zhang K., Wang S., Li R., Chen K., Zhu F., Wang D., Qiu Z. (2025). Promotion of Microplastic Degradation on the Conjugative Transfer of Antibiotic Resistance Genes in the Gut of Macrobenthic Invertebrates. Ecotoxicol. Environ. Saf..

[135] Liang X., Li B., Dong X., Zhao X., Li H., Ye Y., Ma H., Ran S., Li J. (2025). Impact of Microplastics Exposure on the Reconfiguration of Viral Community Structure and Disruption of Ecological Functions in the Digestive Gland of *Mytilus Coruscus*. J. Hazard. Mater..

[136] Yu Y., Qian J., Wang W., Xie M., Tan Q.-G., Chen R. (2025). Effects of Aged Microplastics on the Abundance of Antibiotic Resistance Genes in Oysters and Their Excreta. Mar. Environ. Res..

[137] Zhang P., Lu G., Sun Y., Yan Z., Zhang L., Liu J. (2024). Effect of Microplastics on Oxytetracycline Trophic Transfer: Immune, Gut Microbiota and Antibiotic Resistance Gene Responses. J. Hazard. Mater..

[138] Pitiot A., Rolin C., Seguin-Devaux C., Zimmer J. (2025). Fighting Antibiotic Resistance: Insights into Human Barriers and New Opportunities: Antibiotic Resistance Constantly Rises with the Development of Human Activities. We Discuss Barriers and Opportunities to Get It under Control. Bioessays.

[139] Husna A., Rahman M.M., Badruzzaman A.T.M., Sikder M.H., Islam M.R., Rahman M.T., Alam J., Ashour H.M. (2023). Extended-Spectrum β-Lactamases (ESBL): Challenges and Opportunities. Biomedicines.

[140] Zhang S., Cui L., Zhao Y., Xie H., Song M., Wu H., Hu Z., Liang S., Zhang J. (2024). The Critical Role of Microplastics in the Fate and Transformation of Sulfamethoxazole and Antibiotic Resistance Genes within Vertical Subsurface-Flow Constructed Wetlands. J. Hazard. Mater..

[141] Li H., Shen M., Li M., Tao S., Li T., Yang Z. (2024). Removal of Microplastics and Resistance Genes in Livestock and Aquaculture Wastewater: Current Knowledge and Future Directions. J. Environ. Chem. Eng..

[142] Cholewińska P., Wojnarowski K., Szeligowska N., Pokorny P., Hussein W., Hasegawa Y., Dobicki W., Palić D. (2025). Presence of Microplastic Particles Increased Abundance of Pathogens and Antimicrobial Resistance Genes in Microbial Communities from the Oder River Water and Sediment. Sci. Rep..

